# Long-term Multimodal Recording Reveals Epigenetic Adaptation Routes in Dormant Breast Cancer Cells

**DOI:** 10.1158/2159-8290.CD-23-1161

**Published:** 2024-03-26

**Authors:** Dalia Rosano, Emre Sofyali, Heena Dhiman, Chiara Ghirardi, Diana Ivanoiu, Timon Heide, Andrea Vingiani, Alessia Bertolotti, Giancarlo Pruneri, Eleonora Canale, Hannah F. Dewhurst, Debjani Saha, Neil Slaven, Iros Barozzi, Tong Li, Grigory Zemlyanskiy, Henry Phillips, Chela James, Balázs Győrffy, Claire Lynn, George D. Cresswell, Farah Rehman, Roberta Noberini, Tiziana Bonaldi, Andrea Sottoriva, Luca Magnani

**Affiliations:** 1 Department of Surgery and Cancer, Imperial College London, London, United Kingdom.; 2 The Breast Cancer Now Toby Robins Research Center, The Institute of Cancer Research, London, United Kingdom.; 3 Department of Experimental Oncology, IEO, European Institute of Oncology IRCCS, Milan, Italy.; 4 Human Technopole, Milan, Italy.; 5 Centre for Evolution and Cancer, Institute of Cancer Research, London, United Kingdom.; 6 Istituto Nazionale Tumori, Milan, Italy.; 7 Department of Oncology and Haematology-Oncology, University of Milano, Milano, Italy.; 8 Environmental Genomics and Systems Biology Division, Lawrence Berkeley National Laboratory, Berkeley.; 9 Centre for Cancer Research, Medical University of Vienna, Austria.; 10 Department of Bioinformatics, Semmelweis University, Budapest, Hungary.; 11 RCNS Cancer Biomarker Research Group, Budapest, Hungary.; 12 Department of Biophysics, Medical School, University of Pecs, Pecs, Hungary.; 13 Charing Cross Hospital, Imperial College NHS Trust, London, United Kingdom.

## Abstract

Patients with estrogen receptor–positive breast cancer receive adjuvant endocrine therapies (ET) that delay relapse by targeting clinically undetectable micrometastatic deposits. Yet, up to 50% of patients relapse even decades after surgery through unknown mechanisms likely involving dormancy. To investigate genetic and transcriptional changes underlying tumor awakening, we analyzed late relapse patients and longitudinally profiled a rare cohort treated with long-term neoadjuvant ETs until progression. Next, we developed an *in vitro* evolutionary study to record the adaptive strategies of individual lineages in unperturbed parallel experiments. Our data demonstrate that ETs induce nongenetic cell state transitions into dormancy in a stochastic subset of cells via epigenetic reprogramming. Single lineages with divergent phenotypes awaken unpredictably in the absence of recurrent genetic alterations. Targeting the dormant epigenome shows promising activity against adapting cancer cells. Overall, this study uncovers the contribution of epigenetic adaptation to the evolution of resistance to ETs.

**Significance:**

This study advances the understanding of therapy-induced dormancy with potential clinical implications for breast cancer. Estrogen receptor-positive breast cancer cells adapt to endocrine treatment by entering a dormant state characterized by strong heterochromatinization with no recurrent genetic changes. Targeting the epigenetic rewiring impairs the adaptation of cancer cells to ETs.

*See related commentary by Llinas-Bertran et al., p. 704.*

*This article is featured in Selected Articles from This Issue, p. 695*

## Teaser

Epigenetic reprogramming underlies dormancy after endocrine treatment in estrogen receptor–positive breast cancer, and targeting the epigenome during this adaptation has potential clinical implications.

## INTRODUCTION

Understanding cancer cell dormancy is a crucial challenge in cancer research considering the potential contribution of dormancy to tumor relapse, therapy resistance, and immune evasion (1, 2). Tumor relapse is thought to begin with the “awakening” of dormant persister cells within undetectable microdisseminated reservoirs (3–5). Tumor recurrence can occur over the course of 25 years after initial diagnosis (6). Different from other cancer types, relapse events in patients with estrogen receptor–positive (ER^+^) breast cancer do not decline with time (7), do not spike at the end of adjuvant ETs (8), nor are significantly influenced by the size of the tumor (9, 10). Collectively, these data indicate that the molecular events driving cells out from dormancy might involve reversible cell state transitions and led us to hypothesize that targetable epigenetic processes (11–13) triggered by adjuvant ETs might fuel adaptation and evolution in patients with ER^+^ breast cancer.

## RESULTS

### Genomic Profiling of Awakening in Patients with ER^+^ Breast Cancer

**Figure 1.**
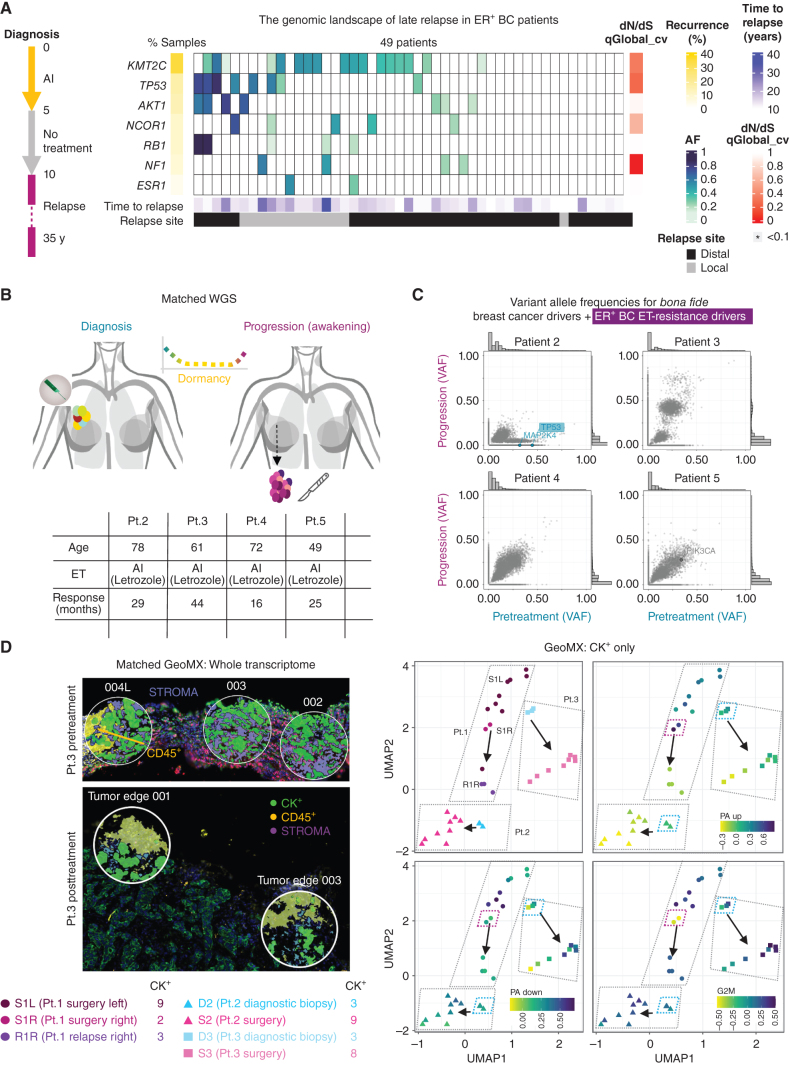
Genetic profile of tumor awakening in the clinical setting. **A,** High-depth profiling (median 105.47×) of ER^+^ breast cancer (BC; estrogen receptor–positive breast cancer) late relapses using a custom targeted panel. The simplified treatment scheme of patients is shown on the left. The heat map shows the mutations in ET resistance drivers in ER^+^ breast cancer passing the filters for allele depth ≥20, Alternate F1R2 + F2R1 ≥4, allele frequency ≥0.1, and consequence level of moderate or high. Time to relapse (years), recurrence in the data set, allele frequency, and relapse site are indicated. Significant genes are indicated based on dN/dS analysis from the q-value of neutrality test at the gene level (*qglobal_cv ≤0.1). **B,** Clinical histories of patients 2–5. The table shows age and response time to ET for each patient (letrozole). **C,** Scatter plots of VAF from whole-genome sequencing (WGS) data. Pairwise comparisons were done for pretreatment (diagnostic biopsies) versus progression (surgical biopsies). All patients were managed with primary endocrine therapy until progression. Labeled genes passed two filters: *bona fide* breast cancer drivers and ET resistance drivers in ER^+^ breast cancer and FATHMM significant score >0.6 (predicted damaging). Detected variants are labeled and color-coded according to detection at diagnosis (teal), progression (magenta), or both (gray). The highlighted gene (*TP53*) is annotated as a variant detected in ET resistance drivers in ER^+^ breast cancer according to the comprehensive ET-resistance driver gene list compiled based on Bertucci et al. (14). Marginal histograms of VAFs are shown on the sides of each plot. **D,** Spatial transcriptomics analysis of patients 1–3. On the left, representative images of regions of interest (ROI) from patient 3, pre- and post-treatment, are shown with the relevant staining. Green, pan cytokeratin (CK^+^); yellow, immune cells (CD45^+^); purple, stroma. On the right side, GeoMx UMAPs of previously identified pre-adapted SWNE up and down signatures from (2), and G_2_–M checkpoint signatures are shown for patients 1–3 (CK^+^ segment). D1 L biopsy was not suitable for spatial transcriptomics analysis due to poor specimen quality and was excluded from further examinations. S1L: surgical biopsy in the left breast; S1R: surgical biopsy in the right breast; R1R: loco-regional relapse after surgery in the right breast.

It is currently unclear if exit from dormancy, defined as long-term tumor quiescence, is mediated by genetic events. Molecular profiling of clinical relapse (local or metastatic) has identified a small number of recurrent genetic events only in 20% to 40% of patients with advanced ER^+^ breast cancer (i.e., *ESR1* activating mutations; refs. 14–16). Because the prospective cohorts profiled in these studies were naturally enriched for early relapse (17), we identified a unique cohort of late relapses (median time to relapse 13 years, min–max 10–35 years, *n* = 49) and profiled them with a targeted coding panel (18). Our data show that late relapses are enriched in *KMT2C* (an H3K4 methyltransferase) mutations while being surprisingly depleted of *ESR1* activating mutations, suggesting that awakening from long-term dormancy is not driven by classic genetic drivers of early relapse (Fig. 1A; Supplementary Fig. S1A and S1B; Supplementary Table S1).

Longitudinal genomic profiling inferred causal genetic events (often not from the same patient) from cancer cells that have undergone extensive replication, often in distinct anatomic sites (14–16) before they become detectable by imaging (19). We bypassed these confounding factors by focusing on a rare cohort of patients (*n* = 5) managed exclusively with ET until *in situ* progression (tumor expansion evaluated by radiologic examination) and resampled at the same anatomic location at diagnosis (diagnostic biopsy) and progression (surgical biopsy). Patient 1 presented with bilateral ER^+^ breast cancer characterized by marked histologic heterogeneity (Supplementary Fig. S2A and S2B). Radiologic examination showed partial response in both lesions, followed by >6 months of stable residual disease (i.e., putative dormancy) followed by radiologic progression (awakening) in the left breast (S1L, 21 months; Supplementary Fig. S2C). Both diagnostic (pretreatment) and surgical biopsies (progression) were profiled by high-depth whole-genome sequencing (WGS; Supplementary Table S1). In this work, we define potential genetic or nongenetic hits associated with resistance as preexistent when identified pretreatment or *de novo* if identified posttreatment. Although we could identify *de novo* pathogenic single-nucleotide variations (SNV) in *bona fide* breast cancer drivers [Functional analysis through Hidden Markov Models (FATHMM) >0.6; refs. 20, 21], none has been previously linked to ET resistance (i.e., FGFR2 S702 L; refs. 14–16; Supplementary Fig. S3A and Supplementary Table S1; refs. 15–17). Thirty-six months after bilateral surgery, the patient experienced a loco-regional recurrence in the right breast (R1R) characterized by *de novo* mutations in *ESR1* (D538G; ref. 16) and *FGFR2* (V565 L; ref. 16) genes, potentially explaining resistance to the aromatase inhibitor (AI)–FGFRi combination (Supplementary Fig. S3A and Supplementary Table S1). Careful examination of genomic data did not find evidence of *ESR1* (D538G) or *FGFR2* (V565L) mutations at progression (S1 L and S1R, 63X and 69X base coverage, respectively; Supplementary Table S2), meaning that these hits likely happened between progression and relapse or that progression and relapse are driven by different disseminated clones. Genomic characterization of four analogous clinical cases also failed to identify preexistent or *de novo* SNVs explicitly linked to ET resistance at the time of tumor progression *in situ* (awakening; Fig. 1B and C; and Supplementary Table S1). Extending the analysis to *bona fide* genetic drivers of other tumor types (21) did not result in additional candidates (Supplementary Fig. S3B and S3C; Supplementary Table S1).

Next, we sought to characterize if progression was driven by recurrent changes in gene expression (Fig. 1D; Supplementary Figs. S4 and S5). Comparing pretreatment and progression samples using spatial transcriptomics did not reveal consistent changes in the immune component (Supplementary Fig. S6). Conversely, focusing on cancer-intrinsic changes identified potential transcriptional evolution between pretreatment and awakened lesions (Fig. 1D; Supplementary Fig. S7A and S7B). Interestingly, transcriptional heterogeneity between individual regions appears to increase posttreatment. A stark example was captured in patient 1 with some areas exhibiting features of dormancy (i.e., dormancy signature derived from ref. 2; Supplementary Table S3) despite overall progression (S1R, Fig. 1D). These data suggest that adaptation to therapy is not driven by preexistent or *de novo* SNVs but involves divergent transcriptional reprogramming within individual tumors.

### Tumor Awakening Dynamics Are Unpredictable *In Vitro*

**Figure 2.**
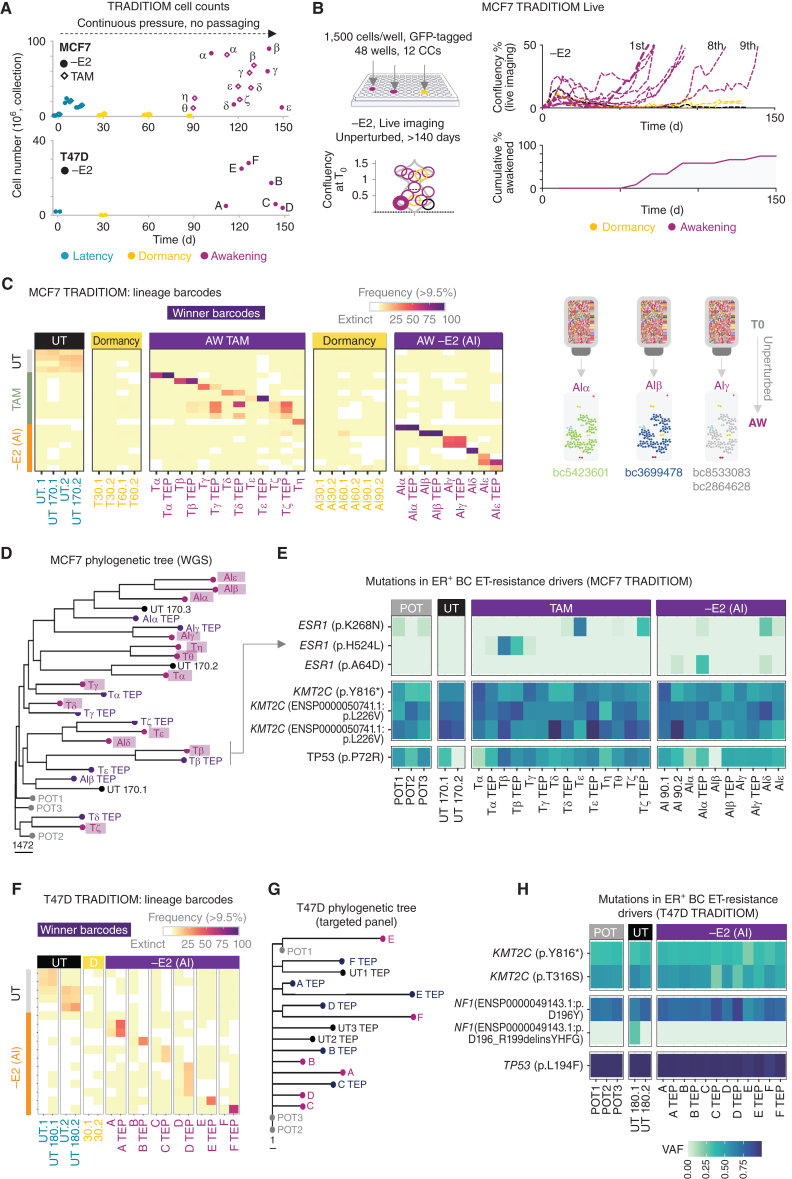
TRADITIOM genetic analysis and lineage composition. **A,** Cell counts for MCF7 and T47D HYPERflasks for −E2 (circle) and TAM (diamond) arms at their respective time of collection (teal: latency–time between the onset of the treatment and cell cycle arrest in the whole cell population; yellow: dormancy, magenta: awakening, early progression. α−ζ: MCF7 awakening carbon copies, **A–F**: T47D awakening carbon copies). **B,** TRADITIOM Live set-up: 12 replicates were seeded in a 48-well plate and imaged two times a week for 150 days using Incucyte Zoom (9 scanning windows per well) to monitor awakening dynamics. Awakening was defined as wells reaching a confluency of 50%. Minor differences in initial plating (violin plot) do not predict awakening times. **C,** Heatmap of MCF7 high-frequency barcodes (frequency ≥10%) among UT (untreated) samples’ endpoints and TRADITIOM carbon copies (replicates) for both TAM and –E2 arm (AI) at the time of dormancy and awakening (T30–60, AI30–60: dormancy time points, cells treated for 30 or 60 days with tamoxifen or estrogen deprivation, respectively). **D,** Phylogenetic tree for MCF7 TRADITIOM samples based on SNVs and indels (VAF >10%). Data depict TRADITIOM samples from POT (pretreatment), awakenings (early progression), and TEPs (late progression) of both treatment arms [TAM and AI (–E2)] and their untreated counterpart samples (UT) cultured in parallel for 170 days. **E,** VAF heatmap for ET resistance driver genes in ER^+^ breast cancer, compiled based on Bertucci et al. (14) derived from TRADITIOM WGS, illustrating only variants with significant changes in VAF across samples (Fisher exact test, *P* < 0.01). **F,** Heatmap of T47D high frequency barcodes (frequency ≥10%) among UT (untreated) samples’ endpoints and TRADITIOM carbon copies (replicates) for –E2 (AI) arm at the time of dormancy and awakening. **G,** Phylogenetic tree for T47D TRADITIOM samples based on SNVs and indels (derived from the targeted sequencing using a custom targeted panel, VAF >10%). **H,** VAF heatmap for ET resistance driver genes in ER^+^ breast cancer, compiled based on Bertucci et al. (14) derived from the targeted sequencing using a custom targeted panel, illustrating only variants with significant changes in VAF across samples (Fisher exact test, *P* < 0.01). Data depict T47D TRADITIOM samples from POT (pretreatment), awakenings (early progression), and TEPs (late progression) of both treatment arms (TAM and –E2) and their counterpart untreated (UT) samples cultured in parallel for 180 days.

Tumor relapse in patients with ER^+^ breast cancer is thought to emerge either from expansion of preexistent drug-resistant clones without any intermediate dormancy (16, 22, 23) or by *de novo* mechanisms appearing under therapeutic pressure during dormancy (1, 2, 13, 17). To capture and quantify these events at scale, we developed a long-term *in vitro* lineage-tracing method termed TRADITIOM (**TR**acking **A**daptation, **D**ormancy and awaken**i**ng with mul**tiom**ics; Supplementary Fig. S8A). TRADITIOM bypasses many of the common confounding factors that have limited previous studies (i.e., serial cell passaging, small populations, and short-term follow-up; refs. 22, 24, 25) and accounts exclusively for cancer cell-intrinsic mechanisms. The founder population of TRADITIOM contained 100,000 ER^+^ MCF7 cells tagged by 100,000 unique barcodes (Supplementary Fig. S8A). Transduced cells were expanded for 13 days up to 90 million cells (the “POT”; full description of the nomenclature in the Methods section), which now contained barcodes at different frequencies reflecting heterogeneous replicative fitness of individual lineages (Supplementary Fig. S8B and S8C). Extensive barcode profiling shows that randomization does not introduce biases in baseline barcode frequencies (Supplementary Fig. S9), allowing us to create 56 carbon copies (replicates). Seventeen carbon copies were then randomized into long-term estrogen deprivation (−E2), mimicking AI treatment, whereas another 17 were assigned to long-term tamoxifen (TAM) treatment. An additional arm of the study followed 3 replicates of serially passaged untreated (UT) cells to capture *de novo* genetic and nongenetic events occurring under neutral drift (Supplementary Fig. S8A). Both TAM and −E2 triggered a period of putative dormancy after one month of treatment, as shown by live and floating cell counts obtained from intermediate sampling (Fig. 2A; Supplementary Fig. S10A) confirming that endocrine therapies (ET) have a dual cytotoxic and cytostatic impact on ER^+^ breast cancer cells (2, 26). ET-treated cells were maintained under continuous selective pressure in the absence of cell splitting until suspected awakening (early progression, cells that resumed proliferation after dormancy), which is defined by a sudden and exponential change in cell number (Fig. 2A). The first suspected awakening events occurred around day 90 in the TAM arm (TAM η−θ carbon copies) and day 105 in the −E2 arm (AIα carbon copy, Fig. 2A). The remaining carbon copies awakened with no obvious pattern over the course of the following 40 to 60 days (Fig. 2A; Supplementary Fig. S10A). Transient suspension of therapeutic pressure (drug holidays) did not considerably alter these dynamics, suggesting that dormancy is not purely the reflection of therapeutic pressure (Supplementary Fig. S10B–S10F). Of note, dormancy and awakening signatures are not composed solely by cell-cycle–related genes (described later in “Tracking adaption in single lineages” section), indicating that cell-cycle arrest is not the only determinant of the dormant phenotype. A similar random pattern of awakening was observed in a second independent ER^+^ breast cancer cell line model (*p53-mutant L194F* T47D; Fig. 2A). To corroborate these findings, we followed cell proliferation with continuous live imaging over the course of 5 months in additional 12 replicates (TRADITIOM Live, nuclear-GFP tagged, Fig. 2B). All carbon copies entered dormancy within one month of estrogen deprivation (−E2) yet awakened asynchronously, with 2 replicates remaining dormant after 150 days of continuous therapy (Fig. 2B). We found that marginal differences in seeding density were not associated with awakening timing (violin plots, Fig. 2B). Taken together, these data demonstrate that ETs can induce long-term dormancy *in vitro* with unpredictable asynchronous awakening dynamics.

### The Persister Pool Is Induced Stochastically

We performed lineage tracing to chart clonal evolution during dormancy entrance and awakening (early progression). First, we asked if the dormant persister pool emerging after 30 days of ET treatment is generated via selection or induction. Mathematical modeling identified a small fraction of putatively dormant cells in untreated POT (treatment naïve) in agreement with our previous observation (ref. 2; Supplementary Fig. S11A). Although these cells could in principle be selected by ET, their frequency (∼1/10,000) is neither sufficient to explain the size of the persister pool (Fig. 2A) nor the number of lineages present at dormancy (34% and 37%, −E2 and TAM MCF7, respectively, and 18%, −E2 T47D, Supplementary Fig. S11B and S11C), suggesting that most of the dormant pool is induced *de novo* by ETs. Lineage tracing highlighted day 30 as a significant bottleneck in the evolutionary arc with lineage extinction slowing in dormancy (Supplementary Fig. S10A and S11B and S11C). Our data show that within the first 30 days, serially passaged flasks (UT, untreated arm) and ET-treated unperturbed population lose barcodes at a similar rate. We also found that pretreatment barcode frequencies were strongly predictive of lineage survival in all arms (Supplementary Fig. S12A–S12D), suggesting that dormancy entrance is stochastic. To formally test this prediction, we developed TRADITIOM Dormancy (see Methods; Supplementary Fig. S12E), where we triggered therapy-induced dormancy in 96 carbon copies. We then compared simulated data, where all cells have an equal probability of entering dormancy, to observed empirical data (Supplementary Fig. S12E and S12F). These data strongly support a dormancy lottery scenario with no preexistent lineage poised to enter dormancy upon treatment exposure *in vitro*. These conclusions agree with a recent CRISPR-screen (doi.org/10.1101/2022.02.15.480537) conducted in the context of estrogen deprivation which shows that replicates strongly diverge during the first months of estrogen deprivation, leading to stochastic enrichment of gRNA guides.

### Adaptive Trajectories Are Not Driven by Genetic Hits

Considering the unpredictable timeline of awakening and the stochastic dormancy entry (14, 27), we hypothesized that awakening was unlikely the result of selection of a preexistent clone. Nevertheless, progressively acquired genetic hits (i.e., *ESR1* mutations) could still explain awakening. To fully characterize the contribution of genomic changes, we leveraged joint lineage tracing and WGS (Supplementary Fig. S13A). Barcode analysis in MCF7 cells showed that on average each awakened carbon copy contained between 4,000 and 15,000 persisting barcodes (−E2 and TAM, respectively) and 20,000 barcodes in T47D cells (Supplementary Fig. S11B and S11C). Barcode frequencies in the untreated (UT) arm showed that a small set of recurrent high-frequency lineages slightly increased their relative frequency following the rich get richer dynamic (ref. 28; Fig. 2C). On the other hand, we observed clonal sweeps for most of the −E2 and TAM carbon copies (Fig. 2C). These sweeps were driven by carbon copy–specific lineages. Of note, drug holidays resulted in identical dynamics (Supplementary Fig. S10E). Nanopore sequencing demonstrated that in the AIγ carbon copy (two presumptive winners) both barcodes integrated jointly, thus tracking a single lineage.

We next tracked all variants (germline, somatic preexistent, and *de novo)* using Platypus (ref. 29; Supplementary Table S1). Platypus identified a total of 5,963,202 variants (4,332,973 SNVs and 1,630,229 other variants like Indels, see Methods). Subclonal mutations that are subjected to selection (driver or passengers) are expected to change in their frequency within the population. For this reason, we identified those mutations that significantly changed in their variant allele frequency (VAF) across samples. A total of 359,316 variants (6.03%) showed evidence for changing VAF (*P* < 0.01). Most of these changes were explained by copy-number alterations that occurred in the samples (Supplementary Fig. S13B and S13C). These data suggest that most preexisting mutations hitch-hiked to a higher VAF within the awakening lineages, but we cannot exclude that some of these might indeed have occurred *de novo* and could thus have caused the awakening. A subset of 76,523 mutations was absent from at least one POT sample (pretreatment population; i.e., average VAF < 5% across the POTs), but present in one or multiple samples of the UT (untreated), –E2 or TAM arm and we used these to reconstruct phylogenetic relationships of samples (Fig. 2D). All replicates including the UT arm showed genetic divergence, suggesting that acquisition of resistance occurred independently in genetically unrelated lineages. Also, winner barcodes followed independent trajectories in all carbon copies, suggesting that individual lineages do not follow reproducible adaptive paths (Supplementary Fig. S14A–S14C). Previous large-scale genomic studies suggest the existence of over 90 potential drivers for ER^+^ breast cancer (30), we therefore considered the possibility that each carbon copy acquired individual resistance drivers. The analysis of all breast cancer driver genes showed the presence of three *ESR1* mutations (Fig. 2E; Supplementary Fig. S15A). Two of these, p.K268N and p.A64D, are neutral with p.K268N possibly already present in the POTs, whereas the p.H524L mutation might be associated with TAM resistance (31) but emerged exclusively in the TAMβ carbon copy before becoming subclonal in TAMβ TEP (terminal endpoint or late progression, obtained from awakening samples kept in culture 1 more month with the introduction of serial passaging), raising doubts on its link to the awakening lineage (Fig. 2E). None of the other *de novo* SNVs (i.e., *BCL11A*, *BCOR*, and *COL1A1*) was recurrent in more than one sample nor has been linked to treatment failure in breast cancer, leaving the evolution of resistance unexplained in 12 of 13 replicates (Supplementary Table S1). We next looked at copy-number variants and again found no recurrent alternations besides *ESR1* amplification in TAMη, TAMθ, and TAMα-TEP (late progression; Supplementary Fig. S13C). We could not identify recurrent hits in potentially damaging noncoding SNVs (doi.org/10.1101/2022.02.15.480537; Supplementary Fig. S15B) as well. Joint barcode and mutational profiling of an independent model (p53-mutant T47D cells) confirmed that awakening occurs in single lineages and is not driven by preexistent or recurrently acquired genetic events (Fig. 2F–H; Supplementary Fig. S15C). Overall, these data suggest that the independent awakening observed in TRADITIOM carbon copies cannot be traced back to genetic drivers.

### Adaptation Involves Divergent Transcriptional Reprogramming

**Figure 3.**
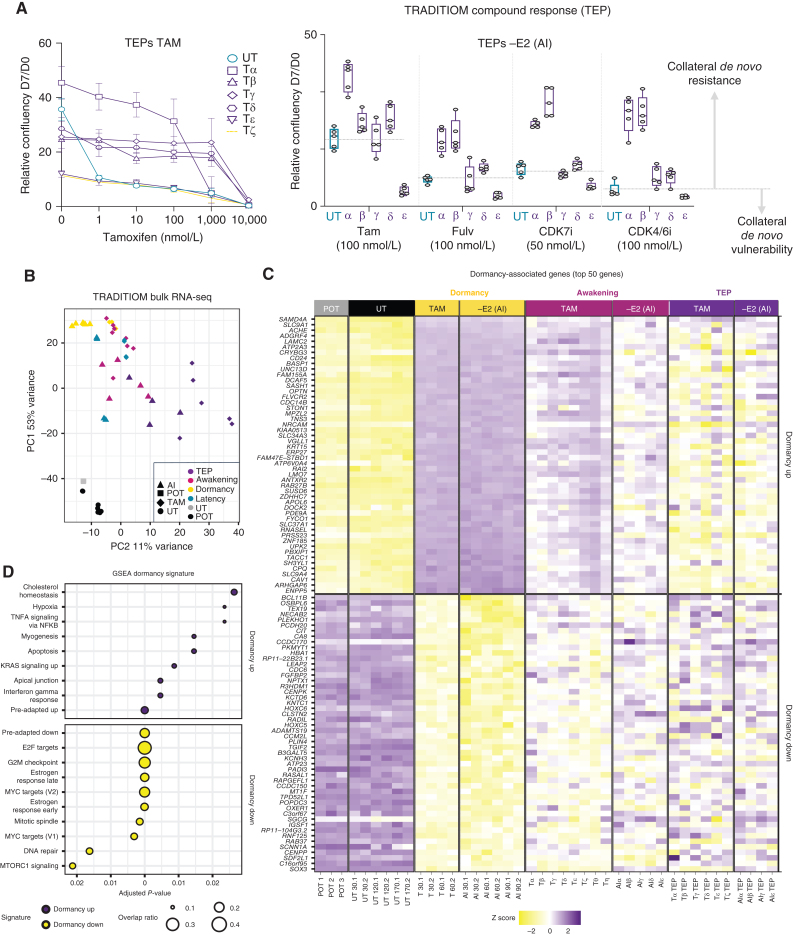
Adaptation is driven by divergent transcriptional reprogramming. **A,** Tamoxifen resistance analysis of the TAM TEPs (late progression) to increasing doses of 4-OHT is depicted in the left. Growth rates of –E2 (AI) TEPs in response to treatment with different drugs: Tamoxifen (Tam, 4-OHT), fulvestrant (Fulv), CDK7 inhibitor (CDK7i), CDK4/6i (palbociclib) are depicted on the right. Representative graphs are shown as normalized confluency fold change upon 7 days of compound treatment (*n* = 3). **B,** Principal component analysis (PCA) of bulk RNA-seq expression data for all MCF7 TRADITIOM samples (POT (pretreatment), latency (time between onset of treatment and dormancy), dormancy, awakening (early progression), and TEP (late progression) of both treatment arms [TAM and –E2(AI)] and their counterpart untreated (UT) samples cultured in parallel for 170 days. **C,** Heat map from bulk RNA-seq data depicting a subset of TRADITIOM MCF7 dormancy signature with top 50 significantly up- and downregulated genes in TAM and –E2 (AI) treated samples during dormancy in comparison with POT (pretreatment). **D,** GSEA for TRADITIOM dormancy signature.

TRADITIOM carbon copies awakened with evident morphologic differences and proliferation rates (Supplementary Fig. S16A–S16C). Unexpectedly, TAM-TEP carbon copies (TAM arm-late progression replicates) also exhibit vastly different drug responses to increasing doses of TAM (Fig. 3A). Divergent collateral drug resistance was even more evident in the −E2 TEPs (estrogen deprivation arm-late progression replicates) and involved carbon copy–specific sensitivities to most second-line treatments, including fulvestrant, CDK4/6i, and CDK7i; Fig. 3A). TEPs also displayed varying growth rates in response to E2 reintroduction with drug holiday and −E2 carbon copies being particularly susceptible to E2 (Supplementary Figs. S10F and S16D). These data demonstrate that awakening involves divergent endpoint phenotypes.

We next asked if these divergent traits correspond to increased transcriptional heterogeneity between different carbon copies, analogously to what we observed in spatially resolved transcriptomes (Fig. 1D). RNA-seq data confirmed the lineage sweep in all carbon copies (Supplementary Fig. S17A). The induced RNA-seq profile of dormant cells appeared to be reproducible and stable over the course of months with consistent downregulation of cell-cycle and metabolic pathways (Fig. 3B–D; Supplementary Tables S4–S7). Awakenings (early progression) and TEPs (late progression) exhibited significant divergence, in line with morphologic and drug-resistance profiles (Fig. 3B and C; Supplementary Figs. S16D and S17B). TEP carbon copies continued to evolve past the awakening phenotype (Fig. 3B and C) in agreement with our cis-regulatory screen, which shows that different from treatment-naïve MCF7, awakened cells’ fitness can be improved (doi.org/10.1101/2022.02.15.480537). On the other hand, six months of neutral drift did not significantly alter the transcriptional profile of the UT arm despite the significant loss of lineages and the associated clonal expansion of four recurrent clones (Figs. 2C and 3B and C; Supplementary Fig. S11B). These data challenge the idea of long-term passaging as a source of transcriptional variability in MCF7, at least for cells maintained in identical culturing conditions (32). Collectively, these data strongly support the notion that ET corners cells in dormancy but awakening propels dormant cells into an unpredictable phenotypic landscape.

### Tracking Adaption in Single Lineages

**Figure 4.**
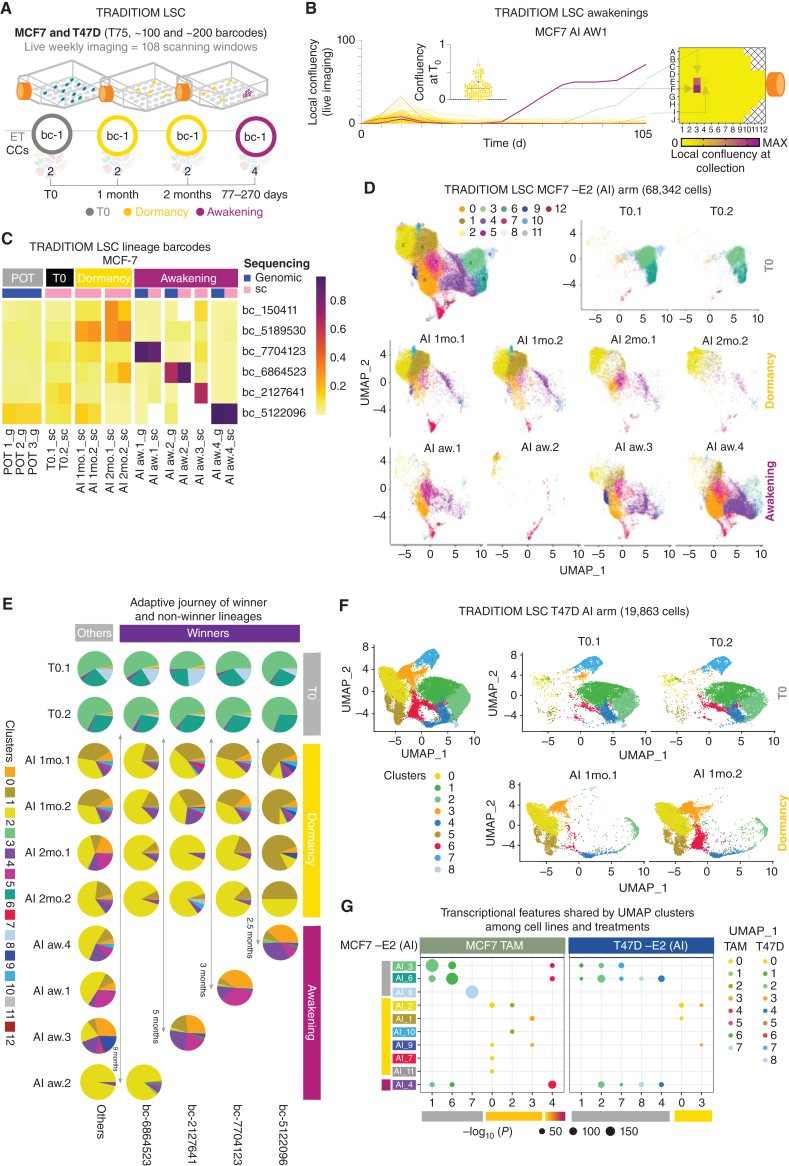
The adaptive journey of individual lineages at single-cell level. **A,** Schematic cartoon of TRADITIOM LSC experimental design. A low-complexity (100 barcodes for MCF7 and 200 barcodes for T47D) barcoded population was seeded in T75 flask format and exposed to ET (TAM and –E2 for MCF7 and –E2 for T47D) in a nonperturbed system (no serial passaging). Cells were collected at the indicated time points (duplicates of T0, 1 month, 2 months, and 4 awakening samples for MCF7 and duplicates of T0 and 1 month for T47D) and analyzed by scRNA-seq. Cells were imaged once a week till awakening with 108 scanning windows covering each flask. **B,** Proliferation dynamics of TRADITIOM LSC for a representative sample [AI (−E2) aw1] determined by weekly imaging along with the local confluency heat map at the time of collection (awakening). Magenta lines follow the growth dynamics of main awakening areas from time zero (T0) to overt expansion that represents the collection point for scRNA-seq analysis. Violin plot shows the confluency of each scanning area at T0 (onset of estrogen deprivation). The T0 confluency of the awakening area is highlighted in purple. **C,** Heat map of winner barcodes’ frequency for TRADITIOM LSC carbon copies for –E2 (AI) arm at the time of awakening, in the POT population (pretreatment), at the start of the experiment (T_0,_ time zero) and at early [1 month (1 mo)] and late dormancy [2 months (2 mo)] stages derived from either genomic barcode sequencing (g) or scRNA-seq (sc). **D,** UMAP projections of MCF7 TRADITIOM LSC –E2 (AI) arm of T0, early [1 month (1 mo)] and late dormancy [2 months (2 mo)] and awakening samples. **E,** Adaptive journey of winner and non-winner lineages (others) of each TRADITIOM LSC –E2 (AI) awakening sample from T0 to awakening (early progression). Pie charts depict the occupancy of miscellaneous UMAP clusters for each lineage. The approximate awakening time of each carbon copy (replicate) is shown with arrows. **F,** UMAP projections of T47D TRADITIOM LSC samples at T0 and dormancy [1 month (1 mo)]. **G,** Dot plot indicates similarity of transcriptional space occupied by MCF7 cells under −E2 treatment (AI) with those under TAM (tamoxifen) and with T47D counterpart under −E2 treatment. Marker genes for clusters in T0, dormancy, transition, and awakening from MCF7 TAM and T47D −E2 UMAPs were checked for enrichment in cluster marker genes from MCF7 −E2 (AI) UMAP. Size of the dot represents the significance of enrichment in context to –log_10_(*P* value; correction method: FDR, *P* ≤ 0.05). The color of the dots corresponds to the respective color of clusters at T0, dormancy, and awakening annotated by colored bars next to cluster names on the *x* and *y* axes (T0: gray, dormancy: yellow, awakening: magenta).

Both spatial transcriptomics of patients and lineage tracing suggest that awakening clones coexist with dormant persister cells and/or that awakened cells can spontaneously reconvert to a dormant cell state (Figs. 1D and 2B, C, and F). Moreover, our data cannot formally exclude that different winner lineages emerge from a common phenotypic clone which was tagged by different barcodes at the start of the experiment. To tackle these concerns, we implemented TRADITIOM Live Single-Cell (TRADITIOM LSC), where we combined live imaging and lineage tracing with single-cell RNA-seq (Fig. 4A). We reduced the complexity of TRADITIOM LSC by subsampling 100 lineages from the original TRADITIOM to match the sampling capacity of 10× chromium (targeting 10,000 cells from each timepoint; Fig. 4A). We optimized a protocol allowing for joint barcode detection and high depth phenotyping that enabled profiling on average more than 3,500 genes and barcode call in ∼95% of scRNA-seq data sets (Supplementary Fig. S18A–S18E). None of the original TRADITIOM winners was featured in TRADITIOM LSC, highlighting the stochastic nature of adaptation. TRADITIOM LSC carbon copies awakened unpredictably between 77 and 154 days, with the final carbon copy collected at day 270 (Fig. 4B; Supplementary Fig. S19A and S19B). Barcode dynamics closely matched the one observed in TRADITIOM (Supplementary Fig. S11B), with ∼22% to 49% of lineages still present at awakening (early progression) despite a full clonal sweep (Fig. 4C; Supplementary Fig. S20A and S20B). We also confirmed that adaptation to −E2 (AI) and TAM follows different routes with AI driving the clonal expansion of unique lineages in each carbon copies, whereas TAM has weaker sweeps, partial barcode overlaps, and a higher proportion of cells still in dormancy at the time of awakening (Supplementary Fig. S21A–S21C).

Next, we created a transcriptional atlas of adapting cells focusing our attention on the −E2 (AI) arm (68,342 single cells) because AI currently represents the standard of care for most patients and the previous lineage-tracing experiment did not include AI treatment (24, 33). Clustering analyses identified 13 cell states after stringent batch correction (Fig. 4D). Clusters were significantly associated with distinct cell states (i.e., cluster 3–6–8 enriched exclusively in pretreatment samples, cluster 1–2 dominating dormant samples). A significant upregulation of dormant features emerged and persisted after 30 days of treatment with most cells entering a potential G_1_ arrest (Fig. 4D and E; and Supplementary Fig. S22A–S22C). Cells from awakening time points, on the other hand, occupy a rather heterogenous set of cell states (Fig. 4D). Despite sharing active cell-cycle features, pretreatment and awakening cells exhibited dramatic differences in line with our patient-derived spatial transcriptomics analyses (Fig. 1D).

Joint barcode-phenotyping allowed us to track adaptation with single-cell resolution and demonstrate that awakening and dormant lineages coexist at the time of awakening (Fig. 4D). Additionally, awakened clones potentially gain plasticity because they can reacquire dormant features (Fig. 4E). These data also explain the sharp lineage extinction observed in the original TRADITIOM TEPs, which can be ascribed to the loss of dormant persisters during serial replating, highlighting the crucial advantage of our experimental setting in preserving population heterogeneity up to cell awakening (Supplementary Fig. S11B and S11C). Our data do not support the notion that winner lineages belong to a preexistent epigenetic clone because they are indistinguishable from non-winner lineages in treatment-naïve samples (Fig. 4E). Finally, all lineages enter dormancy with comparable dynamics, suggesting that awakening happens as the result of a stochastic transition within the dormant pool (Fig. 4E; Supplementary Fig. S23A and S23B). Intriguingly, T47D shows a stronger association between lineages and the choice of dormant cell state (cluster 0 vs. cluster 3; Supplementary Fig. S23C). Of note, regressing out cell-cycle genes from the analysis confirmed our findings, demonstrating that the dormant cell state is not simply a reflection of the cell-cycle arrest (Supplementary Fig. S24A and S24B). Dormancy features appear to be conserved across treatments (−E2 and TAM) and cell lines (T47D and MCF7; Fig. 4F–4G). In conclusion, our data strongly suggest that adaptation to therapy is driven by acquired nongenetic cell state transitions within single lineages.

### Adaptation Involves a Series of Failed Awakenings

**Figure 5.**
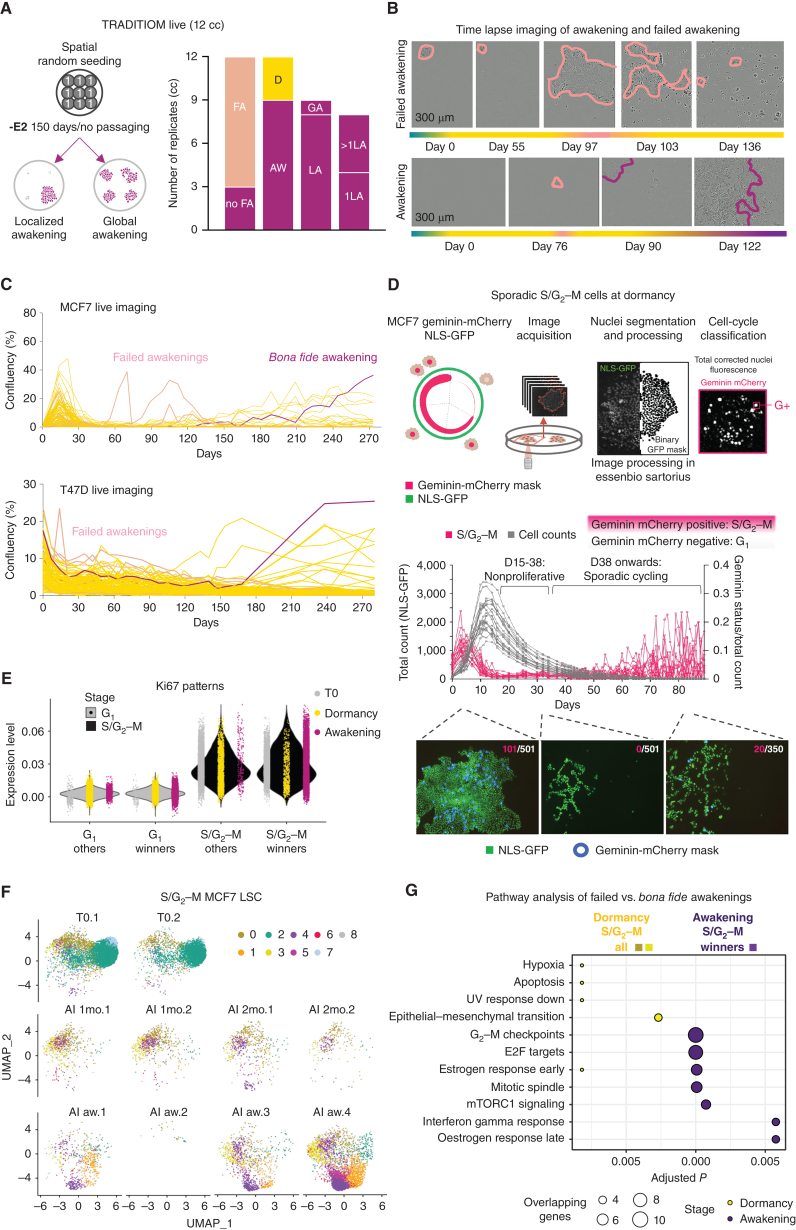
Failed awakenings. **A,** TRADITIOM Live awakening topography analysis depicting the awakening dynamics. Twelve carbon copies (cc; replicates) were seeded in a 48-well plate and imaged two times a week for 150 days using IncuCyte Zoom (9 scanning windows per well) to monitor awakening dynamics. Awakening was defined as wells reaching a confluency of 50% (FA: failed awakening; D: dormant; AW: awakening; GA: global awakening; LA: localized awakening; 1LA: 1 localized awakening; >1LA: more than 1 localized awakening). **B,** IncuCyte time-lapse images from a failed and a bona fide awakening are shown as an example. **C,** Proliferation dynamics of a representative MCF7 [AI (−E2) awakening 2] and T47D TRADITIOM LSC sample determined by weekly IncuCyte time-lapse imaging until awakening. Pink lines indicate failed awakenings. Magenta lines follow the growth dynamics of main awakening areas. **D,** MCF7 Geminin-mCherry NLS-GFP cells were treated with estrogen deprivation (−E2) for 3 months to establish a detailed understanding of long-term dormancy–awakening dynamics. Image sets were analyzed using Essenbio Sartorius software from daily imaging. 35% of replicates (*n* = 60) had dormant persister cells/small colonies until day 88. The proportion of Geminin-mCherry-positive (S/G_2_–M; pink lines) is indicated normalized to total count which was quantified by NLS-GFP (gray lines). **E,** Distribution of Ki-67 expression levels in winner and non-winner (others) lineages associated with either G_1_ or S/G_2_–M states across T0, dormancy, and awakening samples of MCF7 TRADITIOM LSC cell-cycle regressed data set. **F,** Cell-cycle regressed UMAPs of the subset of cells in S/G_2_–M state of TRADITIOM MCF7 LSC data set. **G,** Pathway enrichment analysis comparing failed awakenings (S/G_2_–M cells in dormancy: cluster 0, 3) to bona fide awakenings (S/G_2_–M cells of awakening winner lineages: cluster 4).

Barcode analyses suggest that one lineage can sweep through the entire carbon copy; however, these data cannot distinguish between simultaneous multiple awakenings of the same lineage (global), consecutive localized- or a singular local event. TRADITIOM Live images (Fig. 2B) showed that in 8 of 9 awakened −E2 carbon copies (replicates), the final clonal sweep emerged in a single area of the plate (Fig. 5A; Supplementary Fig. S25A). As expected, persister colonies across the plates showed dormant features with almost complete absence of cell proliferation. Interestingly, most carbon copies, including all dormant ones, contained clones transiently reentering the cell cycle before partial extinction and generation of new dormant persisters, a process we labeled failed awakening (Fig. 5B; Supplementary Fig. S25A). Failed awakenings were also documented in TRADITIOM LSC for both MCF7 and T47D models (Fig. 5C). These data suggest that lineages might attempt awakening several times, but plasticity alone is insufficient to support the evolution of full resistance.

To validate the existence of failed awakening, we developed a cell-cycle reporter cell line that can be tracked by live imaging (Fig. 5D). Long-term live imaging of adapting cells confirms that dormancy state corresponds to a prolonged G_1_ arrest (low Geminin). More importantly, we could capture sporadic cycling in a random subset of cells that eventually regressed to cell-cycle arrest (often followed by partial extinction). These data suggest that the presence of S–G_2_–M cells during dormancy does not considerably contribute to an increase in total cell number and represents failed awakenings.

To gain more insights on this phenotype, we retrospectively classified single cells from TRADITIOM LSC based on their cell-cycle state and compared S/G_2_–M cells from the untreated time point (T0) to S/G_2_–M cells from dormant (failed awakenings) and awakening timepoints (*bona fide* awakenings; Supplementary Fig. S25B). First, we validated that our classification accurately captured the cell-cycle activity (Fig. 5E). Then we clustered cell-cycle regressed cells based on their transcriptional profile and confirmed the presence of cycling cells during dormancy (Fig. 5F). This analysis also showed that cycling cells acquire different phenotypes while adapting to ET as shown by different occupancy of transcriptional states of cycling cells in dormant samples compared with those in treatment-naïve and fully awakened conditions (Fig. 5F). Finally, we compared the transcriptional landscape of failed awakenings (dormant S/G_2_–M scRNA-seq from all barcodes) to *bona fide* awakening (awakening S/G_2_–M scRNA-seq from winner barcodes). Cells captured in a failed awakening cell state were enriched for apoptotic and EMT pathways, whereas *bona fide* awakening involves clearing the cell-cycle G_2_–M checkpoint and reactivating estrogen signaling despite the lack of available ligand (ref. 34; Fig. 5G). Collectively, these data strengthen the hypothesis that nongenetic cell state transitions are required for *bona fide* awakening, and reentering the cell cycle is necessary but not sufficient to acquire a *bona fide* awakening phenotype.

### Adaptation Is Characterized by Epigenetic Reprogramming and Erosion

**Figure 6.**
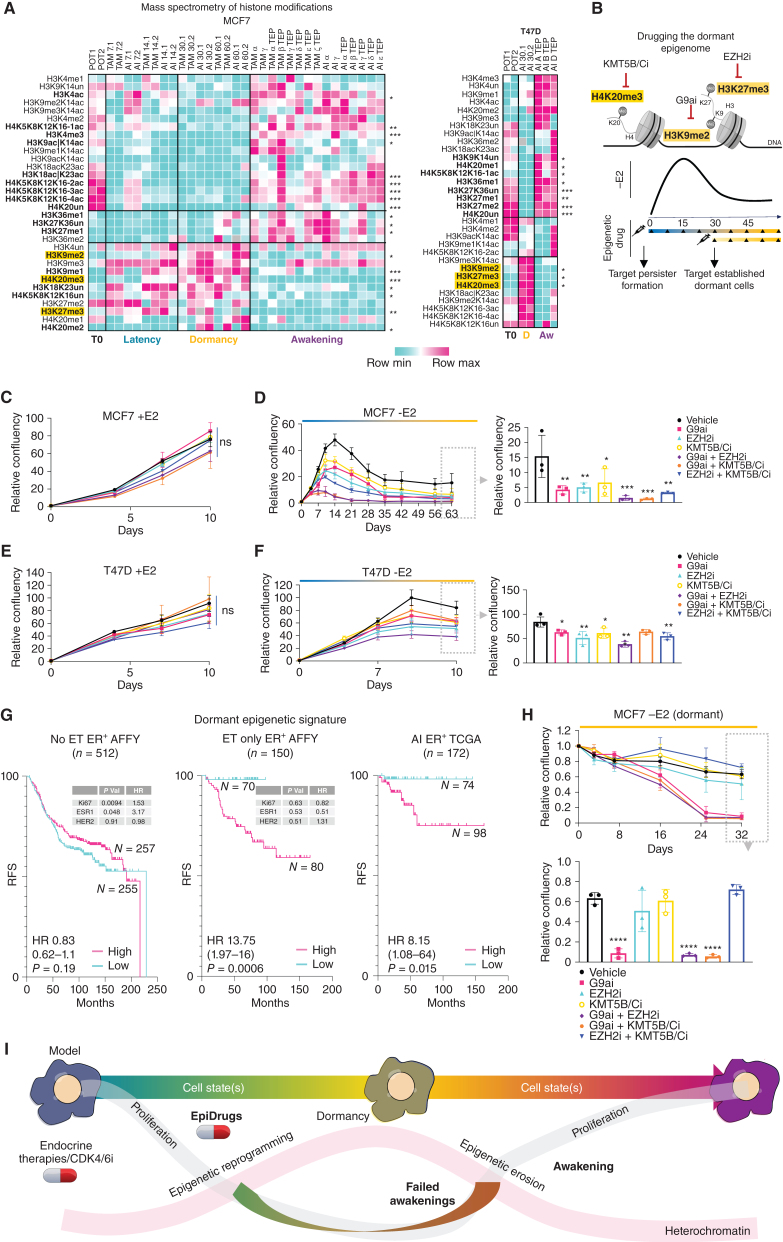
Targeting the dormant epigenome. **A,** Clustered heat maps of histone posttranslational modifications of super-SILAC mass spectrometry for TRADITIOM MCF7 and T47D samples [time zero (T0), latency (time between treatment onset and dormancy entry), dormancy, awakening (early progression), and TEPs (late progression)]. Significantly enriched (dormancy 30 days vs. TEPs, two-tailed *t* test: *, *P* < 0.01; **, *P* < 0.001; ***, *P* < 0.0001) modifications are depicted in bold, and the ones found to be associated with dormancy are highlighted in yellow. **B,** Schematic representation of small-molecule inhibitor experiments. Inhibitors against G9a (H3K9me2), EZH2 (H3K27me3), and KMT5B/C (H4K20me3) were used either alone or in combination. Start time of the inhibition was either at the beginning of estrogen deprivation to target persister pool generation or at 30 days of estrogen deprivation (dormancy) to target established dormant cells. **C,** Proliferation dynamics of MCF7 cells in E2-supplemented conditions (+E2) after treatment with inhibitors against EHMT2, EZH2, KMT5B/C, dual combinations of each and vehicle. **D,** Proliferation dynamics of MCF7 cells in estrogen-deprived conditions (−E2) after treatment with inhibitors against G9a, EZH2, KMT5B/C, dual combinations of each and vehicle. Proliferation dynamics of T47D cells in E2-supplemented (+E2; **E**) and deprived (−E2) conditions (**F**) after treatment with inhibitors against G9a, EZH2, KMT5B/C, dual combinations of each and vehicle (one-way ANOVA with Dunnett correction: *, *P* < 0.05; **, *P* < 0.01; ***, P < 0.001; ****, P < 0.0001). Error bars represent standard deviation (*n* = 3). **G,** Relapse-free survival (RFS) curves for ER^+^ breast cancer patients stratified based on the expression of the epigenetic dormancy signature (high vs. low *EHMT2/EZH2/KMT5C* expression). Left: no adjuvant treatment; middle: adjuvant endocrine therapy (TAM/AI); right: AI adjuvant treatment. Multivariate analysis for clinically relevant prognostic biomarkers is shown in the onset table. **H,** Proliferation dynamics of MCF7 dormant cells (pretreated for 30 days with –E2) after treatment with inhibitors against G9a, EZH2, KMT5B/C, dual combinations of each and vehicle (one-way ANOVA with Dunnett correction: *, *P* < 0.05; **, *P* < 0.01; ***, *P* < 0.001; ****, *P* < 0.0001). Error bars represent standard deviation (*n* = 3). **I,** Model: endocrine therapy-induced dormancy is characterized by a consistent epigenetic reprogramming involving a global increase in histone repressive marks (H3K9me2, H3K27me3, and H4K20me3). The dormant epigenome is unstable and through a progressive loss of the histone repressive marks (erosion), cells resume proliferation in a process that mimics patient relapse (awakening). Epidrugs (G9a/EZH2/KMT5B/C inhibitors) can interfere with epigenetic reprogramming and block the formation of persister dormant clones. During adaptation, dormant cells engage in sporadic cycling (failed awakening) while under therapeutic stress possibly forcing cells into a subsequent round of epigenetic reprogramming that could also be antagonized with epidrugs.

Both the *in vivo* and *in vitro* arms of the study strongly indicate that adaptation to ET involves heritable nongenetic transitions leading cells into a dormant state before drug resistance emerges within one lineage. In the absence of heritable genetic drivers, these transitions might be driven and stabilized by epigenetic change. We recently reported that ER^+^ breast cancer evolution includes epigenetic reprogramming at enhancer elements, as shown by the differential distribution of Histone 3 Lysine 27 acetylation marks (H3K27ac) across the genome. We hypothesized that posttranslational histone modifications (PTM) could be an ideal candidate for inducible and heritable cell state transitions during adaptation. To test this, we used super-SILAC mass spectrometry to quantify changes in histone modifications during adaptation in an unbiased manner. Carbon copies from both models (MCF7 and T47D) show reproducible epigenetic changes during adaptation (Fig. 6A; Supplementary Fig. S26A and Supplementary Table S8). ET triggered the accumulation of H3K9me2, H3K27me3, and H4K20me3 heterochromatin marks and reduction in H3K4me3 and H3K9/14ac during dormancy entrance. The heterochromatin state is partially reversed at awakening (early progression) and TEPs (terminal endpoints, late progression) but their epigenome remained distinct from the baseline (Fig. 6A; Supplementary Fig. S26A). These observed epigenetic changes were validated with independent assays (Supplementary Fig. S26B and S26C).

These data raised the question if the dormancy-associated heterochromatin state is essential for dormancy entrance and maintenance or is a passenger event. To discriminate between these two possibilities, we treated MCF7 with small-molecule inhibitors targeting EZH2 (catalyzing H3K27me3), G9a (EHMT2, catalyzing H3K9me2), and KMT5B/C (aka SUV420H1/2, catalyzing H4K20me2-3) in two contexts: first, in combination with estrogen deprivation (−E2) to study their impact on dormant persister formation, and second by adding the inhibitor after 30 days of pretreatment to evaluate the impact on established dormant cells (Fig. 6B). All inhibitors exhibited activity against their respective targets (Supplementary Fig. S26D–S26F). Blocking the activity of EZH2, G9a (EHMT2), or KMT5B/C did not affect the cell proliferation dynamic of treatment-naïve cells (Fig. 6C). However, targeting heterochromatin writers severely hampered the formation of therapy-induced persister dormant cells (Fig. 6D). Similar results were obtained in ER^+^ T47D cells (Fig. 6E and F). These data prompted us to investigate if tumors characterized by low expression of heterochromatin writers would have limited adaptive potential and therefore increased susceptibility to single-agent ET. To test these, we stratified ET-treated ER^+^ breast cancer patients for a *G9a-EZH2-KMT5C* signature and observed that patients with low expression have a significantly lower risk of relapse over the course of 15 to 20 years as compared with high expressors (Fig. 6G; and Supplementary Fig. S27A and S27B). Of note, the same signature did not stratify systematically untreated ER^+^ breast cancer nor ER-negative patients strongly supporting our hypothesis that epigenetic adaptation is therapy induced (Fig. 6G; Supplementary Fig. S27C and S27D). We next targeted heterochromatin writers in dormant cells and observed a progressive eradicating effect for G9a (EHMT2) inhibition (Fig. 6H), despite bulk and spatial transcriptomics data indicating a significantly lower expression of the target during dormancy (Supplementary Fig. S28A–S28C). This prompted us to investigate the expression pattern of heterochromatin writers at the single-cell resolution. Interestingly, TRADITIOM LSC revealed the expression of *G9a (EHMT2)* during dormancy spikes in S–G_2_–M cells (putative failed awakening, Supplementary Fig. S28D). Our data would then suggest that G9a is important for the adaptive potential of failed awakenings. Collectively, these observations indicate that targeting epigenetic reprogramming can interfere with the evolutionary processes required to adapt to ETs.

## DISCUSSION

Mounting evidence suggests that nongenetic heritable cell state transitions play a central role in cancer evolution (12, 35, 36), but how they contribute to ER^+^ breast cancer adaptation to ETs is still elusive. Adjuvant ETs target microdisseminated cancer cells in different organs, which remain undetectable until clinical progression. This has limited our ability to study the impact of long-term ET *in vivo*. Our work focusing on a rare set of patients exposed to long-term ET in the absence of surgery allowed us for the first time to study adaptation *in situ*. Multiregion spatial profiling coupled with WGS suggests that awakening from therapy-induced dormancy occurs asynchronously and does not involve recurrent genomic changes. The size of our patient cohort, however, does not preclude the existence of some unknown genetic mechanism.

These findings were recapitulated and modeled *in vitro* in an evolutionary study mapping cell state transitions at a single lineage resolution over the course of months*.* Previous lineage-tracing studies did not model AI treatment, used smaller populations, shorter time frames, and were heavily confounded by cell passaging, which could have resulted in a rapid loss of dormant persister cells possibly leading to biased lineage representation (24, 37). Our unperturbed experimental setup has allowed us to track at-scale cell-intrinsic dynamics of dormancy and awakening and to highlight some unexpected principles controlling these processes. First, we could model the cytotoxic–cytostatic impact of ET (2) to show that dormant persister cells emerge with a marked epigenetic reprogramming occurring within a fraction of cancer cells. Our data suggest that lineages contribute stochastically to the minimal residual disease. Second, dormant cells attempt sporadically to reenter the cell-cycle, but these efforts often end up in cell death or a return to a dormant cell state (failed awakening). *Bona fide* awakening is a distinct phenotype from failed awakening, demonstrating that reactivation of the cell-cycle is not sufficient for the evolution of drug resistance. Taken together, lineage tracing and failed awakening also highlight the role of nongenetic mechanism in adaption to ET, which is distinct from pure transcriptional plasticity. The discovery that the formation of therapy-induced dormant persisters and their awakening requires epigenetic reprogramming has potential clinical implications. Targeting genetically fueled evolutionary process (mutagenesis) has proven to be difficult, with standard chemotherapy possibly transiently increasing genetic heterogeneity (38). On the other hand, our data show that long-term ETs bottleneck a random subset of lineages into a dormant persister cell state with reduced epigenetic heterogeneity. Although our matched genomic analysis did not identify any explicit convergent genetic mechanisms, we observed reproducible and functional epigenetic changes during adaptation. More specifically, we found that therapy-induced dormancy is characterized by a global increase in heterochromatin-associated modifications (H3K9me2, H3K27me3, and H4K20me3). Interestingly, we could successfully target this dependency on heterochromatin reprogramming to reduce the formation of dormant persisters. Intriguingly, targeting G9a (EHMT2) in fully dormant cells was effective with delayed response, despite an apparent strong downregulation of its target. Single-cell profiling revealed, however, that a subset of dormant cells sporadically reenters the cell-cycle (failed awakenings) and reexpress *EHMT2*. It is tempting to speculate that cells undergoing failed awakening require G9a to attempt reentering a dormant cell state and further persist within the population. The progressive eradication of dormant cells with G9a inhibition would fit with the cumulative increase in failed awakening observed in our cell-cycle reporter model (Fig. 5D). We hypothesize that an erosion process might be required to resume cell-cycle dynamics despite the continuous presence of therapy due to the lack of EHMT2, EZH2, and KMT5C in dormancy. Indeed, awakening lineages are characterized by an almost complete epigenetic reversal. We also find intriguing the specific increase in H3K36me1 and H3K27me1 in awakened lineages, two understudied histone marks thought to be involved in transcriptional memory and fidelity (39, 40). Future long-term longitudinal studies mapping heterochromatin changes and their association with chromatin 3D structure might shed additional light on the adaptive process (41). Targeting dormant cells could have a broad clinical application, considering that the potential addition of CDK4/6 inhibitors in standard-of-care ET might extend the frequency of therapy-induced dormancy. Additionally, dormant cells carry specific but transient vulnerabilities (doi.org/10.1101/2022.02.15.480537), leaving a largely unexplored space for drug discovery (23). Notably, epigenetic modifiers-based signatures might be useful to stratify patients with ER^+^ breast cancer who would benefit from combinatorial epigenetic therapy. On the other hand, our data suggest that some tumors have lower adaptive potential (i.e., cannot transition to a dormant cell state) and might be resolved by ET alone. Lastly, we show that awakening lineages coexist with dormant ones, which maintain an intrinsic potential for additional awakenings. Considering the divergent phenotypes generated within our experiment, one prediction would be that the subsequent awakenings would generate extensive phenotypical heterogeneity with implications for second-line treatment. This scenario fits with clinical observations where consecutive lines of treatment have progressively shorter responses.

We propose that in treatment-naïve patients, exponentially growing cancer populations can generate sufficient genetic heterogeneity to fuel Darwinian genetics (cancer drivers; refs. 21, 30, 42). After surgery, ETs and CDK4/6i induce a widespread epigenetic cell state transition creating a microdisseminated dormant pool. Nevertheless, dormancy is inherently unstable, and cells can acquire transient states (i.e., failed awakenings) before drug resistance becomes heritably selected at awakening (Fig. 6I). Taken together, our data strongly support an effort toward targeting dormant persister cells (doi.org/10.1101/2022.02.15.480537). Current strategies have focused on combinatorial treatments with limited success (43). Our data would offer an alternative explanation, where the first signs of metastatic progression act as a whistle-blower for additional sequential awakening events within the residual dormant persister cells. We argue that this reflects the difficult task of drugging a moving target and advocates for sequential intervention designed to minimize transcriptional heterogeneity via AI-CDK4/6i followed by targeted approaches tailored to dormant persisters. Although further studies incorporating *in vivo* and *ex vivo* models will help to elucidate the contribution of tumor microenvironment in the fine-tuning of dormancy dynamics, we believe that this study provides a significant leap forward and advances the understanding of therapy-induced dormancy laying the groundwork for future research in this area.

## METHODS

### Cell Culture

Breast cancer cell lines MCF7 and T47D were kindly provided by Philippa Darbre. Cells were cultured in Dulbecco's Modified Eagle Medium (DMEM) supplemented with 10% FCS, 100 units/mL penicillin, 50 μg/mL streptomycin sulfate, 2.5 mmol/L L-glutamine (1% (v/v) PSG, Sigma), and 10^−8^ mol/L 17-ß-estradiol (E2, Sigma-Aldrich) and were kept at 37°C with 5% CO_2_ in a humidified atmosphere with a subculturing ratio of 1:3 three times or twice a week for MCF7 and T47D, respectively. Cells were routinely tested for *Mycoplasma* contamination.

### Cell Culture in Hyperflasks

MCF7 cells were grown without passaging in High Yield PERformance Flasks (HYPERflask) cell culture vessel (Corning, CLS10034) and maintained either in 100 nmol/L 4-Hydroxytamoxifen (TAM, Sigma-Aldrich, H7904) or kept in phenol red-free DMEM (Gibco, 11880028) supplemented with 10% charcoal-stripped FCS (−E2, Estrogen deprivation). Similarly, T47D cells were kept under estrogen deprivation in HYPERflasks. The medium was changed weekly, and cells were monitored twice a week for apparent growth changes and harvested upon awakening (resumption of cell proliferation evaluated by visual inspection using EVOS cell imaging system, Thermo Fisher Scientific). The untreated arm of TRADITIOM was maintained in E2-supplemented media and underwent serial passaging.

### Floating Cell Harvesting

Floating (dead) cells were collected from media at each collection point from the large volume of media (550 mL) of the HYPERflask culture system. MCF7 is an adherent cell line, with cells that detach from the flask surface upon death. By centrifuging the culture media at 1,200 rpm for 5 minutes in Corning 250-mL centrifuge tubes (Corning), we collected cells that had died within the week. Collected cells were counted with a hemocytometer and trypan blue to measure cell viability.

### WGS of Patient Samples

This study was approved by the Institutional Review Boards (Imperial College London and Istituto Nazionale Tumori). Each subject gave written, informed consent prior to enrolment, and the study was conducted in accordance with recognized ethical guidelines (e.g., Declaration of Helsinki, CIOMS, Belmont Report, U.S. Common Rule).

Extraction of DNA from fresh frozen (Buffy Coat and Drug-Resistant Tumor) samples was carried out using a DNeasy Blood and Tissue kit (Qiagen, 69506). Extraction from FFPE samples was conducted using GeneRead DNA FFPE Kit (Qiagen, 180134). Quality and quantity of DNA were determined using the TapeStation 2200 System (Agilent) with the Genomic DNA ScreenTape Analysis (5365). To improve the proportion of DNA fragments at the optimal length for library preparation, samples were sonicated for 10 cycles using the Bioruptor Pico Sonication Device (Diagenode). FFPE DNA samples were treated with the NEBNext FFPE DNA-repair Mix (NEB, M6630L). DNA libraries for Illumina sequencing were prepared with the NEBNext Ultra 2 DNA Library Kit for Illumina (NEB, E7645L) using 200 ng of DNA and custom-made unique dual indices (8 bp), a kind gift from Dr. Paolo Piazza (British Research Council Genomics Facility). DNA libraries were quantified using the TapeStation 2200 System with the High-Sensitivity D1000 ScreenTape Analysis (Agilent, 5584). Samples were pooled based on the type of the original material; FFPE, Fresh Frozen. Normal DNA was pooled at a 1:3 ratio to tumor material. Pooled DNA libraries were sequenced with NovaSeq using the S2 50-bp paired-end flow cell chemistry (output 333–417 Gb).

Clinical information: Patient 1: Metastases—fresh frozen material. Patient 2: Normal—buffy coat—fresh frozen, untreated diagnostic biopsy—FFPE, drug-resistant tumor—fresh frozen. Patient 3: Normal—buffy coat—fresh frozen, untreated diagnostic biopsy—FFPE, drug-resistant tumor—fresh frozen. Patient 4: Normal—buffy coat—fresh frozen, untreated diagnostic biopsy—FFPE, drug-resistant tumor—FFPE. Patient 5: Normal—buffy coat—fresh frozen, untreated diagnostic biopsy—FFPE, drug-resistant tumor—fresh frozen.

### WGS Data Analysis for Patient Samples

Raw reads were trimmed for adapters using skewer (sequence: ACGCTCTTCCGATCT; trimming mode: head; length: 35; stringency: −*r* = 0.1, −*d* = 0.03; quality: 10; ref. 44). The trimmed reads were mapped to GRCh38 with BWA mem (v-0.7.15; ref. 45). This was followed by sorting the alignment maps, marking duplicates and validating with Picard (v-2.20.6). GATK (v-4.1.3.0) mutect2 best practices pipeline was then used for somatic variant calling (–af-of-alleles-not-in-resource 0.0000025). The calls from noncanonical chromosomes and those that do not have PASS as filter criteria were removed. The variants with allele depth greater than 20 and at least 6 read pairs in the F1R2 and F2R1 configurations supporting REF and ALT alleles were used for further analysis. Filtered calls were then annotated with variant effect predictor (VEP). Dot plots were prepared for logical pairs of patient samples to represent VAF in genes with variants within each sample. Gray dots represent all filtered variants. Variants with VAF ≥0.1, FATHMM >0.6, and VEP consequence MODERATE or HIGH were color coded based on the sample in which they are present [diagnosis (teal), progression (magenta)] and dark gray if identified in both samples. Variants in Intogen breast cancer driver genes are labeled according to the same color code and highlighted if they are among the resistance drivers (comprehensive ET resistance driver gene list compiled based on Bertucci et al., ref. 14). Similar plots are created for variants that are identified in Intogen driver genes other than the breast cancer drivers.

### Targeted Sequencing of Late Relapse Patients

DNA was extracted from 10 μm FFPE slices using the Qiagen GeneRead DNA FFPE extraction kit (Qiagen, cat no. 180134) following the manufacturer's instructions. Briefly, paraffin was removed from the samples, cells were lysed, and DNA was treated with the Uracil N Glycosylase enzyme. DNA was then purified using a column-based method. The quantity and quality of DNA between 100 and 50,000 bp was assessed using an Agilent Tapestation 2200 instrument using the Genomic DNA screenTape and reagents (Agilent, cat no. 5067-5365 and 5067-5366). Samples were sonicated with Covaris E220 to reach an average fragment size of 250 bp and sonication efficiency was assessed using the Tapestation 2200 instrument with the Genomic DNA screenTape and reagents. A threshold of 60% of fragments between 100 and 500 bp was set to ensure efficient library preparation. Samples that did not pass this threshold were sonicated further and reassessed. Samples underwent DNA-repair treatment with the NEBNext FFPE DNA-Repair Mix (NEB, cat no. M6630) following the manufacturer's instructions. Briefly, the repair mix and provided buffer were added to DNA and incubated at 20°C for 15 minutes. Library preparation was carried out using the NEBNext Ultra II DNA Library Kit (E7645L) following the manufacturer's instructions. Post-library DNA concentrations were assessed using the Agilent Tapestation 2200 with the High Sensitivity D1000 screenTape and reagents as previously described. Any contamination with persisting adapters was removed through size selection using SPRI size-selection beads. The libraries from patients were pooled and captured with the custom panel (18) produced by Twist Biosciences using the Twist Custom Capture Panel Protocol. Pools of libraries were dried in a ThermoFischer Scientific SPD120 SpeedVac Vacuum Concentrator until less than 3 μL volume was remaining. Capture probes were mixed with the hybridization mix and heated at 95°C for two minutes. Dried library pools were mixed with provided blockers and heated at 95°C for five minutes. After both reactions had cooled to room temperature they were mixed and left to hybridize overnight at 70°C. The next day, the DNA and probe mix was mixed with streptavidin beads to enrich capture probe-bound DNA. The pure capture probes and bead-bound DNA complex underwent a PCR amplification using the KAPA HiFi Hot Start PCR ReadyMix Kit (KAPA Biosystems, cat no. KK2601) following the manufacturer's instructions. The minimal number of PCR cycles possible was used to reduce the introduction of PCR duplicates that could significantly affect sequencing quality. Post-captured quality of DNA was assessed and quantified using both the Qubit and Tapestation. Captured pools were sequenced by Novogene (Cambridge, UK) using the NovaSeq6000 platform (Illumina; paired-end 150 bp).

Raw reads were trimmed for adapters and quality (Phred quality ≥ 30) with trim_galore(v-0.6.4_dev). After confirming the quality of processed reads with FastQC (version v-0.11.9), they were mapped to the human reference genome (hg38) using BWA mem (v- 0.7.17-r1188) with default settings. The alignment maps were then parsed to convert into binary maps marking PCR duplicates, sorting, and indexing using sambamba (v-0.7.1). Depth of coverage was then assessed using the gatk DepthOfCoverage function considering the list of targeted regions that were sequenced. After adding read groups with picard (v-2.27.5), further postprocessing (base quality recalibration, CollectSequencingArtifactMetrics) and variant identification were performed using the Genome Analysis Toolkit (GATK; v- v4.3.0.0; refs. 46, 47) best practices. Somatic variant calling was performed on each sample individually using Mutect2 with a GATK-provided panel of normals (1000g_pon.hg38.vcf.gz) while also collecting F1R2 metrics. A threshold of 0.001 was used for population allele frequency assigned to alleles that are not found in germline resource and read filter for mates on the same contig or no mapped mates was disabled. Reported variant calls were then filtered based on the read orientation model.

Further filtering of variants was performed to keep those with PASS or germline annotations from mutect2, followed by the sum of allele depth for reference and alternate supported by ≥20 reads, sum of F1R2 and F2R1 for alternate ≥4 and allele frequency ≥0.1. These variants were then analyzed with VEP (v-105.0) and filtered for MODERATE or HIGH consequence. DN/DS ratios were then analyzed with the dndscv package in R, and significance levels were reported based on the global q-value of the neutrality test at the gene level (qglobal_cv ≤0.1) as well as confidence intervals for the dN/dS ratios per gene (CI for missense and truncating mutations that do not span through the value of 1). Heat maps were plotted for Intogen breast cancer driver genes and ET resistance drivers reported by Bertucci and colleagues (14).

### GeoMx

Spatially resolved transcriptomic analyses were carried out on the GeoMx platform (NanoString Technologies) with the Human Whole Transcriptome Atlas; NanoString Technologies), following the manufacturer's recommendations. *In situ* hybridization was performed on 4-μm FFPE tissue slides. After deparaffinization, rehydration, and washing with PBS (Sigma-Aldrich P-5368), slides were incubated for 20 minutes in 1 × Tris-EDTA pH 9.0 buffer (Invitrogen, Life Technologies, CA 00-4956-58) at 100°C in a steamer, washed and then incubated in proteinase K (Thermo Fisher Scientific, AM2546) for RNA target exposure. Tissue sections were post-fixed in 10% neutral-buffered formalin (NBF) and NBF stop buffer (0.1M Tris Base, 0.1 mol/L Glycine, Sigma-Aldrich), and then washed for 5 minutes in PBS. Sections were then incubated overnight at 37°C with GeoMx RNA Probe mix in Buffer R (NanoString Technologies) using a Hybridazer (Dako). Two 25-minute stringent washes were performed in 50% formamide at 37°C. Sections were thereafter washed with 2× SSC and then blocked in Buffer W (NanoString Technologies) for 30 minutes in the humidity chamber at room temperature. Slides were subsequently stained with morphology markers solution (Syto13, PanCK, and CD45^−^; NanoString Technologies) and then loaded on the GeoMx Digital Spatial Profiler (DSP). Slide images were acquired and digitalized with the GeoMx, and 71 circular ROIs with 300 diameter were selected by a breast pathologist. Within each ROI, the GeoMx software was used to define areas of interest (AOI) relying on immunoreactivity to fluorescent markers used: epithelial cell (CK^+^, CD45^−^, Syto13 independent), lymphocytes (CK^−^, CD45^+^, Syto13 independent), and “stroma” (i.e., tumor stroma, CK^−^, CD45^−^, Syto13 independent). After AOI definition, oligonucleotides within each AOI were photocleaved by the DSP and collected. Barcoded oligonucleotides were then dispensed in a 96-well plate, dried overnight, and resuspended in 10 μL of DEPC-treated water. Sequencing libraries were then prepared by PCR with unique i7 and i5 sample indices. Purified and pooled libraries were sequenced at 2 × 27 base pairs and with the dual-index workflow on an Illumina NovaSeq 6000. bcl2fastq2 Conversion Software (Illumina) was used to generate FASTQ sequencing files. GeoMx NGS Pipeline software (v2.3.3.10) was applied to automatically process FASTQ sequencing files to GeoMx readable digital counts (DCC) files.

DSP-provided DCC files were analyzed with GeomxTools R package. Preprocessing was done by filtering the data with segment-based QC, probe-based QC, and limit of quantification followed by data normalization. Segment-based QC involved keeping segments with at least 1,000 reads per segment, at least 80% of aligned, trimmed, and stitched reads, sequencing saturation of more than 50%, and minimum segment area of 5000. To remove gene targets for which there are multiple probes, the next filtering was set to remove probes with a minimum probe ratio of 0.1 and if it is an outlier as per Grubb's test in at least 20% segments. Segments and genes with abnormally low signals were then filtered based on the threshold of at least 5% detection limit. The filtered data were then normalized based on the upper quartile (Q3). Finally, dimensionality reduction and clustering of different ROIs from each segment were performed based on their expression profiles using UMAP projections. Average z-scores of genes in dormancy up, dormancy down, and G_2_–M signatures were then plotted on UMAP for each ROI of CK^+^ segment to show transcriptional heterogeneity in multiregion biopsies across diagnostic and surgical biopsies. Cell type deconvolution was done using the SpatialDecon package using Human Primary Cell Atlas data as reference. Average differences in groups of segments based on a linear mixed-effect model using test variable and random intercept. Volcano plots were labeled by color-coding differentially expressed genes (DEG) in logical comparisons based on their significance levels. DEGs with absolute log fold change >0.5 and *P* < 0.005 were checked for enrichment in MSgiDB Hallmark gene sets using the enrichr package in R.

### Cell Barcoding

The CloneTracker XP 10M Barcode-3′ Library with RFP-Puro (BCXP10M3RP-P) was purchased from Cellecta. Production of lentiviral particles and MCF7 transduction was performed following the CloneTracker XP Lentiviral Expressed Barcode Libraries online manual (https://manuals.cellecta.com/clonetracker-xp-lentiviral-barcode-libraries). Briefly, HEK-293T cells were transfected with Cellecta CloneTracker XP library and ready-to-use lentiviral packaging plasmid mix (Cellecta, CPCP-K2A) using Lipofectamine (Thermo Fisher Scientific, 18324020) and Plus reagent (Thermo Fisher Scientific, 11514015). Viral particles were collected 48 hours upon transfection and precipitated overnight with PEG-IT Precipitation Solution (SBI System Bioscience, LV810A-1-SBI). Lentiviral titration was performed by flow cytometry using RFP as reporter. 10 × 10^6^ MCF7 and T47D cells were transduced with 0.01 multiplicity of infection (MOI) using 0.8 mg/mL polybrene to get a final number of 1–2 × 10^5^ differentially barcoded cells. For selection, 1 mg/mL or 2 mg/mL puromycin (Selleckchem) was added to the culture media of MCF7 and T47D, respectively, for two cycles of 72 hours. Cells were maintained with 0.1 μg/mL (MCF7) or 0.2 μg/mL (T47D) puromycin during the TRADITIOM experimental period.

### TRADITIOM Longitudinal Cell Tracking

Differentially MCF7 barcoded cells were expanded for 13 days from 1 × 10^5^ cells to reach a POT (pretreatment) population of ∼90 × 10^6^ cells and plated based on the following scheme: (i) 34 hyperflasks were seeded (1.2 × 10^6^ and 2.8 × 10^6^ cells for −E2 and TAM conditions, respectively) including Drug Holiday flasks; (ii) 2 × 10^6^ cells were kept in culture as untreated arm (UT, 3 replicates) ; (iii) 8 × 10^6^ were expanded to 90 × 10^6^ and harvested as triplicate POT samples; (iv) 5 × 10^6^ cells were expanded to 40 × 10^6^ cells for plating of 20 time zero (T0) samples (replicates at the onset of treatment reflecting initial seeding density). and collected after 48 hours; (v) the rest of the barcoded MCF7 population was frozen. Cell treatment of the 34 aliases, with either E2 or TAM, started 48 hours after seeding. Harvesting of each HYPERflask was performed at the indicated time points (shared time points for 2 replicates at day 7, day 14, 1 month, 2 months, and diverging time points for individual awakenings). At the time of collection, cells were snap-frozen in multiple pellets for subsequent DNA and RNA extraction (for WGS, genomic barcode sequencing and RNA-sequencing, respectively). Following awakening, TAM α−ζ and AI α−ε samples were further cultured for 1 month in T150 flasks (Corning) with cell passaging giving rise to TEPs. Seve­ral aliquots of cells were frozen at awakening and TEP time points.

Differentially T47D barcoded cells (∼200K barcodes) were expanded for 12 days to reach a POT (pretreatment) population of ∼20 × 10^6^ cells and plated based on the following scheme: (i) 8 HYPERflasks were seeded (1.1 × 10^6^ cells) for −E2, (ii) 1.1 × 10^6^ cells were kept in culture as untreated arm (UT, 3 replicates), (iii) 3.5 × 10^6^ were expanded to 20 × 10^6^ and harvested as triplicate POT samples (5 × 10^6^ cells each) with the rest of the barcoded population frozen, (iv) 4.4 × 10^6^ cells were used for plating of four zero (T0) samples (1.1 × 10^6^) and collected after 48 hours. Estrogen deprivation of the 8 HYPERflask carbon copies (−E2) started 48 hours after seeding. Harvesting of each HYPERflask was performed at the indicated time points (shared time points for 2 replicates of 1 month and diverging time points for individual awakenings). At the time of collection, cells were snap-frozen in multiple pellets for subsequent DNA and histone extraction (for targeted sequencing panel, genomic barcode sequencing, and super-SILAC MS, respectively). Following awakening (early progression), AI A-F samples were further cultured for 1 month in T150 flasks (Corning) with cell passaging giving rise to TEP (late progression). Several aliquots of cells were frozen at awakening and TEP time points.

### Drug Holiday

For drug holiday, two MCF7 carbon copies in HYPERFlasks were reexposed to E2-supplemented media after 1 month (namely, dormancy entry stage) of either tamoxifen (DH 7d TAM) or estrogen deprivation (DH 7d –E2). Cells were monitored for a week for resumption of cell proliferation evaluated by visual inspection using an EVOS cell imaging system (Thermo Fisher Scientific). After 1 week of the drug holiday (when cells started to grow exponentially), ET conditions were reintroduced for these flasks (TAM or estrogen deprivation, respectively). To evaluate the effect of drug holiday on late dormancy, one estrogen-deprived carbon copy was reexposed to estrogen-supplemented media (DH 14d –E2). The exponential growth phase for this carbon copy was spotted after 2 weeks by visual inspection, when the media were reverted to estrogen-deprived condition, thus ending the drug holiday period. Samples for genomic barcode sequencing and RNA-seq were collected at the time of awakening (early progression). Terminal endpoints (TEP; late progression) for carbon copies (replicates) that underwent drug holiday were generated in a similar fashion to other awakenings and proliferation dynamics under drug exposure were measured as described below.

### Drug–Response Curves and Proliferation Assay

UT, TAM, or AI (−E2) TEPs were seeded (1,000 cells per well) in 96-well standard plates (Corning). Following overnight incubation, UT and TAM-TEP cells were treated with 10-fold increasing concentrations of 4-OHT (1 nmol/L–10 μmol/L), vehicle control (EtOH) or reexposure to E2 (10 nmol/L) in five independent replicates. On the other hand, UT and −E2 TEPs underwent several treatment conditions in five independent replicates: −E2, 100 nmol/L TAM, 100 nmol/L fulvestrant (Fulv, Sigma I4409), 50 nmol/L CDK7 inhibitor (CDK7i, kindly provided by Prof. Simak Ali), 100 nmol/L palbociclib (Palbo, SIGMA PD 0332991) and reexposure to E2 (10 nmol/L). The percentage of confluency was assessed and automatically calculated based on the images acquired with IncuCyte Zoom Live-Cell Analysis System (Sartorius) both on the day of compound addition (day 0) and 7 days of incubation in a 37°C and 5% CO_2_ cell culture incubator. Day 7 data were normalized to day 0, and overall data were represented as confluency fold changes over time.

### TRADITIOM LSC

A low-complexity (100 barcodes) MCF7 cell population was generated by subsampling the high-complexity (100K barcodes) founder population to be able to trace every barcode (lineage) over time by scRNA-seq where a maximum of 10K cells can be profiled for each sample. Briefly, 100 cells were seeded and expanded to 8 × 10^6^ cells. The resulting number of barcodes and their frequencies were verified by genomic barcode sequencing (NGS). Cells were seeded based on the following scheme: (i) 16 T75 flasks (Corning) were seeded as 0.35 × 10^4^ and 1 × 10^4^ cells for –E2 (8 flasks) and TAM (8 flasks) conditions, respectively; (ii) 2 T75 flasks were seeded as T0 and collected after 3 days (seeding density = 1.5 × 10^4^ cells); (iii) 1.5 × 10^6^ cells were expanded to 9 × 10^6^ and harvested as triplicate POT (pretreatment) for TRADITIOM LSC and genomic barcodes were analyzed by NGS; (iv) the rest of the low-complexity barcoded MCF7 population was frozen. Cell treatment of the 16 aliases, with either E2 or TAM, started 48 hours after seeding. Harvesting of each flask was performed at the indicated time points (shared time points for 2 replicates at 1 month, 2 months, and diverging time points for 4 individual awakenings for each ET condition). Cells were imaged weekly starting from the onset of ET conditions and monitored until awakening (collection time for scRNA-seq procedure) using IncuCyte Zoom Live-Cell Analysis System (except for initial 42 days for AI sc3 sample for technical reasons). Percentage of confluency was assessed in 108 scanning windows covering each T75 flask and automatically calculated based on the acquired images.

For T47D a low-complexity (∼200 barcodes) cell population was generated by subsampling the high-complexity (200K barcodes) founder population. Cells were seeded based on the following scheme: (i) 5 T75 flasks (Corning) were seeded with 0.5 × 10^4^ cells for –E2 treatment, (ii) 2 T75 flasks were seeded as T0 and collected after 3 days (seeding density = 5 × 10^4^ cells), (iii) the rest of the low-complexity barcoded T47D population was frozen. Cell treatment of the 5 aliases, with E2 deprivation, started 48 hours after seeding. Harvesting of each flask was performed at the indicated time points (shared time points for 2 replicates at 1 month, 3 flasks are still in culture at the time of submission, waiting for awakening events). Cells were imaged weekly starting from the onset of ET and monitored until awakening (collection time for scRNA-seq procedure) using IncuCyte Zoom Live-Cell Analysis System. The percentage of confluency was assessed in 108 scanning windows covering each T75 flask and automatically calculated based on the acquired images.

### Single-Cell RNA-Sequencing Library Preparation

Cells were collected at the indicated time points (T0, early and late dormancy, and awakening) and resuspended in HBSS buffer (Invitrogen, 14065049) supplemented with 0,037% sodium bicarbonate (Gibco), 10 mmol/L HEPES (Gibco), and 0.5% BSA. Single-cell suspensions were generated by passing the cells multiple times through 30 μm and 20 μm separation filters (Miltenyi) in succession. The viability and singularity of cells were determined using a Luna-FL Dual Fluorescence cell counter (Logos Biosystems). 10K single cells were loaded into Chromium Single-Cell Platform (10× Genomics). Library preparation was performed following the Chromium Next GEM Single-Cell 3′ Reagent Kits v3.1 manual. For better detection of expressed Cellecta lineage barcodes in scRNA-seq, a custom PCR approach was implemented. After adaptors ligation and cDNA clean up, libraries were divided into two. Three-fourths of the material was used for standard indexing PCR whereas one-fourth of the material was used for breast cancer–specific amplification using a custom two-step nested PCR. PCR1: 13 cycles (Primers: Cellecta FBPI: GTGACTGGAGTTCAGACGTGTGCTCTTCCGATCTCCGACCACCGAACGCAACGCACGCA, 10X read1: ACACTCTTTCCCTACACGACGCTCTTCCGATCT). PCR2: 6 amplification cycles (standard P5 and P7 10× primers). scRNA-seq libraries were sequenced with 285M reads and breast cancer–specific libraries were sequenced with 15M reads using the NovaSeq 6000 platform (Novogene Cambridge, UK).

### TRADITIOM Live and TRADITIOM Dormancy

Barcoded MCF7 EGFP-NLS cells (100 barcode complexity) were generated via lentiviral transduction of the pTRIP-SFFV-EGFP-NLS plasmid (Addgene, #86677). This was performed following a procedure similar to that of cell barcoding, although now using the pMD2.G (Addgene, #12259) envelope and psPAX2 (Addgene, #12260) packaging plasmids for HEK293T transfection. Finally, efficient EGFP-NLS-expressing clones were selected using fluorescence-activated cell sorting (FACS). Cell seeding density in a 6-well for TRADITIOM Dormancy was 1.5k cells/well (45 replicates) and 4.5k cells/well (45 replicates). All 90 carbon copies were subjected to estrogen deprivation 48 hours post-seeding. Cells were imaged weekly starting from the onset of estrogen deprivation, using IncuCyte Zoom Live-Cell Analysis System. EGFP-NLS signal was used for precise cells counting. Cells were collected at 1 month for genomic barcode detection by NGS. 18 libraries were successfully generated and analyzed.

These cells were also used for the TRADITIOM Live study (1,500 cells/well in 48-well plate format): 12 replicate carbon copies were exposed to estrogen deprivation and imaged, using IncuCyte Zoom Live-Cell Analysis System, twice a week over 5 months to monitor dormancy–awakening dynamics.

For topological determination of awakening dynamics, cell number changes in 9 scanning windows in single 48-wells were measured (cell numbers were calculated with NLS-EGFP reporter) by the IncuCyte Zoom Live-Cell Analysis System. Awakening was defined as wells reaching a confluency of 50%. Awakenings were recorded with the number of days passed from the onset of estrogen deprivation. Awakenings were considered localized (localized awakening: LA) when they originated from 1 scanning window (or 2 when they were detected in adjacent scanning windows and temporal image analysis confirmed the expansion from one to the adjacent scanning window or when they were detected simultaneously in adjacent scanning windows and temporal image analysis confirmed a single awakening expanding in 2 adjacent scanning windows at the same time). They were considered global (global awakening: GA) when they were detected in multiple scanning windows around the same time spanning the entire well. Awakening attempts that regressed in successive scans were coined as failed awakenings (FA), whereas scanning windows with no sign of awakening during 5 months of estrogen deprivation were termed dormant (D). Scanning windows with no cells detected were marked as clearance.

### Barcode Amplification and Next-Generation Library Preparation

Barcoded MCF7 and T47D cell lines were harvested and pelleted at indicated time points (POT (pretreatment), latency (only for MCF7), dormancy, awakening (early progression), and TEP (late progression). Genomic DNA isolation was performed using a DNeasy Blood and Tissue DNA extraction kit (Qiagen) according to the manufacturer's recommendations. Qubit (Life Technologies) was used to quantify genomic DNA. Genomic barcode amplification was performed using Titanium Taq DNA polymerase (Clontech-Takara 639208) with a maximum of 50 ng of DNA per reaction. When DNA extraction resulted in more than 50 ng, multiple reactions were performed to amplify the whole material, and the PCR products were combined before library preparation. The following primer sequences were used for amplification: Fwd: ACCGAACGCAACGCACGCA, Rev: ACGACCACGACCGACCCGAACCACGA. TapeStation 2200 (Agilent) was used to detect 151-bp PCR amplicon including the 48-bp semirandom barcode sequence. After purification with SPRIselect beads (Beckman Coulter), NGS libraries were prepared using the NEBnext Ultra II DNA library preparation kit for Illumina (New England Biolabs) according to the manufacturer's recommendations. Libraries were detected and quantified using TapeStation and Qubit. NGS was performed at Novogene (Cambridge, UK) using the NovaSeq6000 platform (Illumina; paired-end 150 bp).

### WGS of Cell Lines

DNA was extracted using a DNeasy Blood and Tissue DNA extraction kit (Qiagen) according to the manufacturer's recommendations. Qubit (Life Technologies) was used for quantification. Quality control and library preparation (28/30 samples prepared using PCR-free library protocol) were performed by Novogene, where 150 bp paired-end sequencing (30× coverage) was performed on the Illumina NovaSeq6000 platform. Trim Galore (v-0.6.4) was used for adapter trimming of reads. Alignment to the hg38 human genome reference was performed using BWA mem (v-0.7.15; ref. 45). Conversion to binary, removal of PCR duplicates, sorting, and indexing were performed using sambamba (v-0.7.0; ref. 48). Postprocessing and variant identification were performed using the Genome Analysis Toolkit (GATK; v- 4.1.3.0; refs. 46, 47) best practices: adding read groups using picard (v-2.20.6) and base quality recalibration using gatk BaseRecalibrator and gatk ApplyBQSR algorithms. Somatic variant calling was performed on each sample individually using Mutect2 using time 0 bam file POT1 (pretreatment) as normal, using the population germline resource af-only-gnomad.hg38.vcf.gz from the GATK resource bundle, with parameter–af-of-alleles-not-in-resource set as 0.001 and disabling MateOnSameContigOrNoMapped-MateReadFilter filter. Mutect2 variants were filtered using gatk FilterMutectCalls and only PASS mutations were further analyzed. BRCA driver gene mutations were found to have some supporting reads in the generated BAMs; however, they could not be detected *de novo* during the variant calling using Mutect2 due to a lack of sufficient evidence. To complement this, we downloaded a list of all coding variants identified in BRCA tissues deposited in the COSMIC database. We subset these to variants altering genes present in the IntOGen BRCA driver list or the pan-cancer IntOGen driver list.

As the Mutect2 analysis only offers information on *de novo* variants, we also used a germline caller, Platypus (29), on all samples including the POTs (pretreatment), to also identify any preexisting variants that might have undergone selection following the initiation of treatment. Platypus variants were annotated with VEP. All variants for which a Fisher exact test suggested a change in VAF across samples at a significance level of *P* < 0.01 were included. Variants that had an average VAF < 5% were absent from the POTs. Those that had a VAF > 10% were present in a specific sample. Variants that had at least one supporting read in either of the tumor samples were selected, while excluding germline mutations by dropping variants with a VAF > 0.1 in matched patient buffy coats. Using these variants, we produced heat maps outlining significant changes in VAF during treatment of either preexistent or *de novo* mutations found in breast cancer driver genes and all cancer driver genes, respectively. Maximum parsimony phylogenies were reconstructed from mutations absent in the POTs with methods from the R package phangorn (49).

Subsequently, we used the same postprocessed bam files to estimate copy-number profiles. This analysis was performed using sequenza (50). We first produced seqz files using sequenza−utils bam2seqz and then binned them using sequenza−utils seqz_binning with size of windows set to 50. We then added average coverage values of normal WGS reference samples of a study of 30 colorectal cancers (51) to the files and recalculated the depth ratios. Integer copy-number values were then estimated based on the modified depth ratios using sequenza. For this, we used default parameters, nonoverlapping windows of 5e^5^, and a parameter space that was restricted to tumor cell content above 0.9.

### Targeted Sequencing of Cell Lines

DNA from TRADITIOM T47D samples was extracted using a DNeasy Blood and Tissue DNA extraction kit (Qiagen) according to the manufacturer's recommendations. Extracted DNA was sonicated and processed further as described above except for the FFPE DNA-repair part (see “Targeted sequencing of late relapse patients” section) to capture the libraries with the bespoke targeted panel probes. Captured pools were sequenced by Novogene using the NovaSeq6000 platform (Illumina; paired-end 150 bp).

### Noncoding Variant Analysis

Noncoding variants were selected via OpenCravat (all SNV excluding Exome). Noncoding SNVs were filtered with the ENCODE Cis Regulatory Element function and sorted for the LINSIGHT score. Noncoding SNVs called in 3 carbon copies and with a LINSIGHT score >0.4 were overlapped with our unpublished noncoding CRISPR-KRAB Screen (repression of CRE under estrogen deprivation).

### Genomic Barcodes Bioinformatic Analysis

Raw reads were trimmed for adapters and quality (Phred Quality ≥30) with trim_galore(v-0.6.4_dev). After confirming the quality of processed reads with FastQC (version v-0.11.9), they were mapped to the Cellecta CloneTracker XP 10M Barcode library with BWA mem (v- 0.7.17-r1188) using default settings. The alignment maps were then parsed with samtools (v-1.9) to filter out all the reads with supplementary alignments and alignment quality less than 30. The filtered and sorted alignment maps were used to count the number of reads per barcode. For the barcodes supported by at least 10 reads, read counts were normalized with library size for each sample to get barcode frequencies. Heat maps were plotted for all the barcodes with frequency greater than 0.1. The similarity between POTs (pretreatment population) and T0s was shown with correlation plots supported with significance values reported from the Spearman correlation test for each pair.

Survival dynamics was studied based on the number of barcodes with nonzero frequency in each sample. The POT was divided into quartiles to color code and create barcode subsets with low, low–mid, mid–high, and high frequencies. Frequency distribution at dormancy was represented with violin plots where surviving barcodes were color coded based on initial frequencies. The evolution of winner barcodes was represented with the frequency of each winner highlighted on the violin plot in POTs, Dormancy, and the respective awakening hyperflasks.

For TRADITIOM Dormancy, unlike TRADITIOM High, all the barcodes supported with at least 3 reads were considered for further analysis. Distribution of survival ratio (number of barcodes surviving in the 18 wells at month 1 to those observed in the POT) was used to simulate survival dynamics at month 1 with stats package in R. Mean and the standard deviation of the actual distribution were used in *rnorm* function to simulate random normal distribution for 20 and 1,000 instances. Initial frequencies for 100 barcodes were simulated using POT frequency distribution with *sample* function. Random binomial distribution for the corresponding initial cell count was estimated using *rbinom* function with 100,000 as the POT size and simulated frequency as probabilities. To simulate month 1 survival in each instance, POT size was reduced with the simulated survival ratio for that instance. Random binomial distribution was again simulated for month 1 cell counts using *rbinom* function with the initial frequency as probability and the reduced POT size. The number of instances where that barcode had nonzero value were identified and plotted against the initial cell count.

We estimated doubling time for cells during the expansion phase of the experimental design, where we started from ∼100,000 cells with 1 barcode per cell and got ∼90 million cells with a broad range of frequency values after 13 days in culture under unperturbed conditions. Considering in “*nt*” days, a cell expands to 2^*n*−^^1^ cells, to reach “*x*” number of cells in 13 days, the cells need “*t*” days as described below:

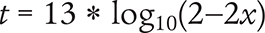


where *t* is the time (in days), and *x* is the number of cells.

Doubling time in hours was calculated using this formula corresponding to all the barcodes in POT samples where the relative number of cells was calculated with frequency values to add up to 90 million in each replicate.

### RNA-Sequencing Analysis

Total RNA was extracted using QIAzol (Qiagen) and the RNeasy Mini Kit (Qiagen). Quality control, mRNA library preparation (polyA enrichment), and sequencing were done at Novogene using the NovaSeq6000 platform (paired-end 150 bp). Raw reads for all the RNA-seq samples were trimmed for adapters and quality (Phred quality ≥30) with trim_galore (v-0.6.4_dev). After quality check with FastQC (v- 0.11.5), the reads were pseudoaligned to reference transcriptome (GRCh38.96) with Kallisto (v-0.46.2; ref. 52). Estimated transcript abundance values reported by “kallisto quant” (number of bootstrap samples = 100) were imported by the “tximport” package in R (v-3.6.1) and gene-level summarization was performed using EnsDb.Hsapiens.v96 (ignoreAfterBar = T, ignoreTxVersion = T). DESeq data set was then created with “DESeqDataSetFromTximport,” and lowly expressed genes were filtered based on having at least 10 counts in more than 3 samples. The filtered data set was then normalized with variance stabilizing transformation (VST) to perform principal component analysis (PCA) using plotPCA function of DESeq2. The filtered data set was then normalized with VST to perform PCA using the plotPCA function of DESeq2.

Differential expression for POT (pretreatment) versus the samples at the latent phase and Dormant versus the awakening samples was estimated with DESeq2 (53). Shrunken log fold changes were calculated, to identify DEGs in all logical comparisons, with the lfcShrink function using “apeglm” as shrinkage estimator. Genes with a fold change of at least 1.5× and adjusted *P* values less than 0.01 were selected as significantly differentially expressed.

DESeq2 Wald statistic (stat) values were used to create the ranked lists of genes based on the expression profiles. These were used to analyze gene set enrichments for up- or downregulated genes in each comparison using gene set enrichment analysis (GSEA) software (v-3.0; ref. 54). The enrichments were observed with a background of hallmark gene sets that represent well-defined biological processes curated by aggregating various MSigDB gene sets (h.all.v7.4.symbols.gmt). Additionally, preadapted SWNE up and down signatures identified by Hong and colleagues (2) were manually added to the hallmark gene sets before performing the enrichment analysis. Significantly enriched gene sets were reported with a false discovery rate (FDR) of 25% as a threshold.

To compare awakening flasks and their TEPs (for which the replicates diverge a lot), we considered each flask independently. Missing the replicate information in this case, we estimated DEGs using the edgeR package (55) with a recommended pipeline for samples without replicates. Common negative binomial dispersion was estimated with “estimateGLMCommonDisp” function with a robust option, “deviance” method, and without a design model. A negative binomial generalized log-linear model was then fit to the read counts for each gene based on the defined contrast. This was followed by the likelihood ratio test with the “glmLRT” function, and the results were then parsed to keep DEGs with *P* value of less than 0.001. A ranked gene list for GSEA in case of these comparisons was created using the reported *P* value multiplied by the sign of the fold change.

For identifying dormancy-associated gene lists, significantly DEGs were selected (abs(log_2_FoldChange)>1 and *P*_adj_ < 0.01) while comparing the expression profiles of replicates during dormancy in both −E2- and TAM-treated arms (AI: days 30, 60, 90 and TAM: days 30, 60) to the POTs. Z-scores were then calculated in this subset of genes across POTs and dormancy samples. For the dormancy-up specific gene list, the genes showing positive z-score ≥0.1-quantile in at least 6 dormancy samples were selected and ordered based on the coefficient of variation and cumulative z-scores. Similarly, the dormancy-down specific gene list was curated with genes showing negative z-scores ≤0.9-quantile value of the distribution in at least 6 dormancy samples. Transcription profile was shown for these genes using a heat map extending the plot to report z-scores across all samples including UT (days 30, 120, and 170), awakening, and TEPs of TAM and −E2 (AI) samples.

### scRNA Sequencing Analysis

Quantification of genes and expressed barcodes was performed with the *cellranger count* pipeline (v-6.0.2) using GRCh38 as the reference transcriptome and a feature reference corresponding to the Cellecta CloneTracker XP 10M Barcode library. The unified filtered feature-barcode matrix was imported into the Seurat package (v-4.1.0; ref. 56) with the *Read10X* function for each sample and the Seurat object was created for at least 200 features detected in at least 3 cells. As very few genes indicate low-quality cells or empty droplets, aberrantly high gene count indicates cell doublets, and extensive mitochondrial contamination indicates low-quality or dying cells, further analysis was done on a filtered matrix after removing cells with very low (<200) or high (outliers) counts for feature RNA and a high percentage of mitochondrial contamination (>20%). Features with high cell-to-cell variations were then identified from the filtered matrix after normalization (log normalization with 10,000 as scaling factor) using “VST” as the selection method. Matrix with top 2,000 highly variable features was then scaled with linear transformation and DoubletFinder package (v-2.0.3; ref. 57) was then used to identify doublets and select only singlets for further analysis.

The Cellecta barcode profile was then added to the Seurat object for singlets using the “Custom” tag. The list of barcodes for each cell was then parsed to identify cells with single or occasional multiple barcodes. Cells with single barcodes were annotated with the corresponding ID. Cells with multiple barcodes were checked for the frequency range of constituent barcodes. For cells with multiple barcodes, the barcodes above 90 percentiles in frequency range were chosen for their annotation. If all the barcodes were supported by the same number of reads and the count was greater than 3, the cell was annotated as “complex,” and all the rest were finally annotated as “others.” Another level of annotation was provided for each cell (only with a new single barcode annotation) marking those with the winning barcode as “winners” and the rest as “others.”

Logical combinations were prepared by merging samples of choice followed by normalization, identifying the most highly variable features and batch-effect correction with fastMNN. Euclidian distance from the first 30 PCs was further used to find neighbors and group cells together with the *findCluster* function. The Uniform Manifold Approximation and Projection (UMAP) method with the first 30 dimensions was used for dimensionality reduction and plotted for selected subsets. Feature plots with gene sets were prepared based on scores derived for each gene set with the *AddModuleScore* function.

To ensure the clustering results are not influenced by cell-cycle genes, cell-cycle phase scores were estimated based on canonical markers for S and G_2_–M phases. The signal was then regressed from the fastMNN-based batch-effect corrected expression matrix while scaling the data. UMAPs were then performed again using the first 10 dimensions. Cycling cells that were initially estimated in G_2_–M or S phase were then subset to plot UMAPs for understanding potential changes in transcriptional profiles of *bona fide* awakening (S/G_2_–M in awakening carbon copies) from FA (cycling cells in dormancy samples). Significantly DEGs (*FindMarkers: p*_val_adj = 0.001, only.pos = TRUE) were identified for all cells during dormancy (yellow and golden clusters) in comparison with cells with winning barcodes at awakening (purple cluster) and vice versa. Functional enrichment analysis was then performed for the identified significant DEGs using enrichR package with MSigDB Hallmark gene sets (overlap count ≥ 3, res$Adjusted.P.value ≤ 0.05).

### Nanopore Data Analysis

Nanopore sequencing was done for 2 POTs, AIα, AIγ, TAMα, and TAMγ. Reads passing the quality control were converted in fasta format and aligned to the Cellecta CloneTracker XP 10M Barcode library using Blast Like Alignment Tool (BLAT v-36; ref. 58). BLAST8 format output was parsed to filter hits with alignment length greater than 45 (93.75% of the barcode length) and alignment percentage greater than 95%.

### MCF7 Geminin-mCherry NLS-GFP Cell Line Generation

To generate retroviral particles for mCherry-Geminin integration, HEK293-GP2 viral packaging cells were seeded into 0.1% gelatin-coated T75 flasks. TransIT-LT1 (mirusbio) transfection reagent:DNA mixture was prepared using 7 μg of pLNCX2-FUCCI plasmid (TakaraBio) and 7 μg of pVSV-G (#138479; Addgene) plasmid, parental MCF7 cells were transfected following the manufacturer's instructions. Retroviral particles were harvested by filtering the media through a 0.45 μm with Millipore syringe filter and adherent target cells were transduced at MOI 0.6, seeded at 300,000 cells/well in a 6-well plate for 48-hour incubation. Cells were expanded and screened for mCherry expression using the EVOS XL Core Imaging System (Thermo Fisher) prior to geneticin treatment (600 μg/mL) over 3 weeks for neomycin selection. To incorporate the NLS-GFP nuclear marker, lentiviral particles were generated using the previously described method, except with HEK293-FT transfected with pTRIP-SFFV-EGFP-NLS plasmid (Addgene, #86677), pMD2.G (Addgene, #12259) envelope and psPAX2 (Addgene, #12260) packaging plasmids. Finally, efficient eGFP-NLS and Geminin-mCherry-expressing cells were selected using FACS.

### Continuous Live IncuCyte Imaging of MCF7 Geminin-mCherry NLS-GFP Cells during −E2 (AI) Treatment

MCF7 Geminin-mCherry NLS-GFP cells were seeded at 350 cells/well in 96-well format in E2+ supplemented conditions for 48 hours before switching to −E2 conditions (AI; using FluoroBrite DMEM as a replacement for standard DMEM). The IncuCyte live-cell analysis system was used to for automated imaging over 3 months, images were acquired once daily at 10× objective. Quantification of mCherry and NLS-GFP fluorescence was performed using automated machine learning algorithms within IncuCyte software.

### Histone PTMs Mass Spectrometry Analysis

Histones were enriched from 0.2 to 4 × 10^6^ MCF7 or T47D cells as previously described (59). Approximately 4 μg of histone octamer were mixed with an equal amount of heavy-isotope labeled histones, which were used as an internal standard (super-SILAC mix; ref. 60) and separated on a 17% SDS-PAGE gel. Histone bands were excised, chemically acylated with propionic anhydride, and in-gel digested with trypsin, followed by peptide N-terminal derivatization with phenyl isocyanate (PIC). Peptide mixtures were separated by reversed-phase chromatography on an EASY-Spray column (Thermo Fisher Scientific), 25-cm long (inner diameter 75 μm, PepMap C18, 2 μm particles), which was connected online to a Q Exactive HF instrument (Thermo Fisher Scientific) through an EASY-Spray Ion Source (Thermo Fisher Scientific). The acquired RAW data were analyzed using EpiProfile 2.0, selecting the SILAC option, followed by manual validation. For each histone-modified peptide, the percentage relative abundance (%RA) for the sample (light channel: L) or the internal standard (heavy channel: H) was estimated by dividing the area under the curve of each modified peptide for the sum of the areas corresponding to all the observed forms of that peptide and multiplying by 100. Light/heavy (L/H) ratios of %RA were then calculated and are reported in Supplementary Table S8. Only peptides that could be reliably quantified in at least 50% of the samples for each condition tested were included in the analysis. The mass spectrometry data have been deposited to the ProteomeXchange Consortium (61) via the PRIDE partner repository with the data set identifier PXD038030.

### Histone Extraction

Cells were harvested with trypsin, centrifuged (12, 000 × *g*, 5 minutes, 4°C) and pellets were washed twice with ice-cold PBS. Histones were extracted using the Abcam histone extraction kit (ab113476), sonicated (2 pulses, 30 seconds, on Bioruptor Pico, Diagenode) during lysis stage and pH adjusted with Balance-DTT buffer. BCA (PierceTM, 23225) was used for histone quantification following the manufacturer's instructions.

### Cell Lysis and Western Blotting

Cell pellets were collected with trypsin, centrifuged (1,200 × *g*, 5 minutes, 4°C), and washed with ice-cold PBS. Protein lysates were obtained by lysing cells in RIPA buffer (15 minutes, on ice) supplemented with protease and phosphatase inhibitors. Lysates were sonicated (2 pulses, 30 seconds, on Bioruptor Pico, Diagenode) and centrifuged (14,000 × *g*, 15 minutes, 4°C) to remove cell debris. Supernatants were collected and protein concentration analyzed by BCA assay (Pierce, 23225). Equal amounts of protein were separated on precast SDS-PAGE gels (Invitrogen Bolt 4%–12% Bis-Tris) in MES running buffer and transferred (wet transfer, 1 hour at 100 V or overnight at 35 V) to methanol activated (5 minutes at RT) PVDF membrane (Immobilon-P). Membranes were blocked with 5% BSA in phosphate-buffered saline containing 0.1% Tween-20 (PBS-T) and incubated with primary antibodies (diluted in 5% BSA in PBS-T) targeting EZH2 (Cell Signaling Technology CS5246), G9a (EHMT2; Cell Signaling Technology CS3306), H4K20me3 (Abcam ab9053), H3K9me2 (Abcam 1220), H3 total (Cell Signaling Technology CS9715), H4 total (Active Motif 61300), and β-actin (Cell Signaling CS 4967). After 4 washes (RT, 10 minutes, PBS-T), membranes were incubated with appropriate horseradish peroxidase-conjugated secondary antibodies (diluted in 1% BSA in PBS-T), membranes were washed (4 washes, 10 minutes at RT in PBS-T), and protein bands were visualized using enhanced chemiluminescence (Pierce ECL) detection. If necessary, membranes were washed in PBS and stripped with Thermo Fisher Restore Western Blot Stripping Buffer (30 minutes, RT), washed in PBS and reblocked for further reprobing.

### Inhibitor Experiments

An optimal number of cells (MCF7: 200 cells per well; T47D: 800 cells per well) was seeded in 96-well standard plates (Corning). Following 2 days of incubation in E2-supplemented media, cells were treated with small-molecule inhibitors (1 μmol/L for MCF7 cells and 1.5 μmol/L for T47D cells) GSK343 (EZH2 inhibitor, Sigma-Aldrich SML0766), HKMTi-1-005 (G9a inhibitor, kindly provided by Robert Brown), A-196 (KMT5B/C inhibitor, Sigma-Aldrich SML1565), or with appropriate vehicle control in six independent replicates and grown in estrogen-supplemented (+E2) or estrogen-deprived (−E2) media. Confluence was automatically assessed and calculated with IncuCyte Zoom software based on the phase contrast images acquired with IncuCyte Zoom Live-Cell Analysis System (Sartorius) both at the day of compound addition (day 0) and indicated days of incubation, in a 37°C and 5% CO_2_ humidified cell culture incubator. Data were normalized to day 0 for each time point, and confluency fold changes over time were used to represent data. One-way ANOVA was used for statistical analysis with the significance level set at *P* < 0.05. Increasing statistical significance is indicated with asterisks (*, *P* < 0.05; **, *P* < 0.01; ***, P < 0.005; ****, *P* < 0.0001). GraphPad Prism version 9.3.1 was used to analyze and visualize the data.

### Survival Analyses

Kaplan–Meier analysis was performed as described previously (62). Three main cohorts were considered for this manuscript. A meta-cohort including several Affymetrix profiled individual cohorts, which were reprocessed as a single cohort, the TCGA cohort, and the METABRIC cohort (63). For the analysis, patients were dichotomized based on the median expression of *EZH2*, *EHMT2*, and *KMT5C*, and a Cox regression analysis was run (where possible, using covariates). The Kaplan–Meier survival plot, and hazard ratio with 95% confidence intervals and log-rank *P* value were calculated and plotted in R using Bioconductor packages.

### Data and Code Availability

Data are available at the Gene-Expression Omnibus under accession number GSE234185. This includes raw and processed data for RNA-seq (GSE234171), genomic barcodes (GSE234174), scRNA-seq (GSE234181), and WGS (GSE234173). The rest of the data can be made available from the corresponding authors upon request.

The code for all the analyses except WGS is accessible at the github repository https://github.com/hd4git/traditiom. The WGS code used in this manuscript is equivalent to the one published in (36, 51).

## Supplementary Material

Supplementary Table 1 Annotation of coding mutations from the patient cohorts and cell lines

Supplementary Table 2 Coverage data for patient profiling

Supplementary Table 3 GeoMx data

Supplementary Table 4 Differentially expressed genes for MCF7 Untreated samples Vs POT

Supplementary Table 5 Differentially expressed genes for MCF7: AI (-E2) latency and dormancy samples Vs POT; awakening Vs Dormancy samples

Supplementary Table 6 Differentially expressed genes for MCF7: TAM latency and dormancy samples Vs POT; awakening Vs Dormancy samples

Supplementary Table 7 Expression matrix for dormancy associated genes

Supplementary Table 8 Mass spectrometry analysis for MCF7 and T47D

Supplementary Figure S1 Genomic profiling and clinical history of late relapse patients.

Supplementary Figure S2 Clinical history of patient 1 (rare cohort treated with long-term ET until progression)

Supplementary Figure S3 Genomic profiling of patients (rare cohort treated with long-term ET until progression)

Supplementary Figure S4 Spatial transcriptomics of patient 1 (rare cohort treated with long-term ET until progression)

Supplementary Figure S5 Spatial transcriptomics of patients 2-3 (rare cohort treated with long-term ET until progression)

Supplementary Figure S6 Spatially resolved changes in the transcriptional profile of tumour-associated immune cells and stroma (Patient 1-3, rare cohort treated with long-term ET until progression)

Supplementary Figure S7 Differential expression profiling of tumour biopsies from spatially resolved CK+ segments of patients 1-3 (rare cohort treated with long-term ET until progression)

Supplementary Figure S8 Traditiom experimental strategy

Supplementary Figure S9 Correlation between barcode composition in Traditiom samples

Supplementary Figure S10 Drug holiday regimen

Supplementary Figure S11 Barcode extinction dynamics of Traditiom samples

Supplementary Figure S12 The de novo dormant persister pool is generated stochastically

Supplementary Figure S13 TRADITIOM WGS Coverage and CNA map

Supplementary Figure S14 Frequencies of winner barcodes (Traditiom samples)

Supplementary Figure S15 Genomic analysis of breast cancer drivers and non-coding alterations in Traditiom samples

Supplementary Figure S16 Morphological inspection of cells along adaptation

Supplementary Figure S17 TRADITIOM MCF7 Bulk RNA-seq

Supplementary Figure S18 Traditiom LSC barcode statistics at single cell level and quality checks

Supplementary Figure S19 TRADITIOM LSC awakening dynamics

Supplementary Figure S20 TRADITIOM MCF7 LSC barcode analysis

Supplementary Figure S21 TRADITIOM MCF7 LSC of the TAM arm

Supplementary Figure S22 TRADITIOM LSC Cell cycle states

Supplementary Figure S23 High frequency barcode occupancy of scRNA-seq clusters

Supplementary Figure S24 Cell cycle regression of TRADITIOM MCF7 LSC for AI (–E2) arm

Supplementary Figure S25 Failed awakening characterization

Supplementary Figure S26 Validation of mass spectrometry and inhibitors’ activity

Supplementary Figure S27 Stratification of BC patients using the epigenetic dormancy signature

Supplementary Figure S28 Expression of epigenetic dormancy signature genes
