# Supplementary material for: Long-term Multimodal Recording Reveals Epigenetic Adaptation Routes in Dormant Breast Cancer Cells
Source: Cancer Discov. 2024 Mar 26;14(5):866–89. doi: 10.1158/2159-8290.CD-23-1161 (PMC11061610; doi:10.1158/2159-8290.CD-23-1161)
Supplement: Supplementary Figure S1 — Genomic profiling and clinical history of late relapse patients. [file cd-23-1161_supplementary_figure_s1_suppsf1.pdf]

Supplementary Figure S1. Genomic profiling and clinical history of late relapse patients

a *Bona fide* Breast cancer drivers

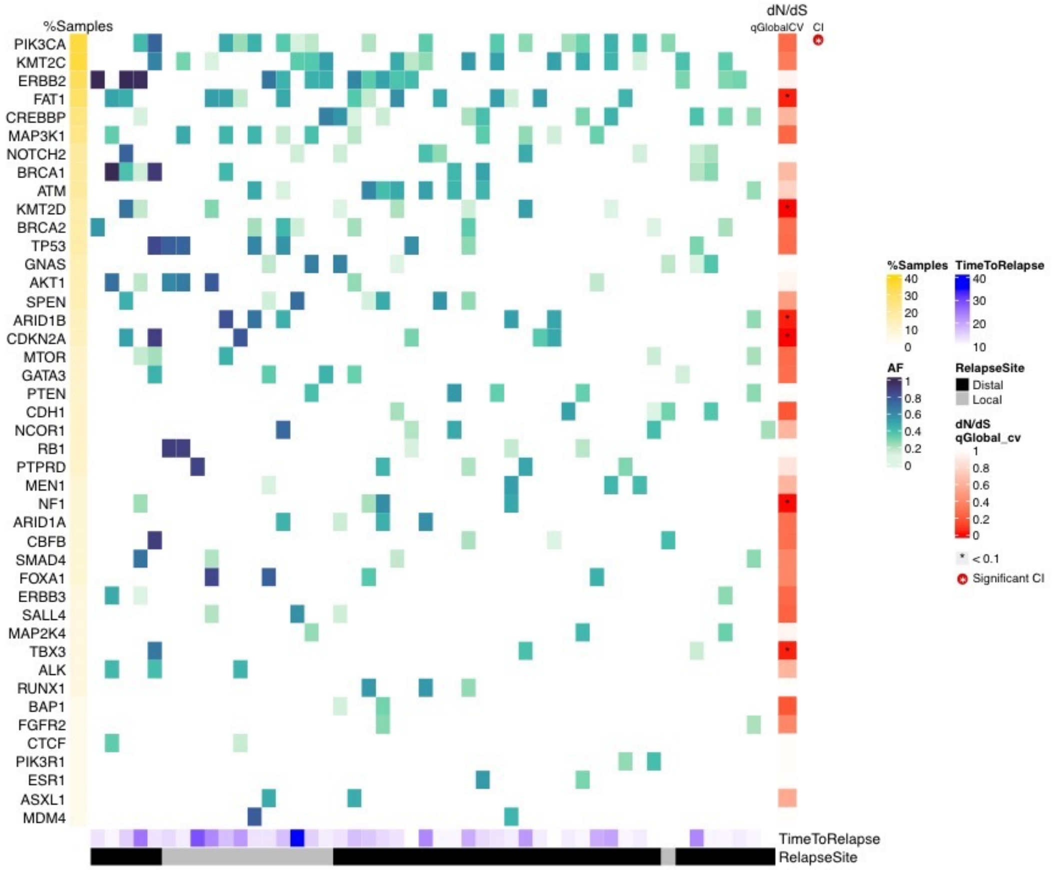

b Late relapses – Clinical details

Patient #	Time to Relapse (y)	Site of relapse	Cellularity (%)
1	11	Pleura	70
2	15	Liver	30
3	12	lymph nodes - supraclavicular	90
4	11	lymph node - axillary	70
5	13	Bone - L5	70
6	14	Breast	40
7	11	Lymph node - axillary	60
8	10	Soft tissue/Skin - Axillary	40
9	10	Skin- Breast (local recurrence)	10
10	12	Soft tissue- subclavicular	70
11	11	Bone - iliac	80
12	15	Bone - iliac	50
13	11	Soft tissue- subclavicular	70
14	13	Liver	80
15	10	Soft tissue - Parasternale	70
16	12	Pleura	50
17	11	Soft tissue- presacrale	60
18	19	lymph node - axillary	60
19	10	Lung	40
20	11	Soft tissue - subclavicular	60
21	11	Pleura	70
22	17	Breast - local	50
23	10	Skin- abdomen	70
24	12	Breast	60
25	13	lymph node - axillary	60
26	14	Lymph node - Axillary	60
27	17	Liver	60
28	15	Breast - Local	10
29	19	Pleura	30
30	35	Breast - local	60
31	29	Breast	70
32	25	Bone - iliac	60
33	14	Lung	30
34	23	Lymph node	30
35	20	Pleura	30
36	12	Skin breast	80
37	13	Lung	30
38	23	Breast	70
39	17	Breast	40
40	21	lymph node- supraclavicular	60
41	10	Ovary	30
42	12	Lymph node- Axillary	60
43	23	Soft tissue - Retroclavicular	60
44	13	Breast	30
45	13	Breast	70
46	16	liver	35
47	12	Breast- left	60
48	21	Breast	70
49	13	Bone - iliac	70

Supplementary Figure S1. Genomic profiling and clinical history of late relapse patients. a) High depth profiling of ER+ BC late relapses using a custom targeted panel. The heatmap shows the mutations of *bona fide* breast cancer drivers that passed the filter for Allele depth >=20, Alternate F1R2+F2R1 >= 4, Allele frequency >= 0.1 and consequence level of moderate or high. Time to relapse, recurrence in the dataset, allele frequency and relapse site are indicated across x and y axes. Significant genes are indicated based on dN/dS analysis from the q-value of neutrality test at gene level (*qglobal_cv <= 0.1) and confidence intervals for the dN/dS ratios per gene (* CI for missense and truncating mutations do not span through value of 1). **b)** Table depicting the time to relapse (in years), site of relapse and cellularity (%) of late relapse samples (n=49).
