# Supplementary material for: Long-term Multimodal Recording Reveals Epigenetic Adaptation Routes in Dormant Breast Cancer Cells
Source: Cancer Discov. 2024 Mar 26;14(5):866–89. doi: 10.1158/2159-8290.CD-23-1161 (PMC11061610; doi:10.1158/2159-8290.CD-23-1161)
Supplement: Supplementary Figure S2 — Clinical history of patient 1 (rare cohort treated with long-term ET until progression) [file cd-23-1161_supplementary_figure_s2_suppsf2.pdf]

Supplementary Figure S2. Clinical history of patient 1

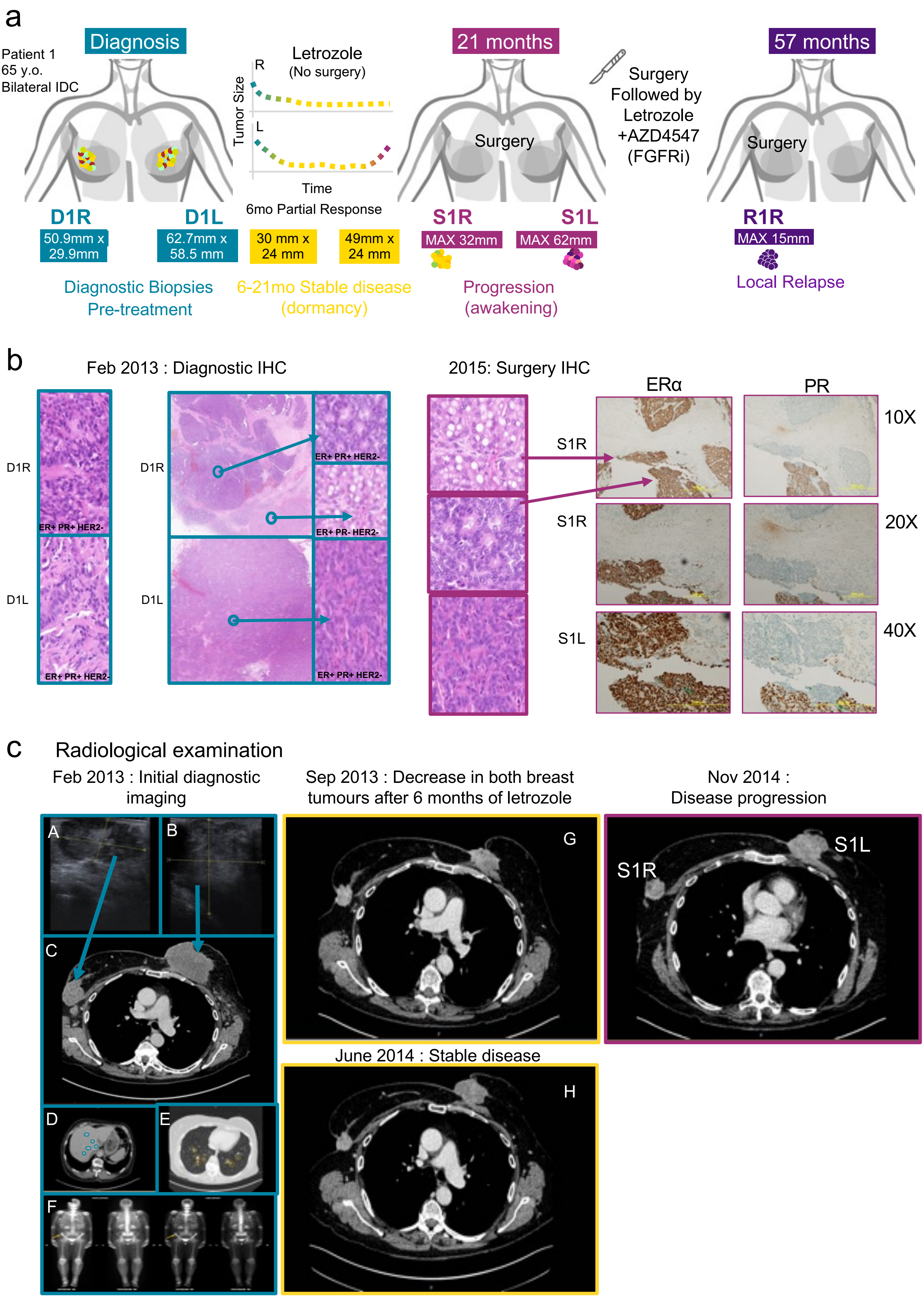

Supplementary Figure S2. Clinical history of patient 1. a) Clinical history of patient 1. IDC=Invasive Ductal Carcinoma, D: diagnostic, S: surgery, R: right, L: left, R1R: relapse in the right breast. **b)** Representative images of H&E and immunohistochemical (IHC) staining of tumours from patient 1 at diagnostic (upper panel) and surgical biopsies (lower panel) (D: diagnostic, S: surgery, R: right, L: left). **c)** Ultrasound scan of patient 1 at diagnosis and after 6 months of ET (letrozole) (A) (February 2013) Ultrasound scan showing the right breast lesion measuring 50.9 mm in maximal diameter. **(B)** Ultrasound scan showing the fungating left breast lesion measuring 62.7mm x 58.5mm. **(C)** Axial section of an intravenous (I.V.) contrast phase computer tomography (CT) scan of the thorax revealing right (52 mm x 41 mm) and left (82 mm x 63 mm) breast tumours. **(D)** Axial section of an I.V. contrast phase CT scan of the liver showing liver metastases highlighted by the yellow circles. **(E)** Axial section of an I.V. contrast phase CT scan of the thorax showing bilateral lung nodules suggesting lung metastases. **(F)** A nuclear medicine whole body bone scan showing uptake of radioactive tracer in the right iliac crest on early and late phase imaging indicative of a solitary sclerotic bone metastatic lesion. The patient was commenced on neoadjuvant letrozole, and a 6 month repeat CT scan (**G)** revealed disease response with the left tumour now measuring 49 mm x 24 mm and the right tumour 30 mm x 24 mm (September 2013). **(H)** A follow-up CT scan in June 2014 showed stable disease with minimal shrinkage of both left and right breast tumours. In December 2014 a further I.V. contrast CT scan revealed evidence of disease progression with the left tumour now measuring 62 mm **(S1L)** and the right 33 mm **(S1R)** in maximal diameter. The patient was then placed on the RADICAL trial with the addition of an FGFR inhibitor. She was then scheduled for a mastectomy and wide local excision for the left and right breast tumours, respectively.
