# Supplementary material for: Long-term Multimodal Recording Reveals Epigenetic Adaptation Routes in Dormant Breast Cancer Cells
Source: Cancer Discov. 2024 Mar 26;14(5):866–89. doi: 10.1158/2159-8290.CD-23-1161 (PMC11061610; doi:10.1158/2159-8290.CD-23-1161)
Supplement: Supplementary Figure S3 — Genomic profiling of patients (rare cohort treated with long-term ET until progression) [file cd-23-1161_supplementary_figure_s3_suppsf3.pdf]

Supplementary Figure S3. Genomic profiling of patients

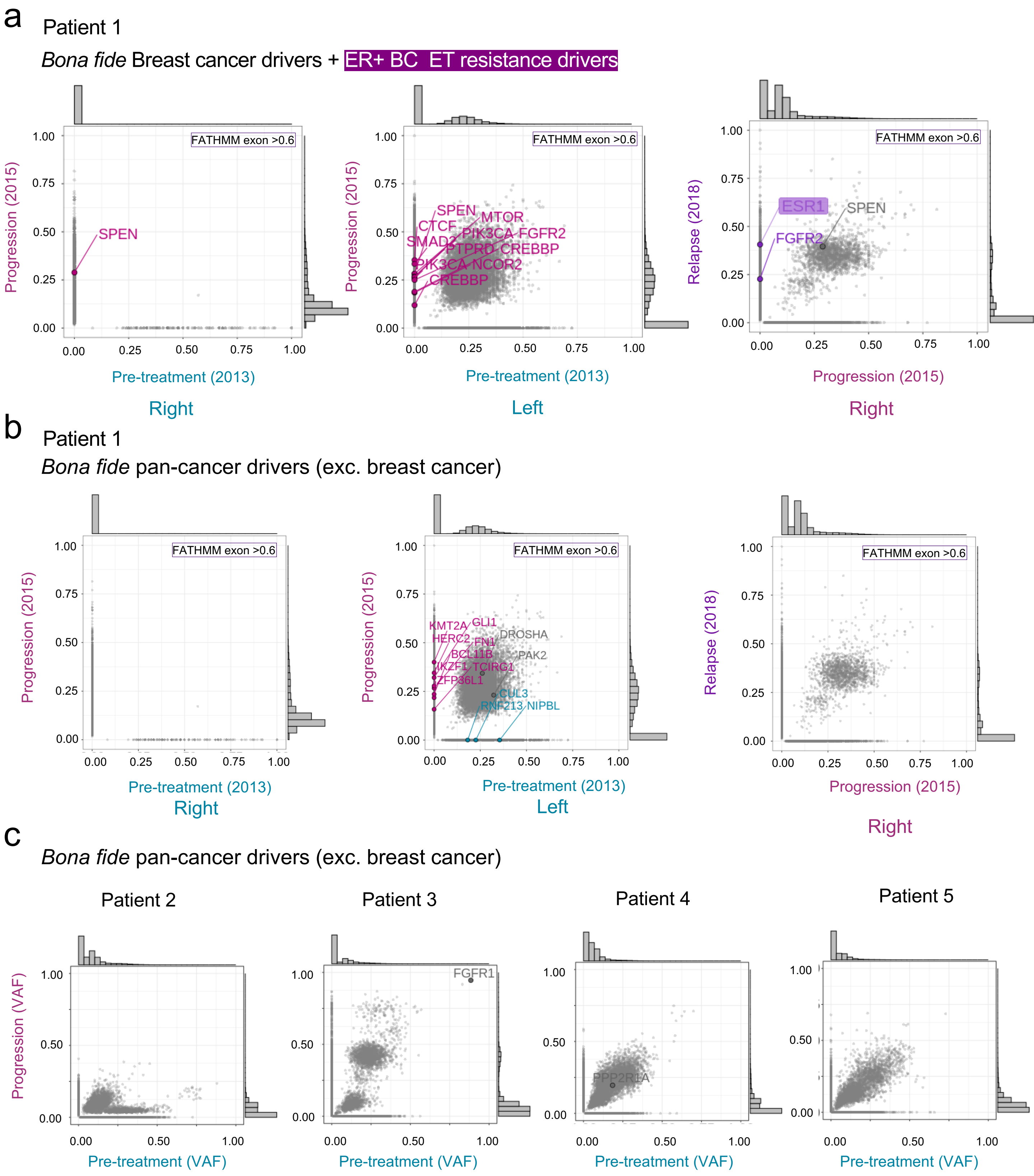

Supplementary Figure S3. Genomic profiling of patients. Variant Allele Frequency plots from WGS for *bona fide* breast cancer drivers and ER+ BC ET resistance drivers for patient 1 **(a)**, known pan-cancer drivers other than breast cancer for patient 1 **(b)** and patients 2-5 **(c)**. Variants are labelled and highlighted according to detection in pre-treatment (teal), progression (magenta), relapse (purple) or both (grey). Labelled genes passed FATHMM significant score >0.6 (predicted damaging). *Bona fide* breast cancer drivers (Intogen) and ER+ BC ET resistance drivers (Bertucci *et al.*) are highlighted and boxed respectively.
