# Supplementary material for: Long-term Multimodal Recording Reveals Epigenetic Adaptation Routes in Dormant Breast Cancer Cells
Source: Cancer Discov. 2024 Mar 26;14(5):866–89. doi: 10.1158/2159-8290.CD-23-1161 (PMC11061610; doi:10.1158/2159-8290.CD-23-1161)
Supplement: Supplementary Figure S4 — Spatial transcriptomics of patient 1 (rare cohort treated with long-term ET until progression) [file cd-23-1161_supplementary_figure_s4_suppsf4.pdf]

Supplementary Figure S4. Spatial Transcriptomics of patient 1

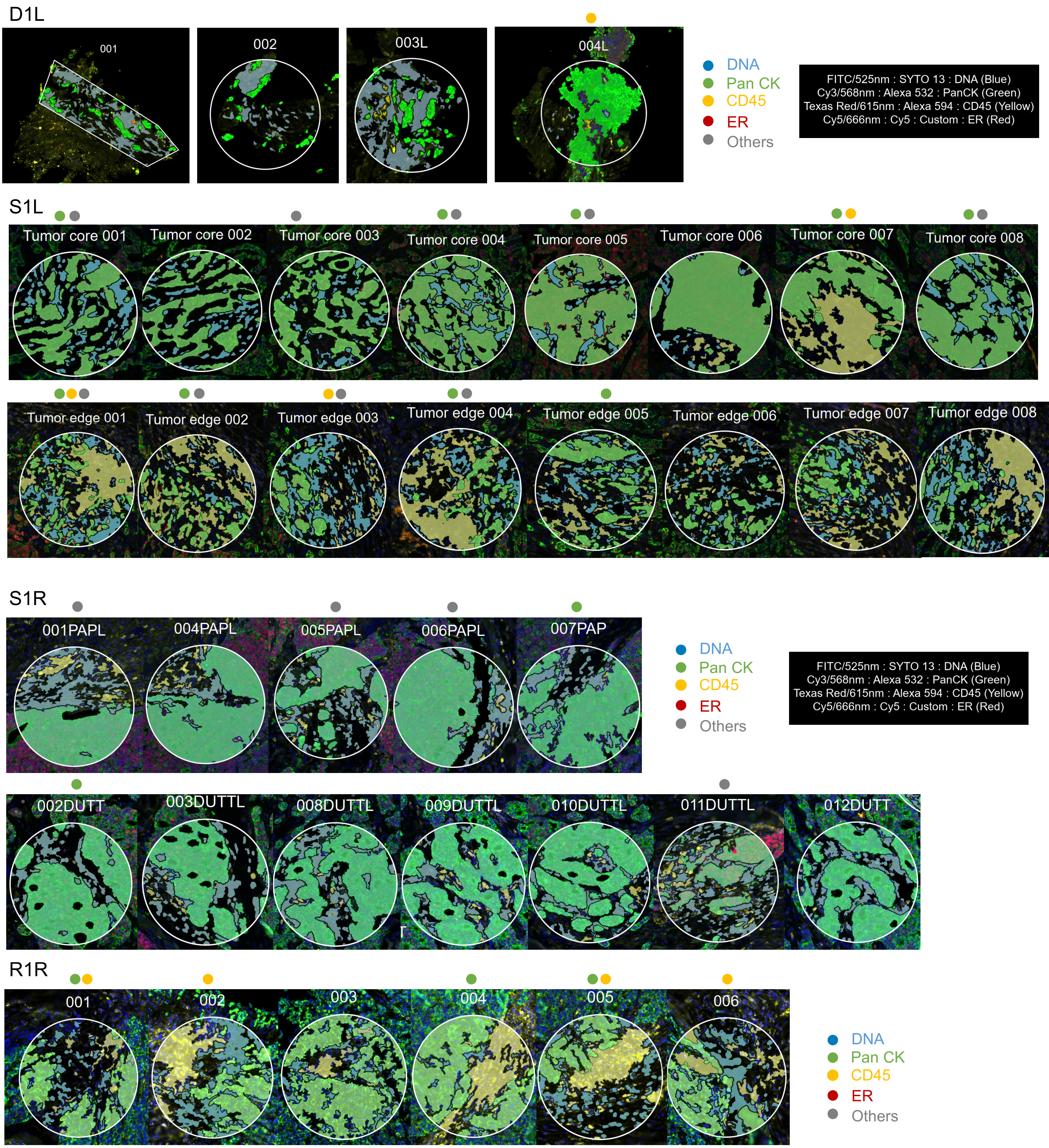

Supplementary Figure S4. Spatial transcriptomics of patient 1. Profiled region of interests (ROIs) of spatial transcriptomics (GeoMx) from patient 1 with relevant stainings (DNA: blue, Pan-cytokeratin: green, CD45: yellow, ER: red, Others: grey). D1L: diagnostic biopsy left, S1L: surgery biopsy left, S1R: surgery biopsy right, R1R: relapse biopsy right. PAPL: papillary, DUTTL: ductal. The dots over each image represent the ROI from corresponding segments that passed the QC.
