# Supplementary material for: Long-term Multimodal Recording Reveals Epigenetic Adaptation Routes in Dormant Breast Cancer Cells
Source: Cancer Discov. 2024 Mar 26;14(5):866–89. doi: 10.1158/2159-8290.CD-23-1161 (PMC11061610; doi:10.1158/2159-8290.CD-23-1161)
Supplement: Supplementary Figure S5 — Spatial transcriptomics of patients 2-3 (rare cohort treated with long-term ET until progression) [file cd-23-1161_supplementary_figure_s5_suppsf5.pdf]

Supplementary Figure S5. Spatial Transcriptomics of patients 2-3

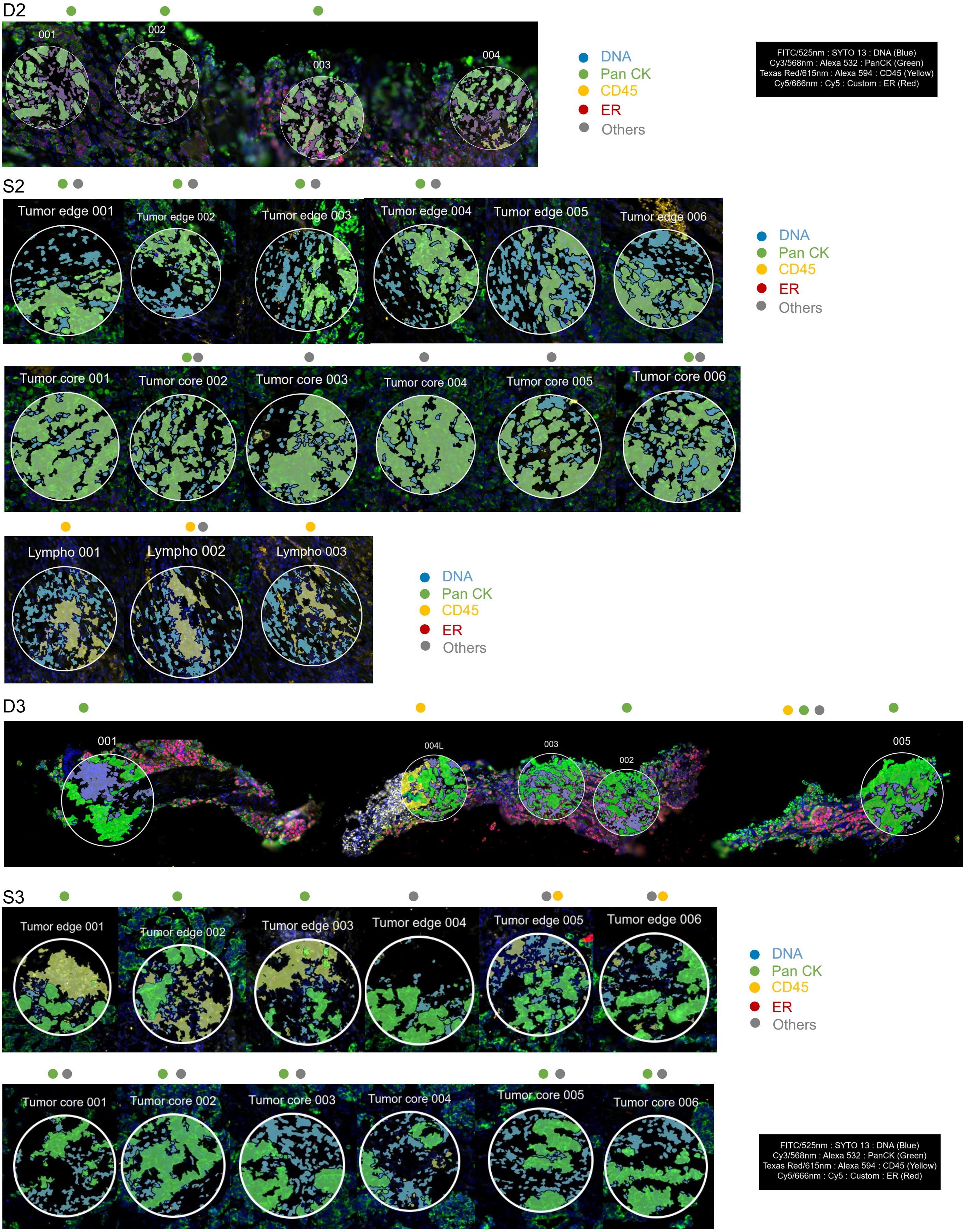

Supplementary Figure S5. Spatial transcriptomics of patients 2-3. Profiled region of interests (ROIs) of spatial transcriptomics (GeoMx) from patients 2 and 3 with relevant staining (DNA: blue, Pan-cytokeratin: green, CD45: yellow, ER: red). D2: diagnostic biopsy (patient 2), S2: surgery biopsy (patient 2), D3: diagnostic biopsy (patient 3), S3: surgery biopsy (patient 3), Lympho: Lymph node. The dots over each image represent the ROI from corresponding segments that passed the QC.
