# Supplementary material for: Long-term Multimodal Recording Reveals Epigenetic Adaptation Routes in Dormant Breast Cancer Cells
Source: Cancer Discov. 2024 Mar 26;14(5):866–89. doi: 10.1158/2159-8290.CD-23-1161 (PMC11061610; doi:10.1158/2159-8290.CD-23-1161)
Supplement: Supplementary Figure S6 — Spatially resolved changes in the transcriptional profile of tumour-associated immune cells and stroma (Patient 1-3, rare cohort treated with long-term ET until progression) [file cd-23-1161_supplementary_figure_s6_suppsf6.pdf]

**Supplementary Figure S6. Spatially resolved changes in the transcriptional profile of tumour-associated immune cells and stroma**

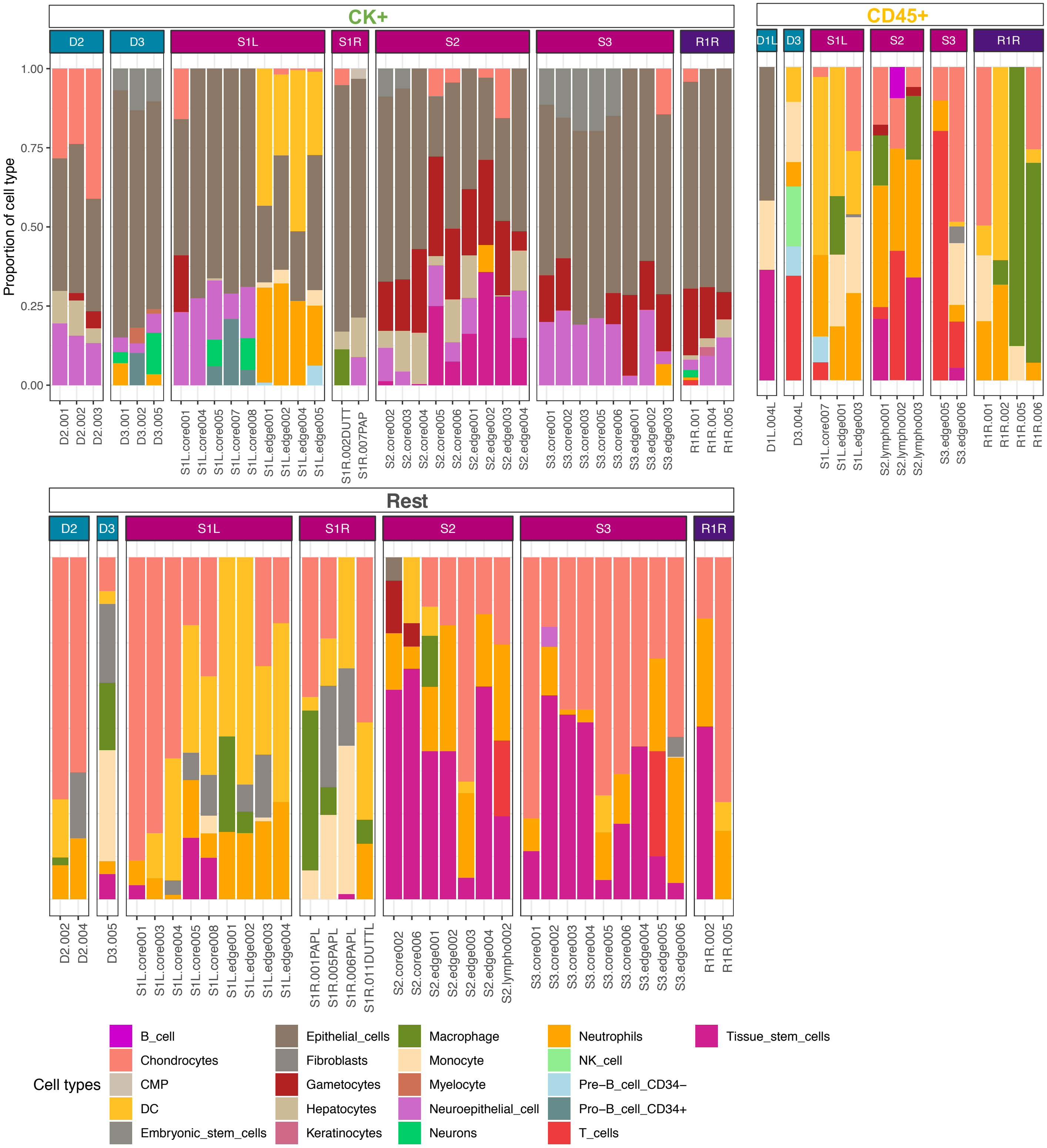


**Supplementary Figure S6. Spatially resolved changes in the transcriptional profile of tumour-associated immune cells and stroma.** Deconvolution of cell types estimated from gene expression profiles in each region of interest according to relevant staining (CK+: tumour cells, CD45+: immune cells, rest: stroma) using signatures from human protein cell atlas (HPCA) as reference. Patient1 (D1L: diagnostic biopsy left, S1L: surgery biopsy left, S1R: surgery biopsy right, R1R: relapse biopsy right) Patient2 (D2: diagnostic biopsy, S2: surgery biopsy), Patient3 (D3: diagnostic biopsy, S3: surgery biopsy).
