# Supplementary material for: Long-term Multimodal Recording Reveals Epigenetic Adaptation Routes in Dormant Breast Cancer Cells
Source: Cancer Discov. 2024 Mar 26;14(5):866–89. doi: 10.1158/2159-8290.CD-23-1161 (PMC11061610; doi:10.1158/2159-8290.CD-23-1161)
Supplement: Supplementary Figure S8 — Traditiom experimental strategy [file cd-23-1161_supplementary_figure_s8_suppsf8.pdf]

Supplementary Figure S8. Experimental design

a

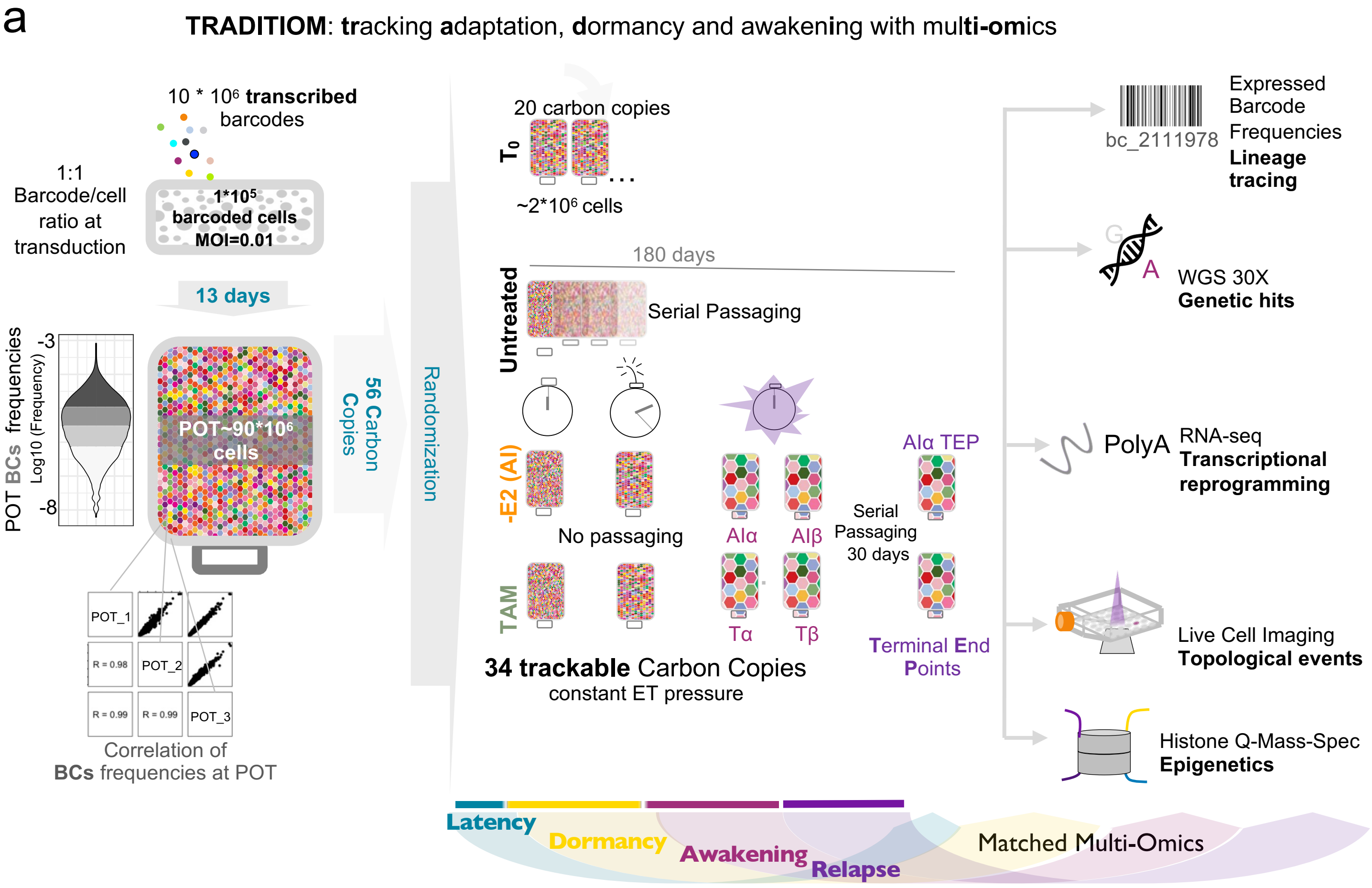

b

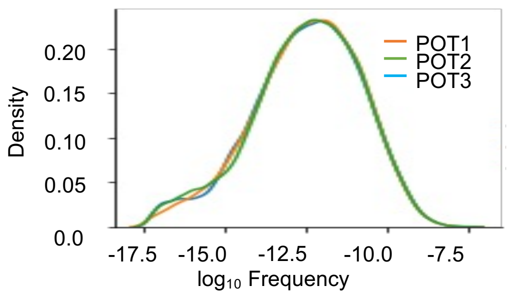

c

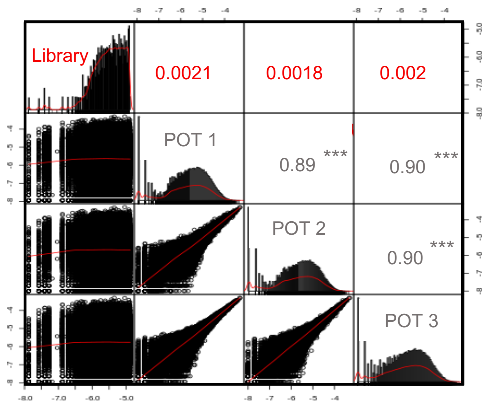

Supplementary Figure S8. Experimental design. a) Schematic representation of TRADITIOM experimental setup. Differentially barcoded cells were expanded from $1*10^5$ to a POT (pre-treatment) population of $90*10^6$ cells. Cells were replated in 56 HYPERflasks (carbon copies, replicates), exposed to either –E2 (oestrogen deprivation) or TAM (tamoxifen) and never passaged during the whole experimental course (3 of the HYPERflasks were subjected to drug holiday regimen– 1xTAM and 2x-E2). In parallel, cells were kept in culture as untreated (UT) counterpart and were subjected to serial passaging for 180 days. Part of the founder population was collected in 3 POT samples to analyse the initial barcode composition and replated in 20 time zero (T0) samples (reflecting the seeding of HYPERflasks) to determine the initial overlap between barcode composition in carbon copies. The experimental design focused on four stages of adaptation: latency (time between treatment onset and dormancy entry at whole population level), dormancy, awakening (early progression) and relapse (late progression, cell passaged for 1 month after awakening). Harvesting of each HYPERflask was performed at the indicated time points (shared timepoints for 2 replicates for latency and dormancy and diverging time points for individual awakenings and terminal end points -TEPs (late progression). Collected samples were analysed to reconstruct lineage adaptation dynamics (genomic barcode analysis), genetic contribution to therapy resistance (WGS), transcriptional reprogramming under adaptation (RNA-seq) and Histone Modification Mass Spectrometry. **b)** Overlap between barcode frequency distributions in the 3 POTs. **c)** Correlation plot for barcode frequency distribution (diagonal) among POT samples and the original plasmid library used to transduce MCF7 cells supported with significance levels from Spearman correlation test (upper triangle). POT samples show correlation among each other while they do not correlate with the plasmid library.
