# Supplementary material for: Long-term Multimodal Recording Reveals Epigenetic Adaptation Routes in Dormant Breast Cancer Cells
Source: Cancer Discov. 2024 Mar 26;14(5):866–89. doi: 10.1158/2159-8290.CD-23-1161 (PMC11061610; doi:10.1158/2159-8290.CD-23-1161)
Supplement: Supplementary Figure S9 — Correlation between barcode composition in Traditiom samples [file cd-23-1161_supplementary_figure_s9_suppsf9.pdf]

Supplementary Figure S9. Correlation between barcode compositions in Traditiom samples

Correlation of BC frequencies between POTs and T_0

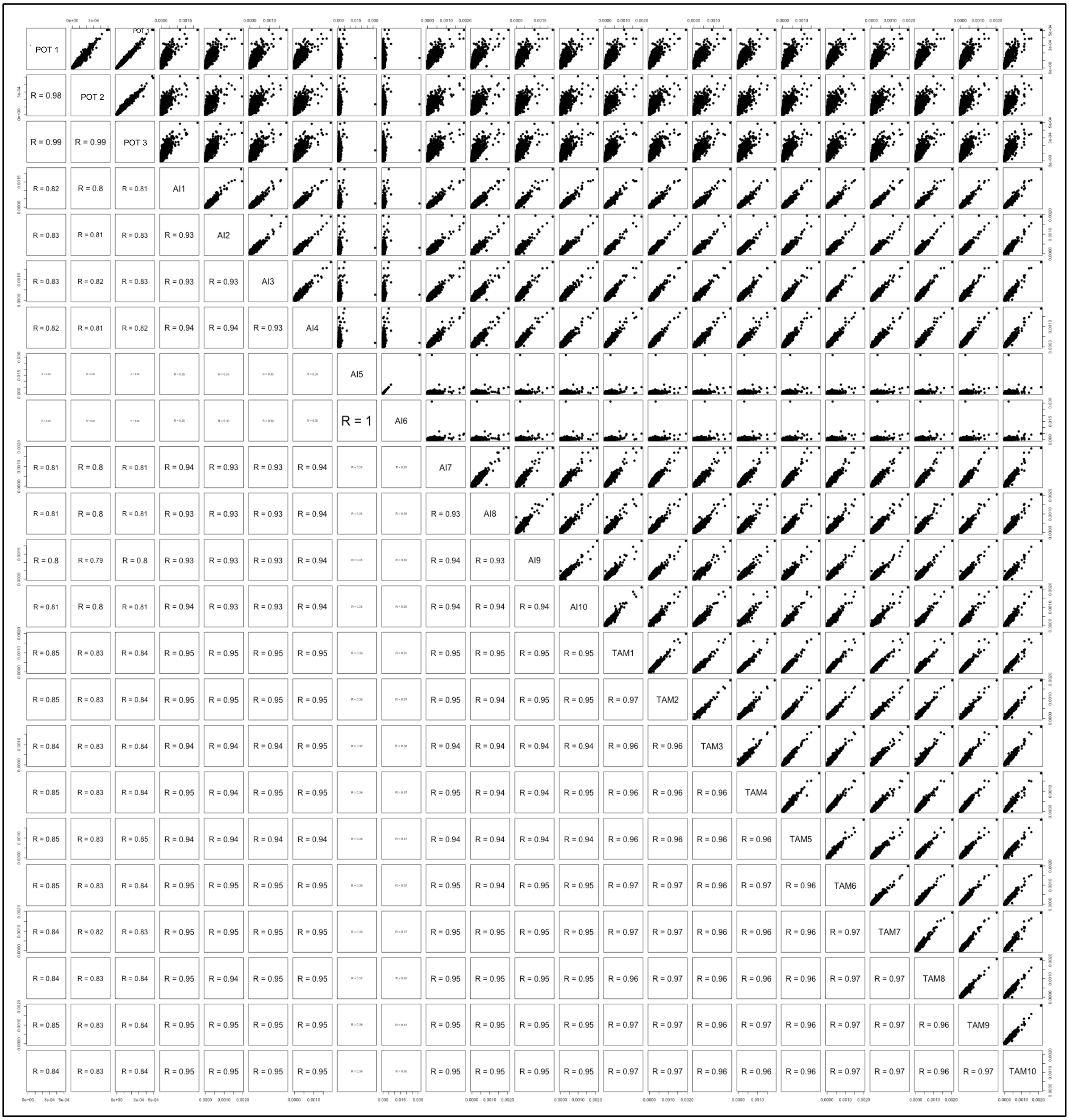

Supplementary Figure S9. Correlation between barcode compositions in Traditiom samples. Ranked correlation between barcode composition in 3 POTs (pre-treatment) and 20 time zero (T0) replicates (upper triangle: correlation plots; diagonal: sample names; lower triangle: significance values from Spearman correlation test). AI1-10 T0 reflect Hyperflask seeding in –E2 flasks. TAM 1-10 T0 reflect seeding in TAM Hyperflasks (Technical problems effected the analysis of two samples -AI5 and AI6-).
