# Supplementary material for: Long-term Multimodal Recording Reveals Epigenetic Adaptation Routes in Dormant Breast Cancer Cells
Source: Cancer Discov. 2024 Mar 26;14(5):866–89. doi: 10.1158/2159-8290.CD-23-1161 (PMC11061610; doi:10.1158/2159-8290.CD-23-1161)
Supplement: Supplementary Figure S10 — Drug holiday regimen [file cd-23-1161_supplementary_figure_s10_suppsf10.pdf]

Supplementary Figure S10. Drug Holiday

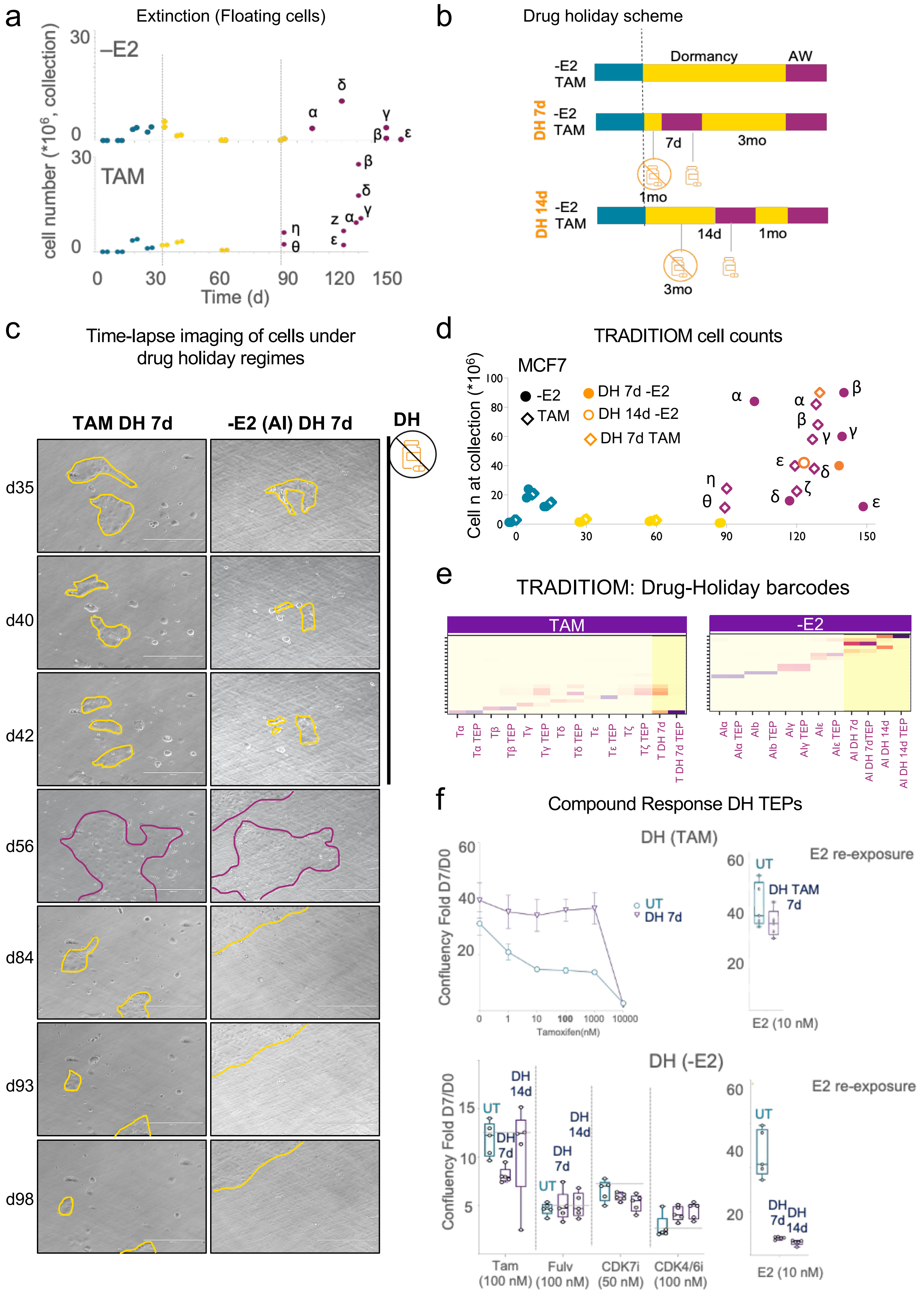

Supplementary Figure S10. Drug holiday. a) Floating cell counts in HYPERflasks for MCF7 –E2 and TAM arm at their respective time of collection (teal: latency, time between onset of treatment and dormancy entry, yellow: dormancy, magenta: awakening (early progression)). **b)** Pictogram explaining alternative drug holiday regimes used in TRADITIOM study. Drug holiday (E2 re-exposure) was introduced at early (30days, for both TAM and –E2) and late dormancy (90days, for –E2). Samples were reverted to drug treatment when cell proliferation was detected by visual inspection and collected at their respective awakening. **c)** Representative bright-field images of MCF7 –E2 and TAM HYPERFlasks (observable layers) along drug holiday (DH) entry and exit (7 days-long drug holiday for both arms). Progressive Images of the same area of the two flasks were captured with EVOS Cell Imaging Systems (10X). Dormancy and proliferation are highlighted by yellow and magenta outlines respectively. **d)** Cell counts for MCF7 HYPERFlasks for –E2 (circle) and TAM (diamond) arms at their respective time of collection (teal: latency, yellow: dormancy, magenta: awakening) including drug holiday samples (orange). **e)** Barcode enrichment analysis: heatmap of barcodes with high frequencies (frequency >=0.1) in carbon-copies (replicates) for both DH TAM and –E2 arm at the time of awakening (early progression) and their corresponding TEPs (Terminal End Points, late progression), along with winners in other TRADITIOM carbon copies and their respective TEPs. **f)** Tamoxifen resistance analysis of the DH TAM TEP to increasing doses of 4-OHT is depicted at the upper left panel. Growth rates of DH –E2 TEPs in response to treatment with different drugs: Tamoxifen (Tam, 4-OHT), Fulvestrant (Fulv), CDK7 inhibitor (CDK7i), CDK4/6 inhibitor (Palbociclib) are depicted at the bottom left panel. Growth dynamics of DH TAM TEPs (upper right) and DH –E2 TEPs (bottom right) when exposed to oestrogen (E2, 10nM) re-introduction are shown as separate plots. Representative graphs are shown as normalized confluency fold change upon 7 days of compound treatment (n=3).
