# Supplementary material for: Long-term Multimodal Recording Reveals Epigenetic Adaptation Routes in Dormant Breast Cancer Cells
Source: Cancer Discov. 2024 Mar 26;14(5):866–89. doi: 10.1158/2159-8290.CD-23-1161 (PMC11061610; doi:10.1158/2159-8290.CD-23-1161)
Supplement: Supplementary Figure S11 — Barcode extinction dynamics of Traditiom samples [file cd-23-1161_supplementary_figure_s11_suppsf11.pdf]

**Supplementary Figure S11. Barcode extinction dynamics in Traditiom samples**

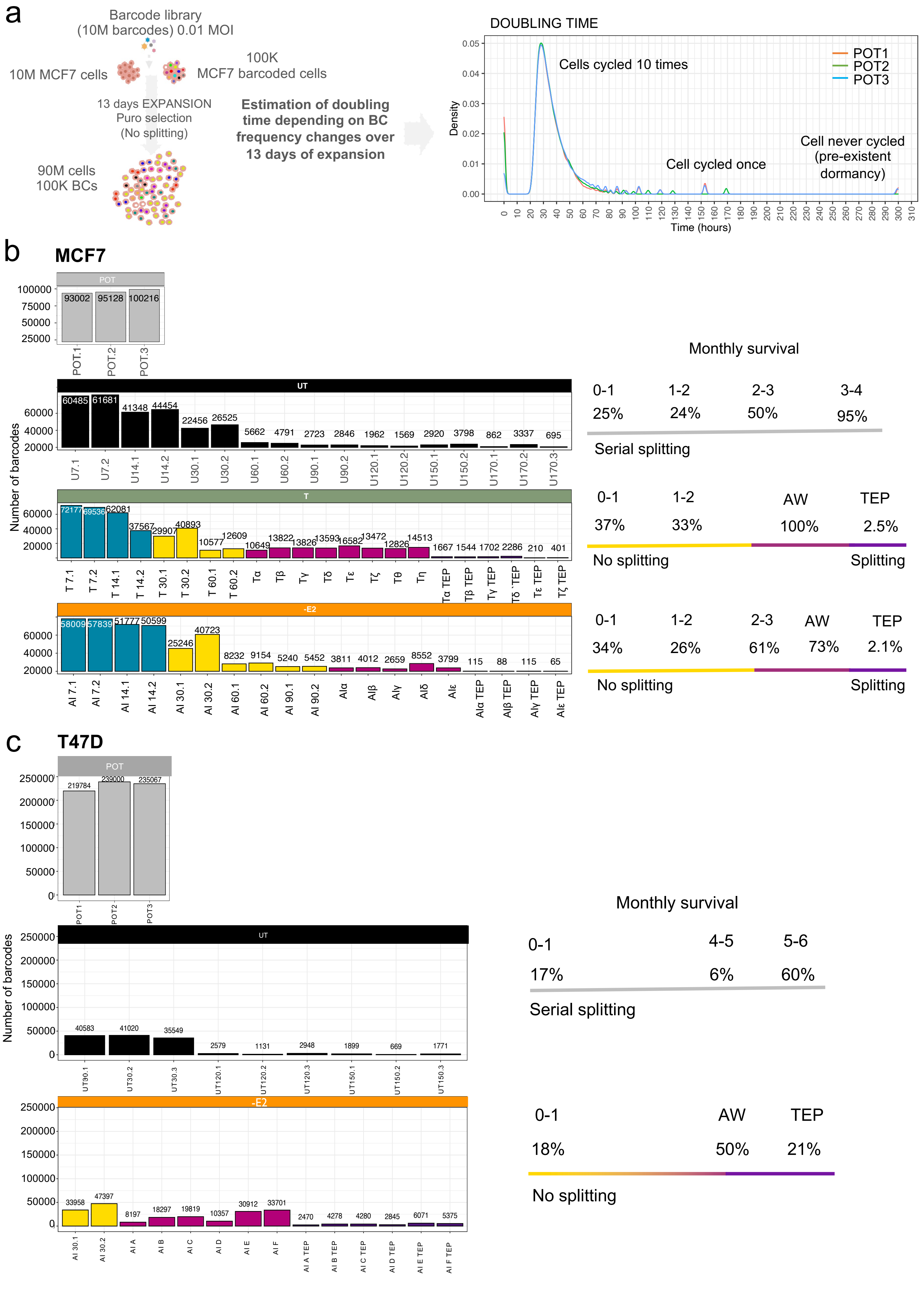

**Supplementary Figure S11**. **Barcode extinction dynamics in Traditiom samples. a)** Density plot of relative barcode abundance across estimated doubling time shows overall consistency in POT (pre-treatment) replicates (with a slight lag in POT2) and indicates presence of cells cycling 10 times (doubling time ~30h), very slow cycling cells that might have undergone cell cycle only once (doubling time ~150h) and cells that might not have cycled (doubling time ~300h) during 13 days of expansion between transduction and POT. **b)** Number of initial barcodes at POT and number of surviving barcodes at each indicated time point, are listed for UT (untreated), TAM (tamoxifen) and –E2 (oestrogen deprivation (mimicking AI)) arms of MCF7 including their respective TEPs (terminal end points, late relapse). **c)** Number of initial barcodes at POT and number of surviving barcodes at each indicated time point, are listed for UT and -E2 arms of T47D including their respective TEPs. A summary of the percentages of surviving barcodes along the collection points is shown on the right side of the panel.
