# Supplementary material for: Long-term Multimodal Recording Reveals Epigenetic Adaptation Routes in Dormant Breast Cancer Cells
Source: Cancer Discov. 2024 Mar 26;14(5):866–89. doi: 10.1158/2159-8290.CD-23-1161 (PMC11061610; doi:10.1158/2159-8290.CD-23-1161)
Supplement: Supplementary Figure S12 — The de novo dormant persister pool is generated stochastically [file cd-23-1161_supplementary_figure_s12_suppsf12.pdf]

Supplementary Figure S12. The *de novo* dormant persister pool is generated stochastically

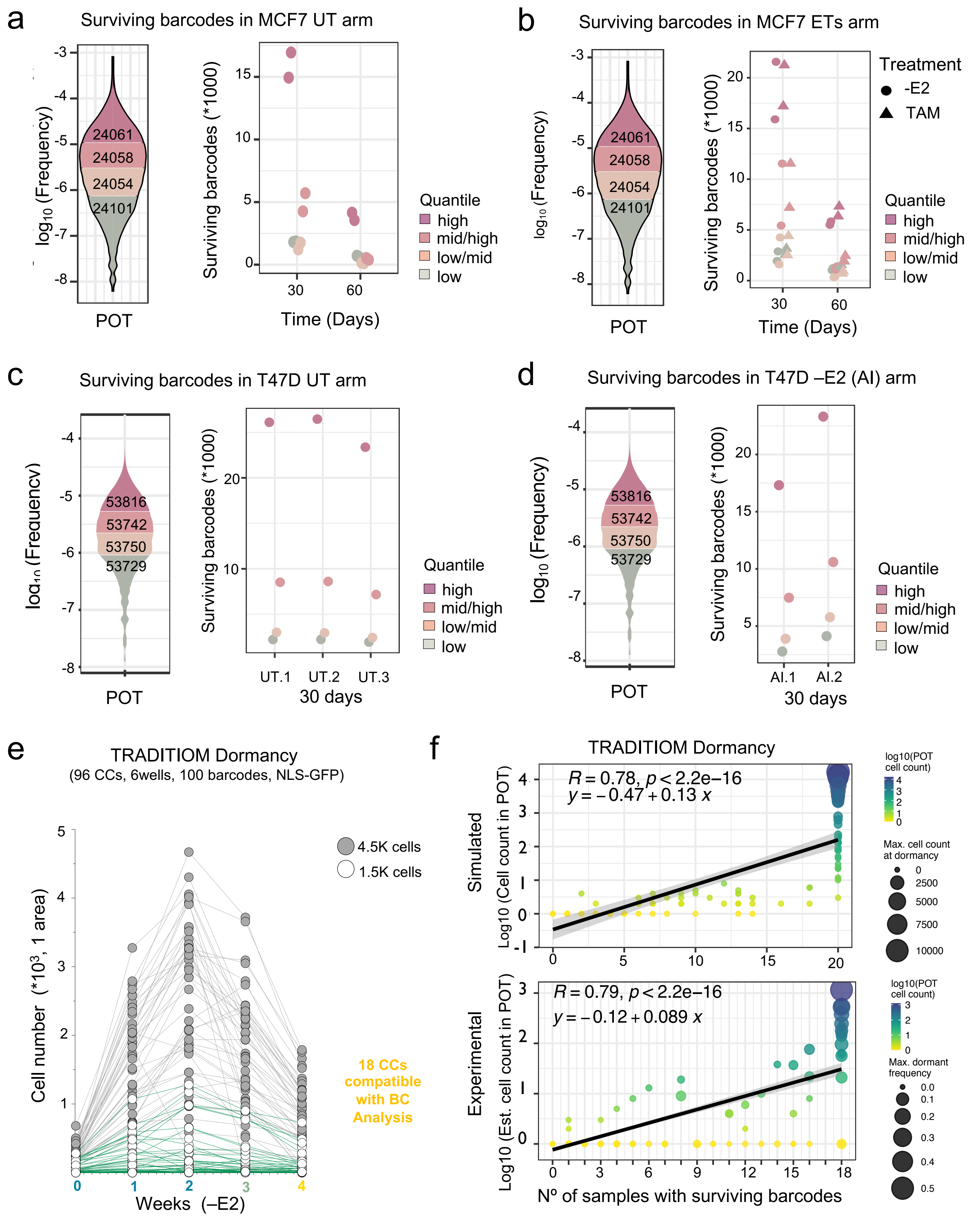

Supplementary Figure S12. The *de novo* dormant persister pool is generated stochastically. BC frequency distribution is shown for POT (pre-treatment) (number of BCs for each quartile is shown) along with number of surviving BCs for each quartile for MCF7 UT (untreated cells at 30 and 60 days) **(a)**, both treatment arms at dormancy (MCF7, TAM and –E2) **(b)**, T47D UT (untreated cells at day 30) **(c)**, and T47D –E2 treatment arm at dormancy **(d).** The initial frequency of barcodes at POT influences the probability of dormancy entry (30days) and maintenance (60days). Barcode composition in dormant samples' replicates is colour coded based on their frequency in POT. Dormant cell pools are constituted mainly by high/ mid-high frequency barcodes. **e)** TRADITIOM Dormancy: cells were seeded in 6-well format, 1.5k cells/well (45 replicates) and 4.5k cells/well (45 replicates). All 90 carbon copies were subjected to oestrogen deprivation (-E2) for 4 weeks to reach dormancy. Cells were imaged weekly starting from the onset of oestrogen deprivation, using Incucyte Zoom Live-Cell Analysis System. EGFP-NLS signal was used for precise cell counting in 1 scanning window per well. **f)** TRADITIOM Dormancy cells were collected at 1 month for genomic barcode sequencing. 18 libraries for genomic barcodes were successfully generated and analysed. Simulation of dormancy entry in a stochastic scenario for 100 BCs with initial POT size of 100K cells in 20 instances (iterations) (upper panel). Simulated data show that the BCs survive in accordance with their initial frequencies. Experimental data confirms that the barcodes which reproducibly entered dormancy in all the replicates were all present at high frequency in POT (lower panel).
