# Supplementary material for: Long-term Multimodal Recording Reveals Epigenetic Adaptation Routes in Dormant Breast Cancer Cells
Source: Cancer Discov. 2024 Mar 26;14(5):866–89. doi: 10.1158/2159-8290.CD-23-1161 (PMC11061610; doi:10.1158/2159-8290.CD-23-1161)
Supplement: Supplementary Figure S13 — TRADITIOM WGS Coverage and CNA map [file cd-23-1161_supplementary_figure_s13_suppsf13.pdf]

Supplementary Figure S13. TRADITIOM WGS Coverage and CNA map

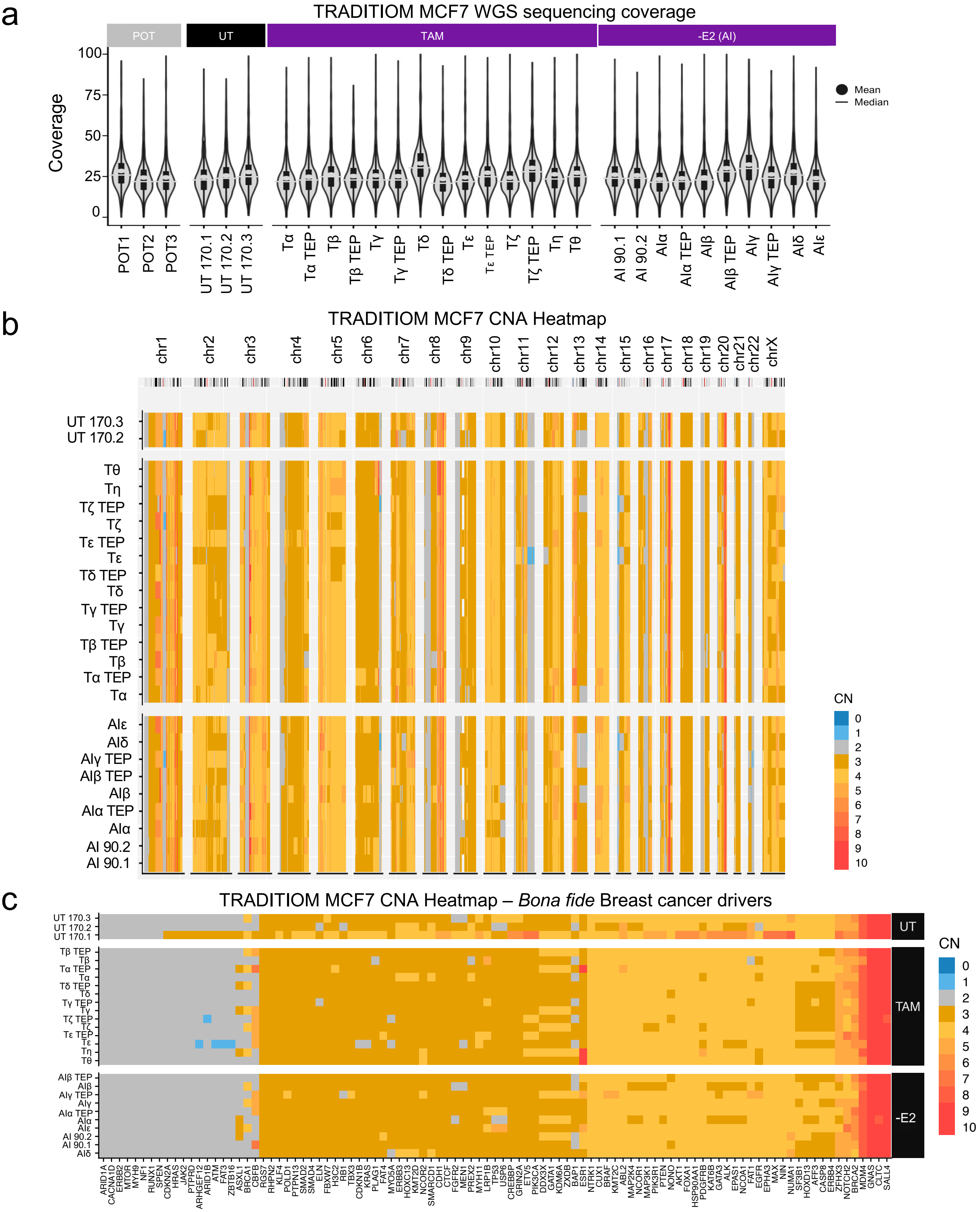

Supplementary Figure S13. TRADITIOM WGS Coverage and CNA map. a) Sequencing coverage distributions (WGS) for all sites across TRADITIOM POT (pre-treatment) samples, awakening (early progression) and TEP (late progression) carbon copies (replicates). **b)** Genome wide heatmap of copy number alterations (CNAs) across carbon copies. **c)** Heatmap for CNAs across TRADITIOM MCF7 WGS dataset for *bona fide* breast cancer drivers. CNA profiles were obtained using sequenza (read-depth approach) against a panel of 30 normal samples from Heide *et al.* 2022 (Nature).
