# Supplementary material for: Long-term Multimodal Recording Reveals Epigenetic Adaptation Routes in Dormant Breast Cancer Cells
Source: Cancer Discov. 2024 Mar 26;14(5):866–89. doi: 10.1158/2159-8290.CD-23-1161 (PMC11061610; doi:10.1158/2159-8290.CD-23-1161)
Supplement: Supplementary Figure S14 — Frequencies of winner barcodes (Traditiom samples) [file cd-23-1161_supplementary_figure_s14_suppsf14.pdf]

Supplementary Figure S14. Frequencies of winner barcodes

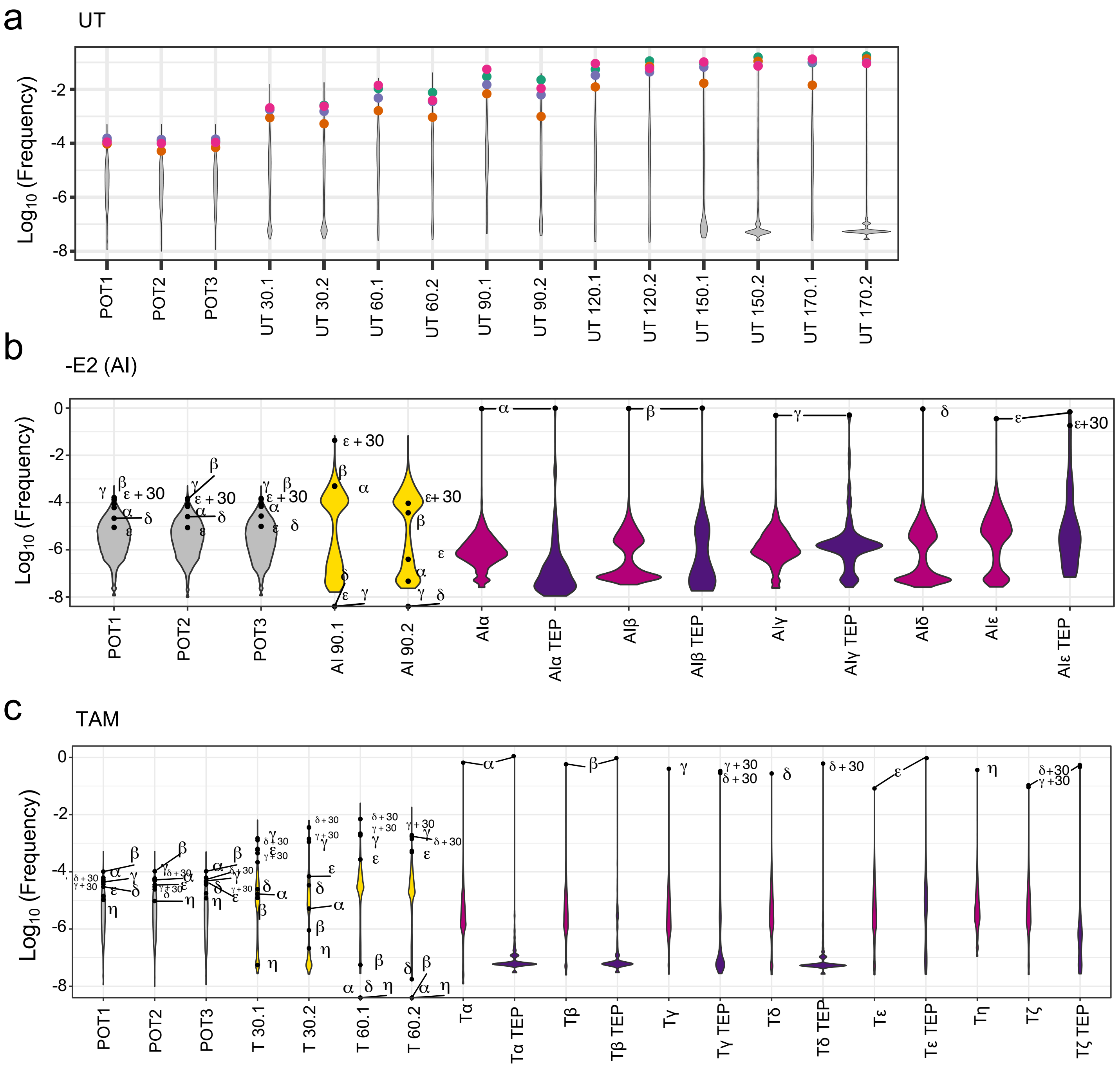

Supplementary Figure S14. Frequencies of winner barcodes. a) High frequency barcodes for UT (untreated) arm, at all the corresponding analytic time points, are highlighted within the entire BC frequency distribution. High frequency barcodes show progressive increase of frequency along time. **b)** Frequencies of winner barcodes for –E2 (AI) arm at POT (pre-treatment), late dormancy (90 days from ET onset), awakenings (early progression) and TEPs (late progression) are highlighted within the entire BC frequency distribution at each time point. ***ε***+30 represents the second winning barcode, in ***ε*** carbon copy, getting more enriched in its TEP counterpart. **c)** Frequencies of winner barcodes for TAM arm at POT, dormancy entry (30 days), dormancy maintenance (60 days), awakening and TEPs. For both AI (-E2) and TAM arms, while winner BCs are present at high frequency in POT, their frequency at dormancy is variable.
