# Supplementary material for: Long-term Multimodal Recording Reveals Epigenetic Adaptation Routes in Dormant Breast Cancer Cells
Source: Cancer Discov. 2024 Mar 26;14(5):866–89. doi: 10.1158/2159-8290.CD-23-1161 (PMC11061610; doi:10.1158/2159-8290.CD-23-1161)
Supplement: Supplementary Figure S15 — Genomic analysis of breast cancer drivers and non-coding alterations in Traditiom samples [file cd-23-1161_supplementary_figure_s15_suppsf15.pdf]

Supplementary Figure S15. Genomic analysis of breast cancer drivers and non-coding alterations in Traditiom samples

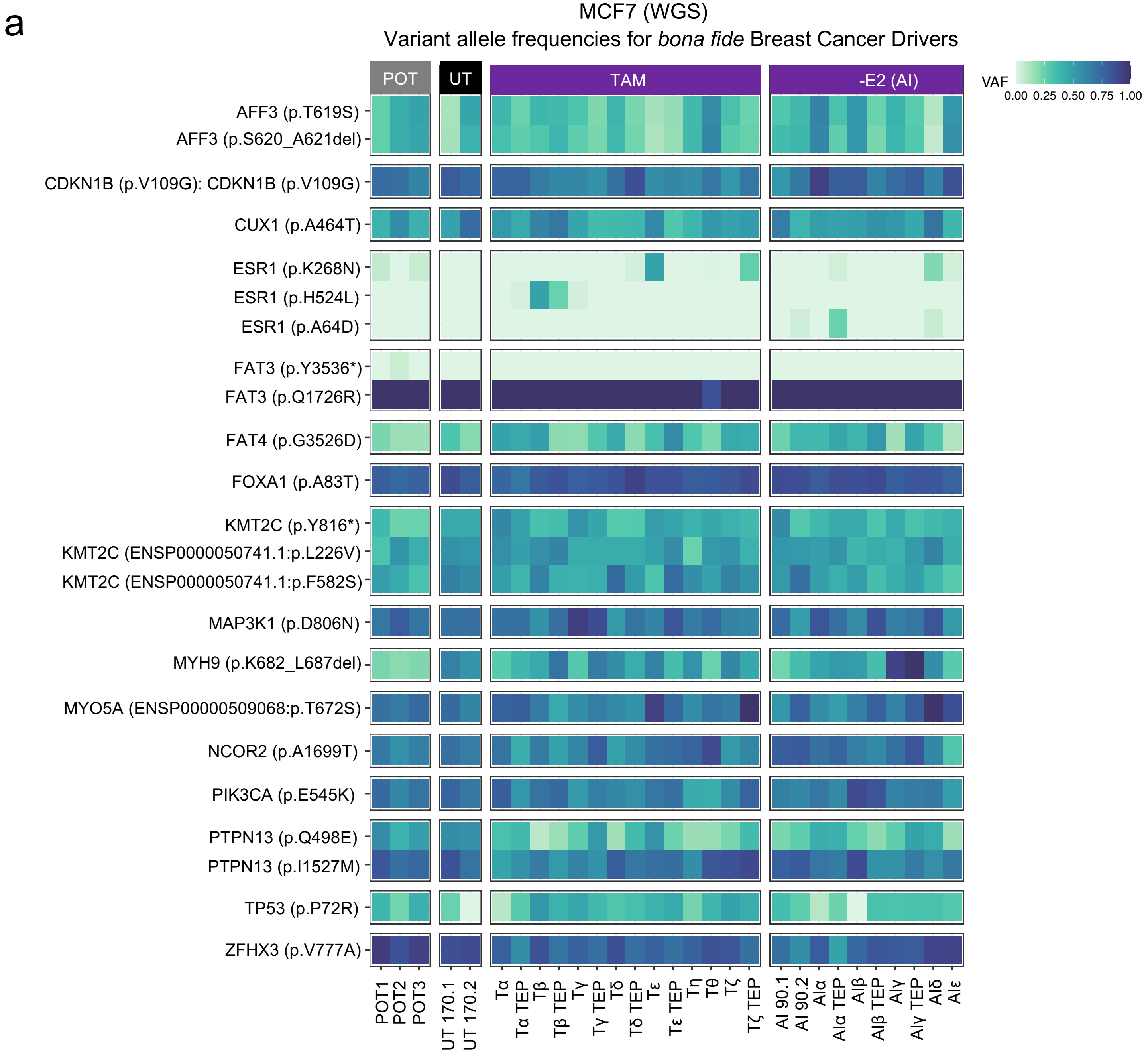

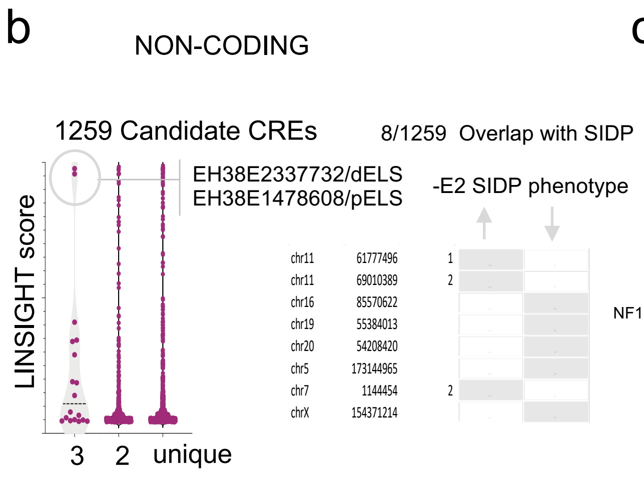

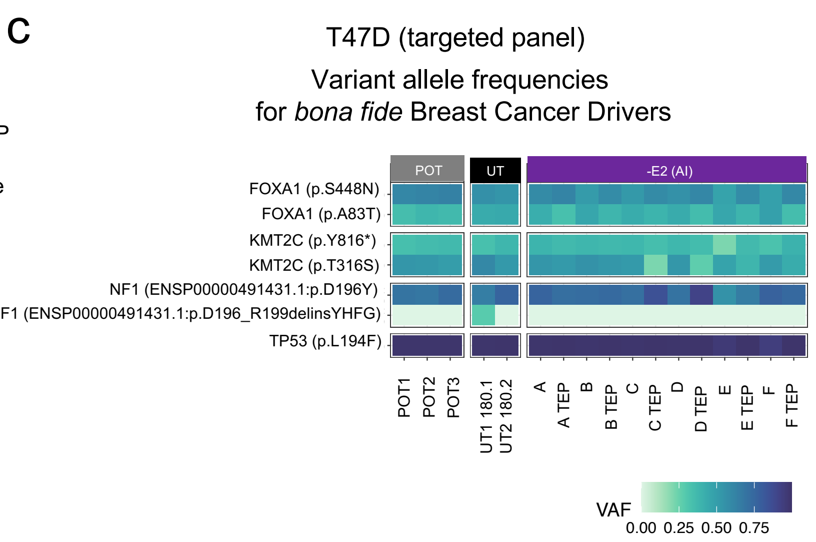

Supplementary Figure S15. TRADITIOM Genomic analysis of breast cancer drivers and non-coding alterations in Traditiom samples. a) Variant Allele Frequency (VAF) heatmap for *bona fide* breast cancer drivers derived from TRADITIOM MCF7 WGS, illustrating only variants with significant changes in VAF (Fisher's Exact test, $p < 0.01$). Data depict TRADITIOM samples from POT (pre-treatment), awakening (early progression) and TEP (late progression) of both treatment arms (TAM and –E2 (AI)) and their counterpart untreated (UT) samples cultured in parallel for 170 days. **b)** Noncoding variants were selected via Open Cravat (all SNV excluding Exome). Noncoding SNVs were filtered with the ENCODE Cis Regulatory Element function and sorted for LINSIGHT score. Noncoding SNVs called in 3 carbon copies and with a LINSIGHT score >0.4 were overlapped with our unpublished non-coding CRISPR-KRAB Screen (Repression of CRE under oestrogen deprivation, doi.org/10.1101/2022.02.15.480537). Upward pointing arrow indicates gRNA was enriched under ET treatment while downward pointing arrow indicates gRNA was depleted. Number indicates gRNA guide scoring per region. **c)** Variant Allele Frequency (VAF) heatmap for *bona fide* breast cancer drivers derived from TRADITIOM T47D custom targeted panel sequencing, illustrating only variants with significant changes in VAF (Fisher's Exact test, $p < 0.01$). Data depict TRADITIOM samples from POT (pre-treatment), awakening and TEP samples of –E2 arm and the untreated (UT) counterpart cultured in parallel for 180 days.
