# Supplementary material for: Long-term Multimodal Recording Reveals Epigenetic Adaptation Routes in Dormant Breast Cancer Cells
Source: Cancer Discov. 2024 Mar 26;14(5):866–89. doi: 10.1158/2159-8290.CD-23-1161 (PMC11061610; doi:10.1158/2159-8290.CD-23-1161)
Supplement: Supplementary Figure S16 — Morphological inspection of cells along adaptation [file cd-23-1161_supplementary_figure_s16_suppsf16.pdf]

Supplementary Figure S16. Morphological inspection of cells along adaptation

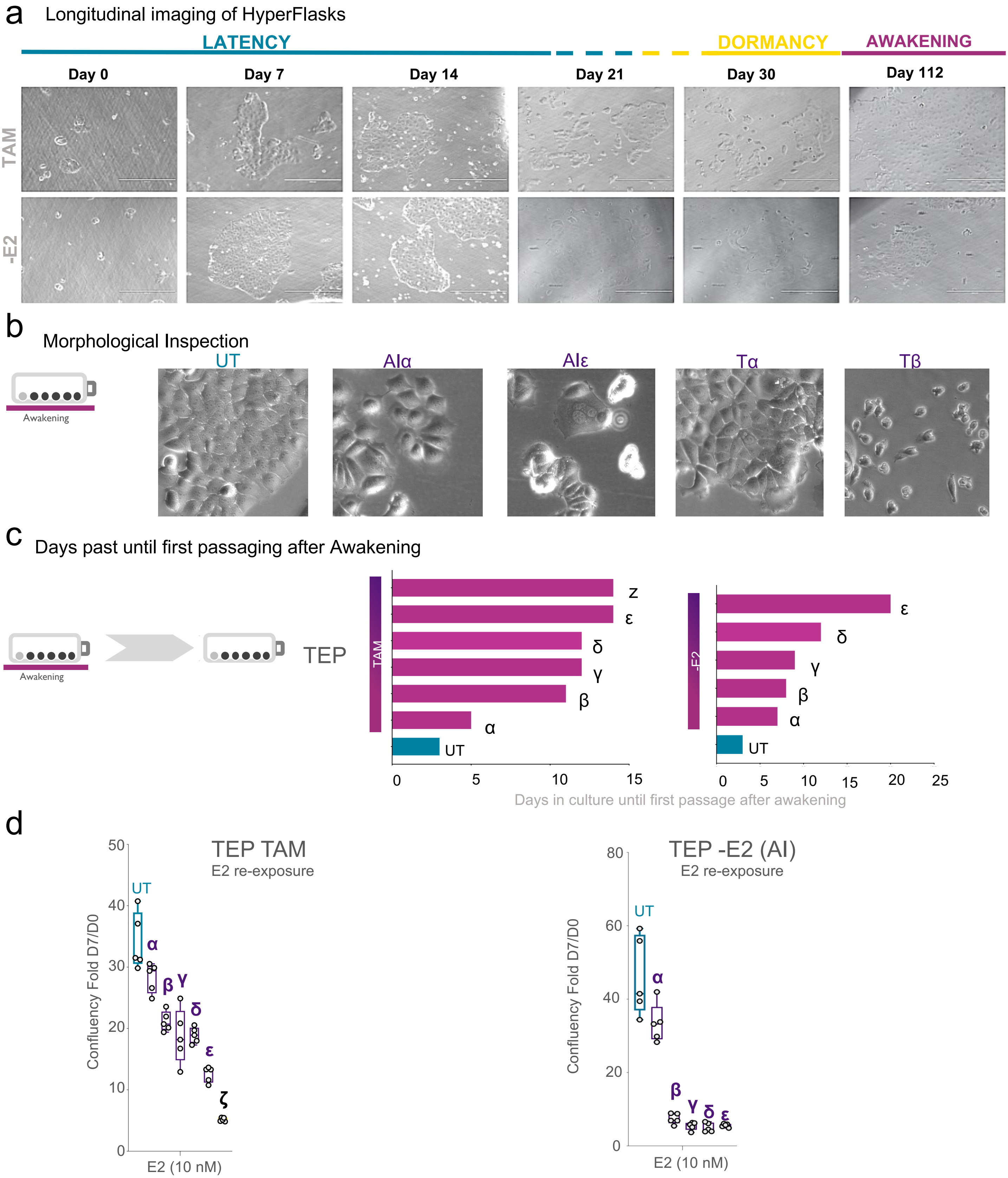

Supplementary Figure S16. Morphological inspection of cells along adaptation. a) Representative bright-field images of MCF7 –E2 (AI) and TAM HYPERFlasks (observable layers) along latency (time between treatment onset and dormancy entry), dormancy and awakening. Images were captured with EVOS Cell Imaging Systems (10X). **b)** Morphological differences between untreated (UT) and TEPs (terminal end points, late progression) were captured with EVOS Cell Imaging Systems (10X). **c)** Number of days before the first passage of awakening cells detached from HyperFlasks and replated in standard culture conditions. **d)** Growth dynamics of MCF7 TAM and –E2 (AI) TEPS when re-exposed to oestrogen (E2, 10nM). Representative graphs are shown as normalized confluency fold change upon 7 days of compound treatment (n=3).
