# Supplementary material for: Long-term Multimodal Recording Reveals Epigenetic Adaptation Routes in Dormant Breast Cancer Cells
Source: Cancer Discov. 2024 Mar 26;14(5):866–89. doi: 10.1158/2159-8290.CD-23-1161 (PMC11061610; doi:10.1158/2159-8290.CD-23-1161)
Supplement: Supplementary Figure S17 — TRADITIOM MCF7 Bulk RNA-seq [file cd-23-1161_supplementary_figure_s17_suppsf17.pdf]

Supplementary Figure S17. TRADITIOM MCF7 Bulk RNA-seq

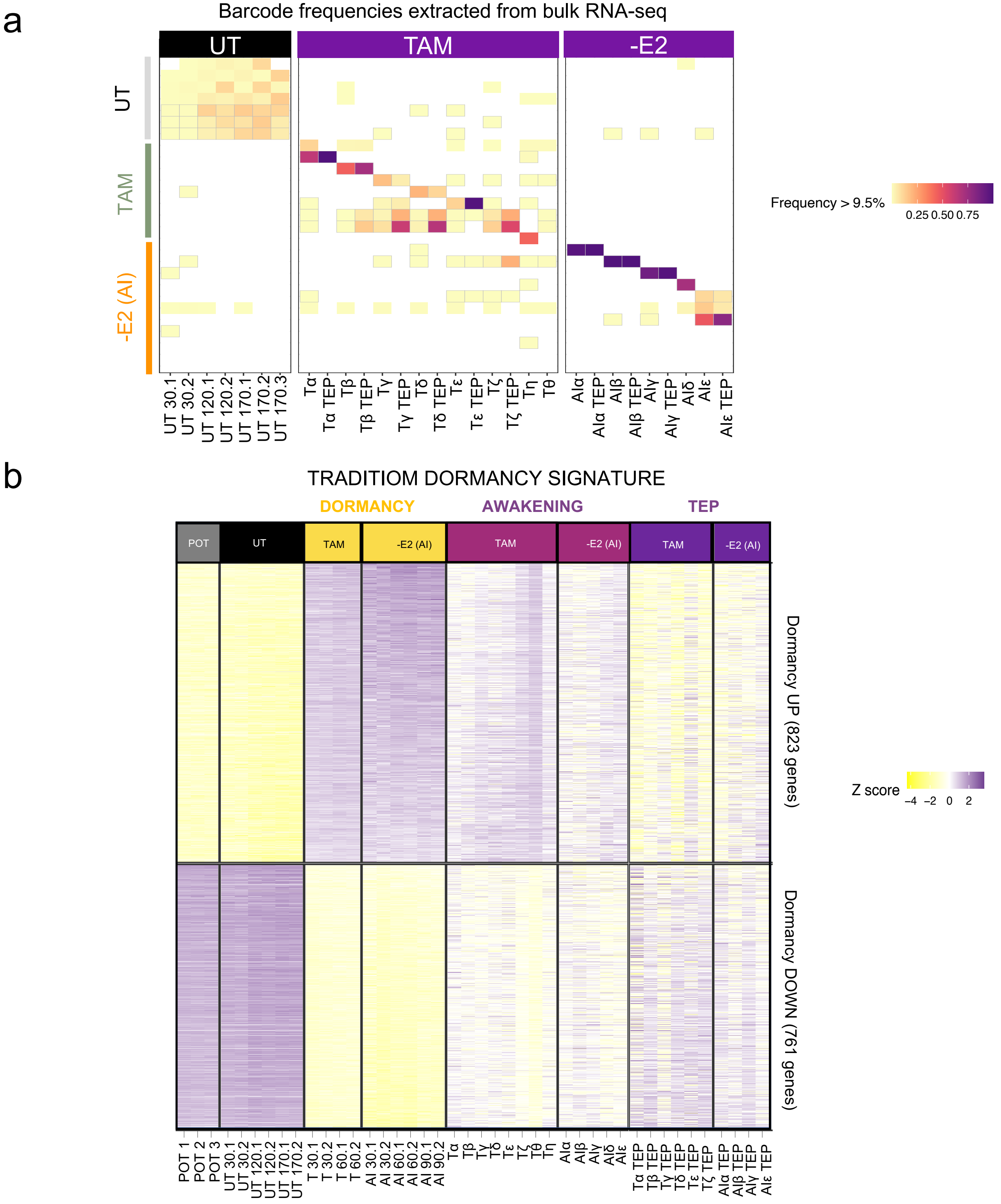

Supplementary Figure S17. TRADITIOM MCF7 Bulk RNA-seq. a) Heatmap of high frequency barcodes (BC frequency>10%) among UT (untreated) samples and TRADITIOM carbon-copies (replicates) for both TAM and –E2 (AI) arm at the time of awakening (early progression) and their corresponding TEPs (Terminal End Points, late progression) as determined by barcode calls from bulk RNA-seq data. **b)** Heatmap depicting significantly differentially expressed genes (abs(log2FoldChange)>1 & padj < 0.01) at dormancy (AI day 30, 60, 90 and TAM day 30, 60) in comparison to the POT (pre-treatment). Dormancy specific gene list was created based on consensus of DEGs using z-score distributions across T0s and all dormancy samples from replicates of both TAM and –E2 (AI) treatment (dormancy up: dormancy z-score >0 and >= 0.1-quantile for at least 6 samples, dormancy down: dormancy z-score <0 and <= 0.9-quantile for at least 6 samples). The heatmap is further extended across UT (day 30, 120 and 170), awakening and TEPs of TAM and-E2 (AI) samples to report corresponding changes in transcriptional profile for rest of the samples.
