# Supplementary material for: Long-term Multimodal Recording Reveals Epigenetic Adaptation Routes in Dormant Breast Cancer Cells
Source: Cancer Discov. 2024 Mar 26;14(5):866–89. doi: 10.1158/2159-8290.CD-23-1161 (PMC11061610; doi:10.1158/2159-8290.CD-23-1161)
Supplement: Supplementary Figure S18 — Traditiom LSC barcode statistics at single cell level and quality checks [file cd-23-1161_supplementary_figure_s18_suppsf18.pdf]

Supplementary Figure S18. TRADITIOM LSC barcode statistics at single cell level and quality checks

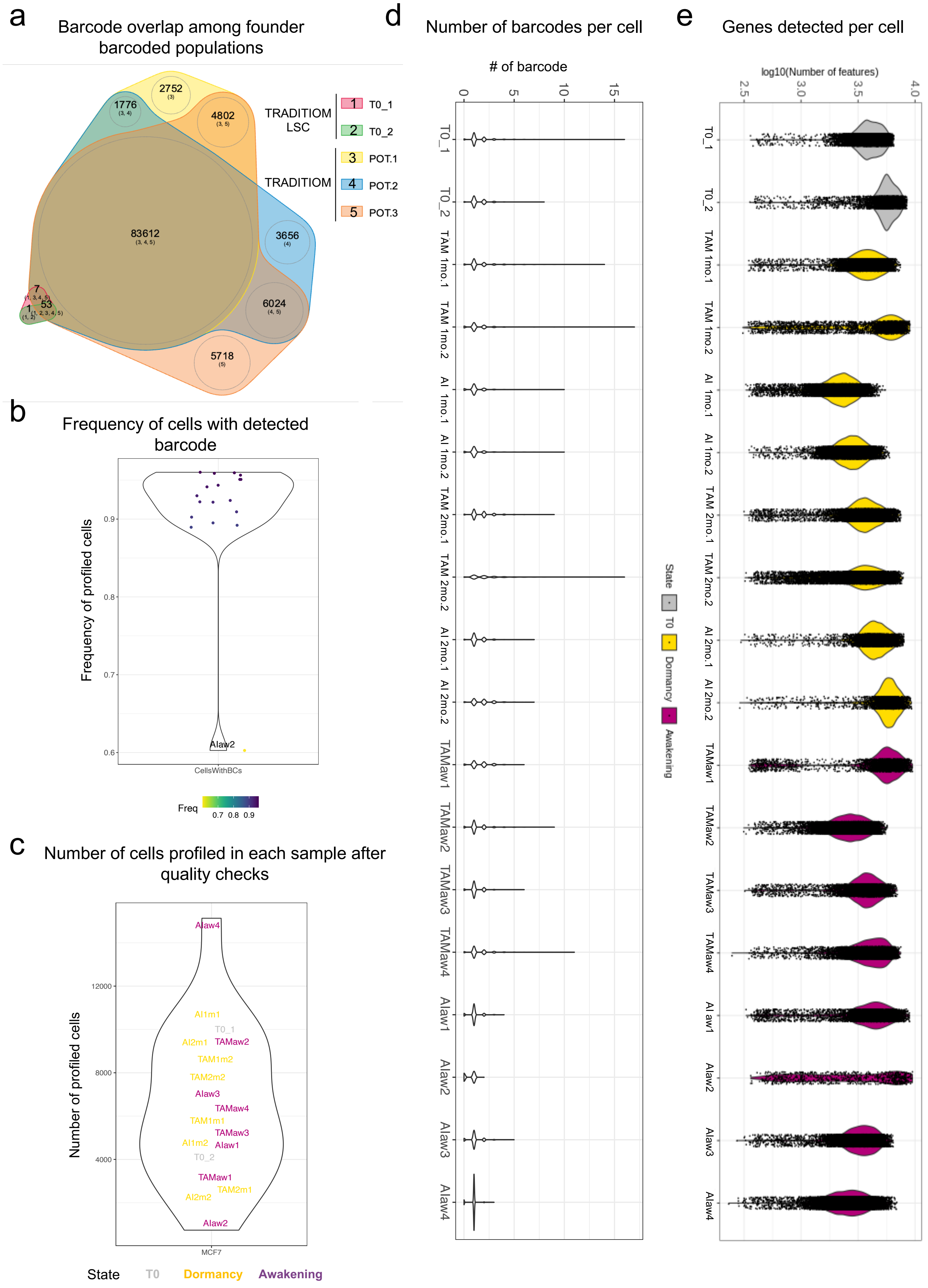

Supplementary Figure S18. TRADITIOM LSC barcode statistics at single cell level and quality checks. a) Barcode overlap between MCF7 TRADITIOM POTs (pre-treatment) (100K barcodes) and MCF7 TRADITIOM Live Single Cell (LSC) T0 (Time zero) samples (100 barcodes subsampled from TRADITIOM POT). **b)** Frequency of cells with detected barcodes. Barcodes could be detected in more than 85% of the cells for all the samples other than AIaw2 (-E2 arm, awakening sample 2). **c)** Number of cells profiled in each MCF7 TRADITIOM LSC (live single cell) sample after quality checks. **d)** Number of detected barcodes per cell along TRADITIOM LSC for MCF7 cells. **e)** Number of genes detected per cell in MCF7 TRADITIOM dataset.
