# Supplementary material for: Long-term Multimodal Recording Reveals Epigenetic Adaptation Routes in Dormant Breast Cancer Cells
Source: Cancer Discov. 2024 Mar 26;14(5):866–89. doi: 10.1158/2159-8290.CD-23-1161 (PMC11061610; doi:10.1158/2159-8290.CD-23-1161)
Supplement: Supplementary Figure S20 — TRADITIOM MCF7 LSC barcode analysis [file cd-23-1161_supplementary_figure_s20_suppsf20.pdf]

# Supplementary Figure S20. TRADITIOM MCF7 LSC barcode analysis

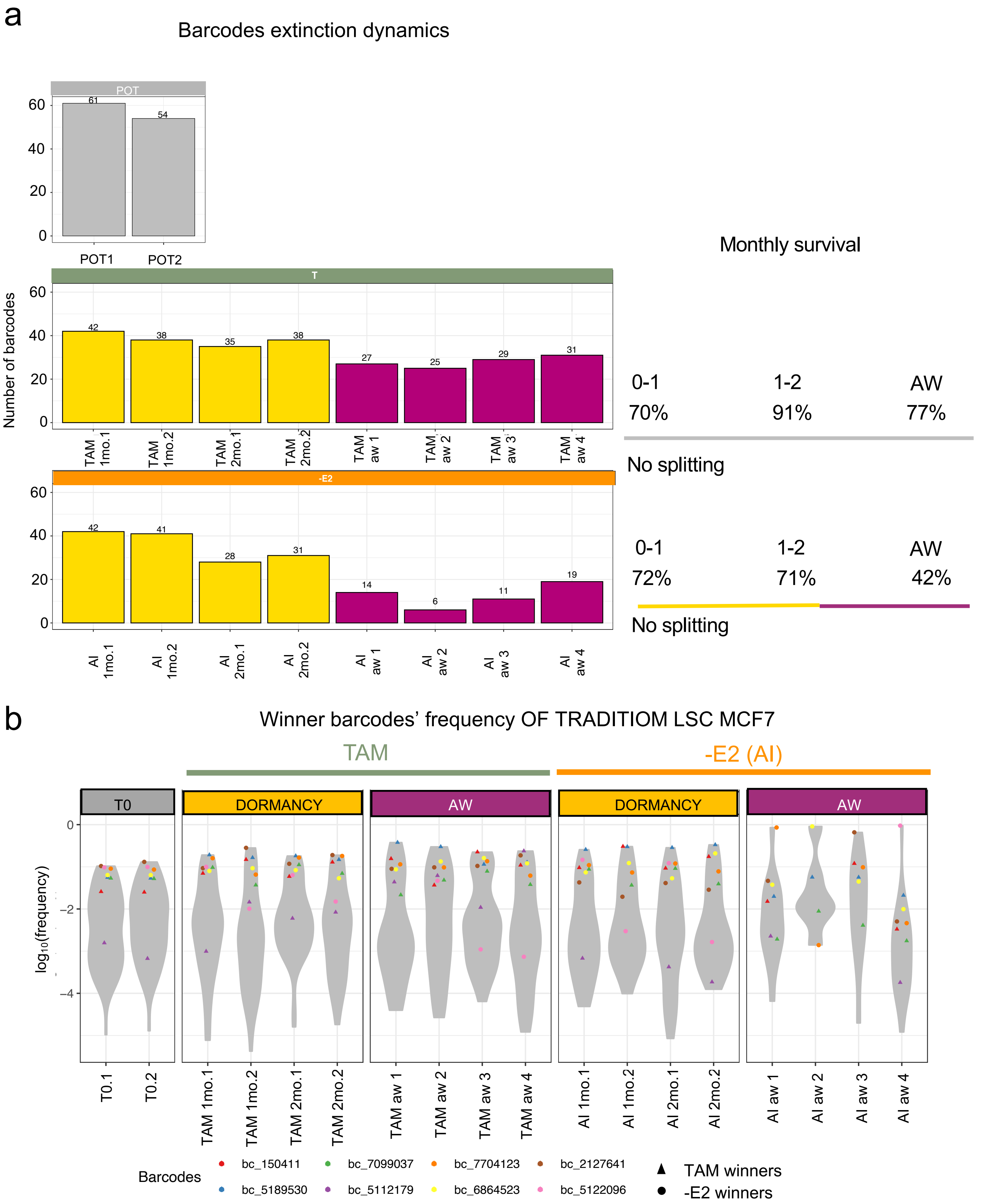


**Supplementary Figure S20. TRADITIOM MCF7 LSC barcode analysis. a)** Number of initial barcodes at POT (pre-treatment) and number of surviving barcodes at each indicated time point (dormancy and awakening), are listed for TAM and –E2 (AI) arms of TRADITIOM LSC (live single cell) MCF7. A summary of the percentages of surviving barcodes along the collection points is shown on the right side of the panel. **b)** Frequencies of awakening carbon copies' winner barcodes (triangle: TAM arm winners, circle: -E2 (AI) arm winners) are highlighted on the barcode frequency distribution plots from T0 (onset of treatment) to awakening (early progression).
