# Supplementary material for: Long-term Multimodal Recording Reveals Epigenetic Adaptation Routes in Dormant Breast Cancer Cells
Source: Cancer Discov. 2024 Mar 26;14(5):866–89. doi: 10.1158/2159-8290.CD-23-1161 (PMC11061610; doi:10.1158/2159-8290.CD-23-1161)
Supplement: Supplementary Figure S21 — TRADITIOM MCF7 LSC of the TAM arm [file cd-23-1161_supplementary_figure_s21_suppsf21.pdf]

Supplementary Figure S21. TRADITIOM MCF7 LSC barcode analysis and clustering of the TAM arm

a

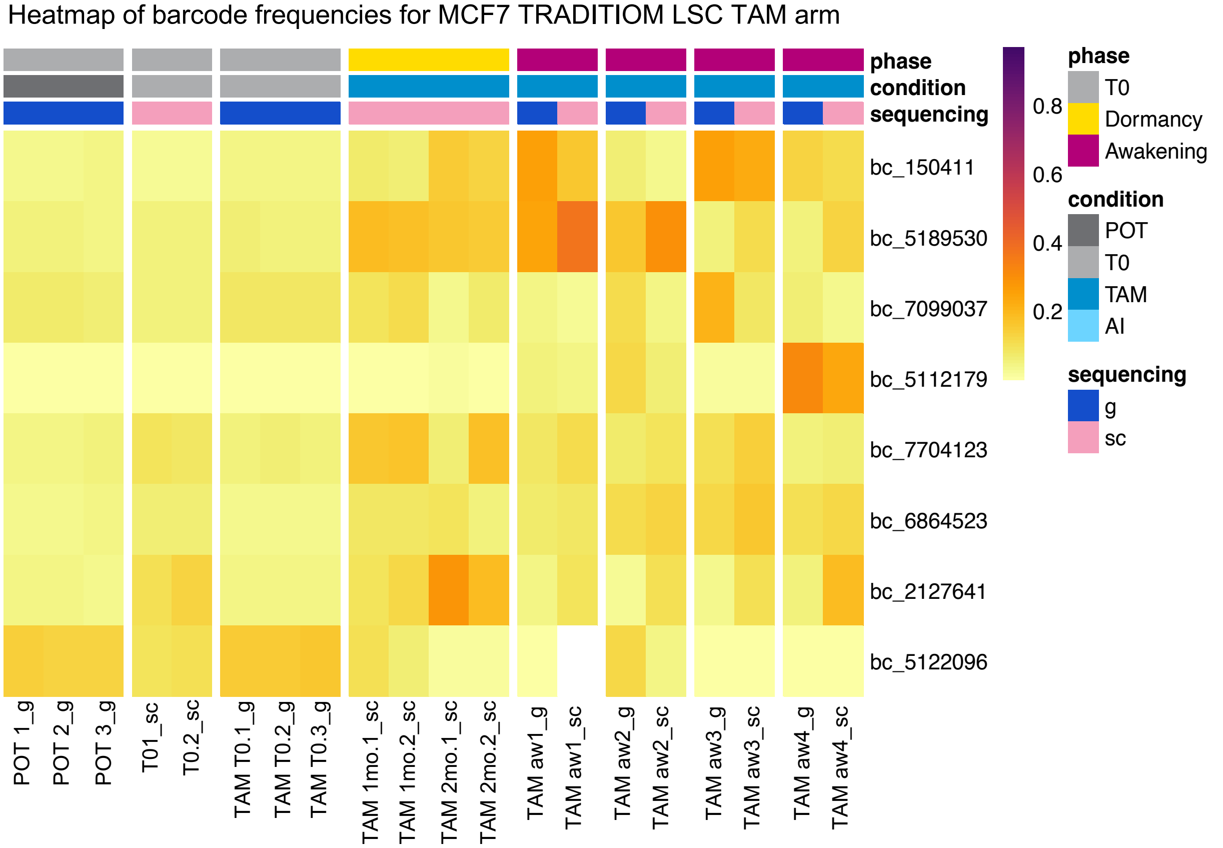

b

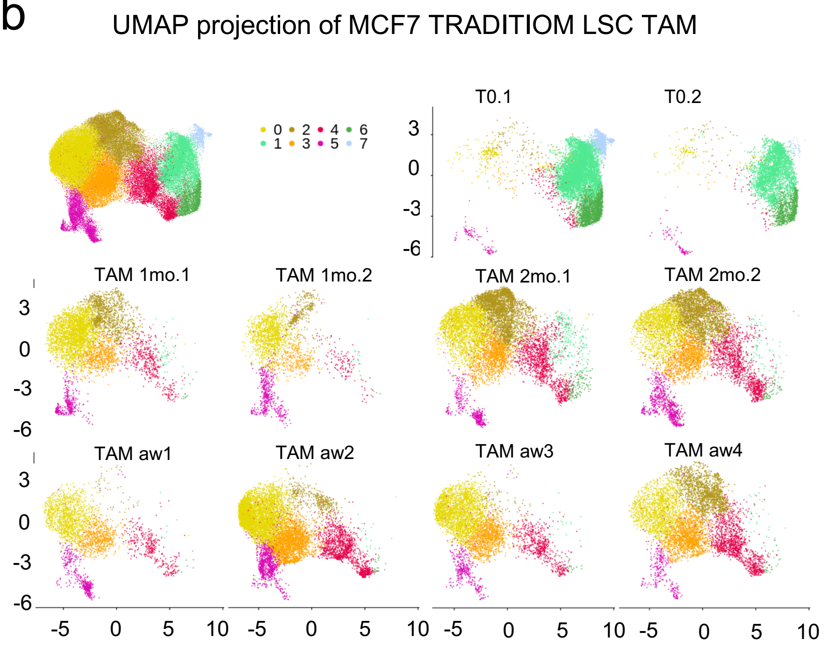

c

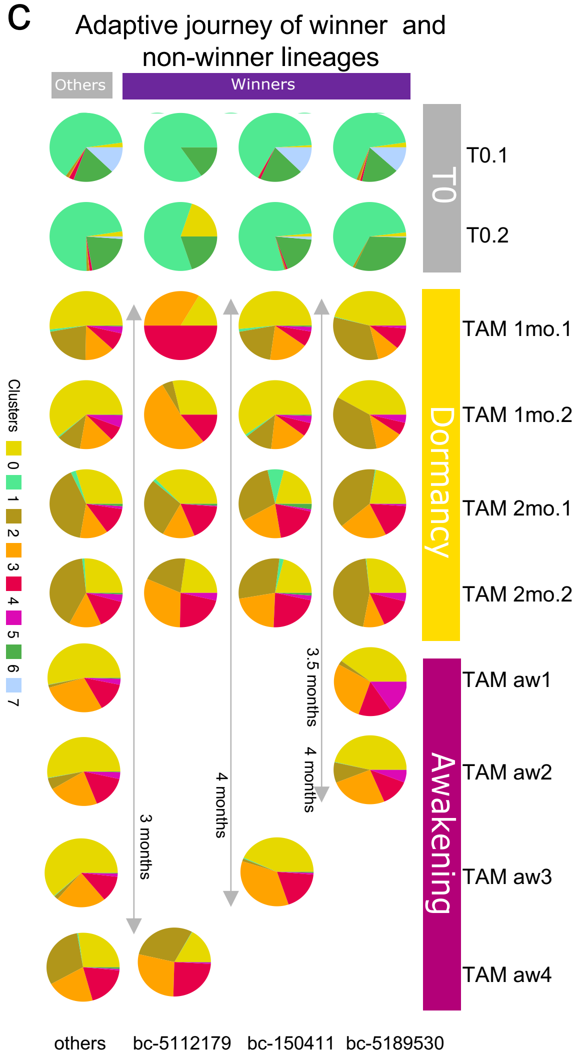

Supplementary Figure S21. TRADITIOM MCF7 LSC barcode analysis and clustering of the TAM arm. **a)** Heatmap of high frequency barcodes of MCF7 TRADITIOM LSC (live single cell) carbon copies (replicates) for TAM arm at the time of awakening from the start of the experiment (T0) including early (1 month, 1mo) and late dormancy (2 months, 2mo) stages. Barcodes were derived from either genomic barcode sequencing (g) or scRNA-seq (sc). **b)** UMAP projections of MCF7 TRADITIOM LSC TAM arm carbon copies from T0 to awakening. **c)** Adaptive journeys of winner and non-winner lineages (others) of each TRADITIOM LSC TAM arm awakening sample from T0 to awakening. Pie charts depict the occupancy of miscellaneous UMAP clusters for each lineage.
