# Supplementary material for: Long-term Multimodal Recording Reveals Epigenetic Adaptation Routes in Dormant Breast Cancer Cells
Source: Cancer Discov. 2024 Mar 26;14(5):866–89. doi: 10.1158/2159-8290.CD-23-1161 (PMC11061610; doi:10.1158/2159-8290.CD-23-1161)
Supplement: Supplementary Figure S22 — TRADITIOM LSC Cell cycle states [file cd-23-1161_supplementary_figure_s22_suppsf22.pdf]

Supplementary Figure S22. TRADITIOM LSC Cell cycle states

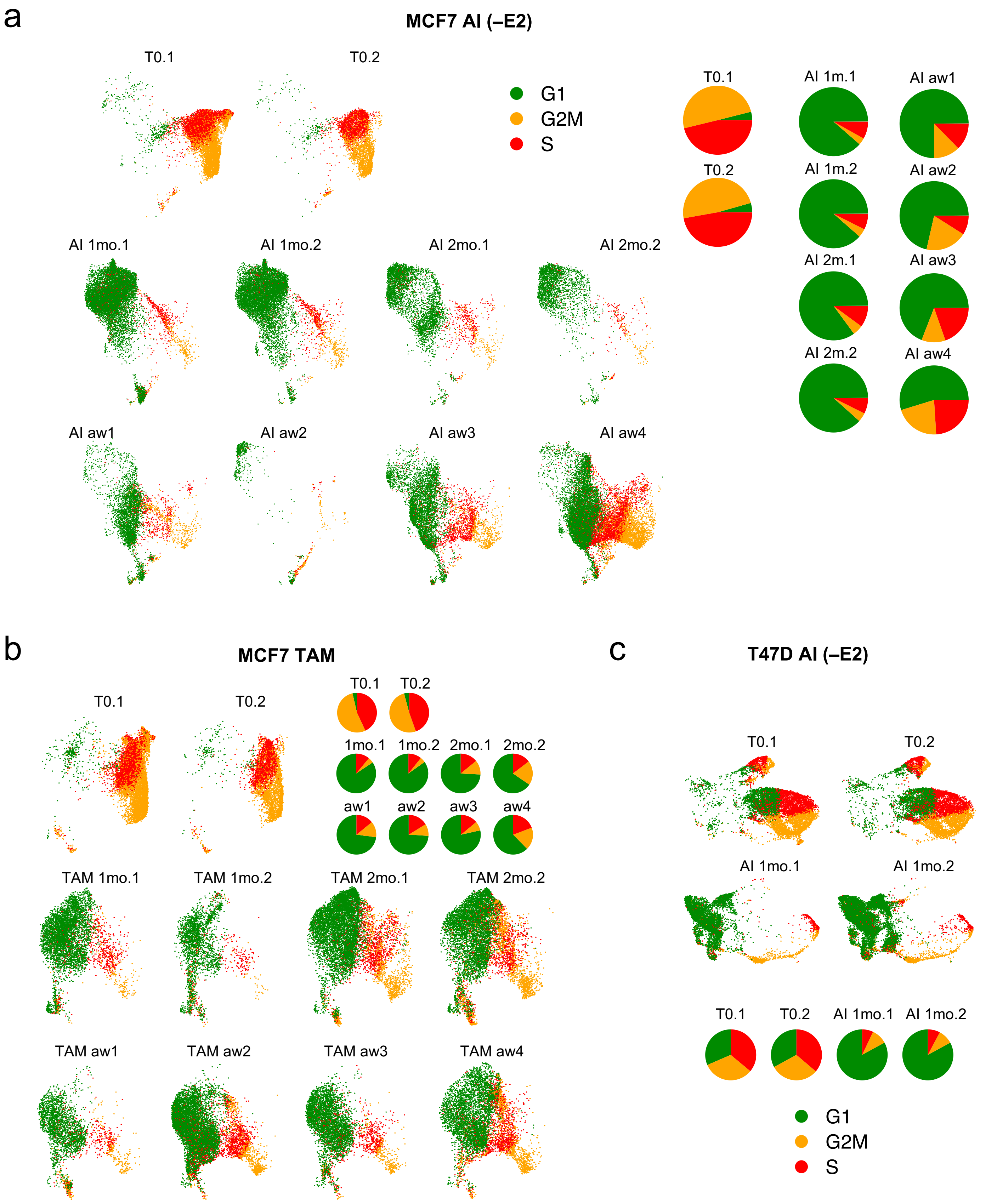

Supplementary Figure S22. TRADITIOM LSC Cell cycle states. UMAP projections of TRADITIOM LSC (live single cell) carbon copies (replicates) for MCF7 AI (-E2) arm **(a)** and TAM arm **(b)** from T0 to awakening and T47D AI (–E2) **(c)** from T0 to dormancy (1 month, 1mo) depicting cell cycle states (G1: green, S: red and G2M: orange).
