# Supplementary material for: Long-term Multimodal Recording Reveals Epigenetic Adaptation Routes in Dormant Breast Cancer Cells
Source: Cancer Discov. 2024 Mar 26;14(5):866–89. doi: 10.1158/2159-8290.CD-23-1161 (PMC11061610; doi:10.1158/2159-8290.CD-23-1161)
Supplement: Supplementary Figure S23 — High frequency barcode occupancy of scRNA-seq clusters [file cd-23-1161_supplementary_figure_s23_suppsf23.pdf]

Supplementary Figure S23. High frequency barcode occupancy of scRNA-seq clusters

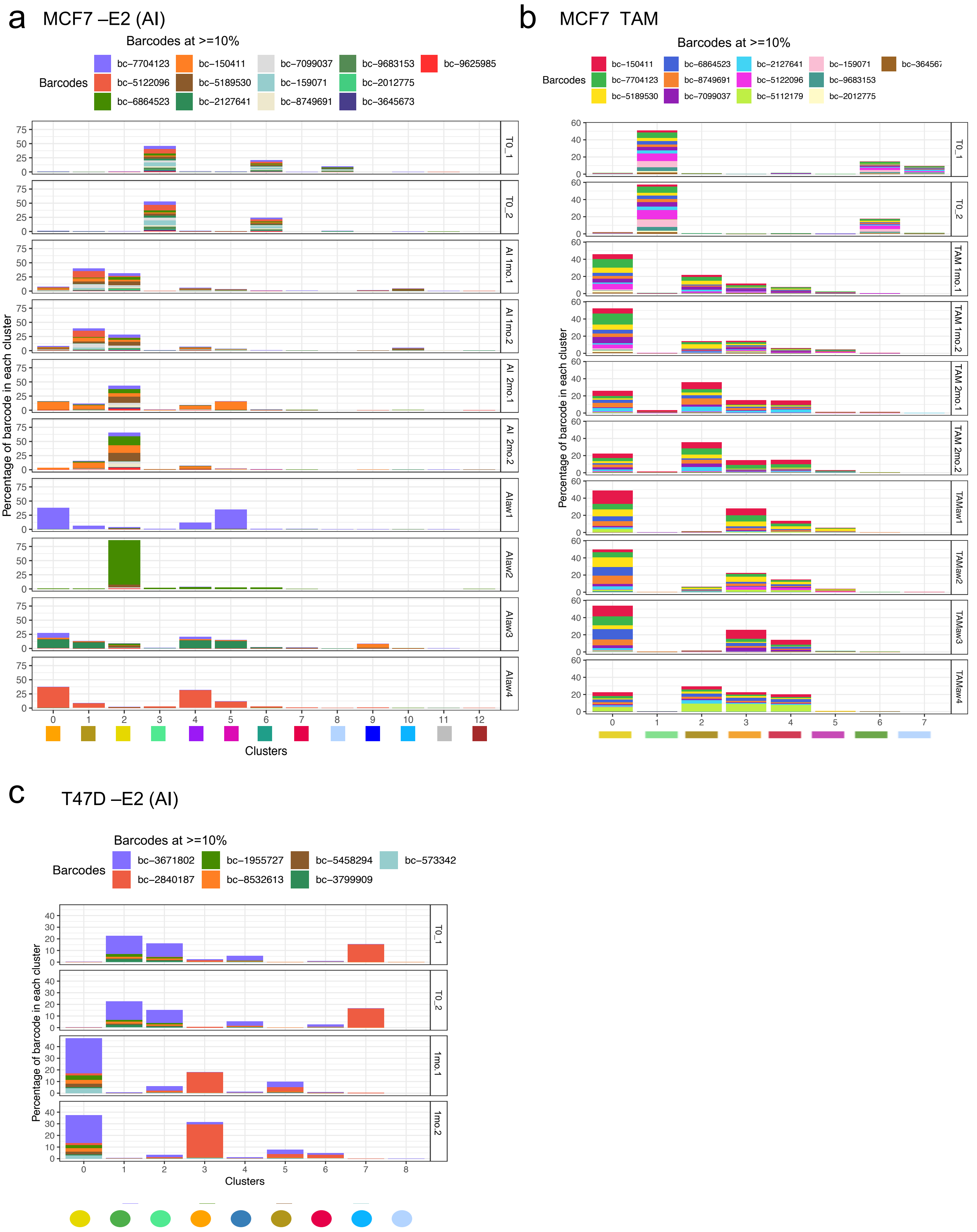

Supplementary Figure S23. High frequency barcode occupancy of scRNA-seq clusters. Bar plots depict the percentage of each lineage (barcode frequency >10%) in distinct UMAP clusters for TRADITIOM LSC (live single cell) MCF7 –E2 (AI) **(a)**, MCF7 TAM **(b)** from T0, early dormancy (1month, 1mo), late dormancy (2month, 2mo) to awakening and T47D –E2 (AI) **(c)** from T0 to dormancy (1month, 1mo).
