# Supplementary material for: Long-term Multimodal Recording Reveals Epigenetic Adaptation Routes in Dormant Breast Cancer Cells
Source: Cancer Discov. 2024 Mar 26;14(5):866–89. doi: 10.1158/2159-8290.CD-23-1161 (PMC11061610; doi:10.1158/2159-8290.CD-23-1161)
Supplement: Supplementary Figure S24 — Cell cycle regression of TRADITIOM MCF7 LSC for AI (–E2) arm [file cd-23-1161_supplementary_figure_s24_suppsf24.pdf]

Supplementary Figure S24. Cell cycle regression of TRADITIOM MCF7 LSC for AI (–E2) arm

a UMAP clustering of cell cycle regressed samples

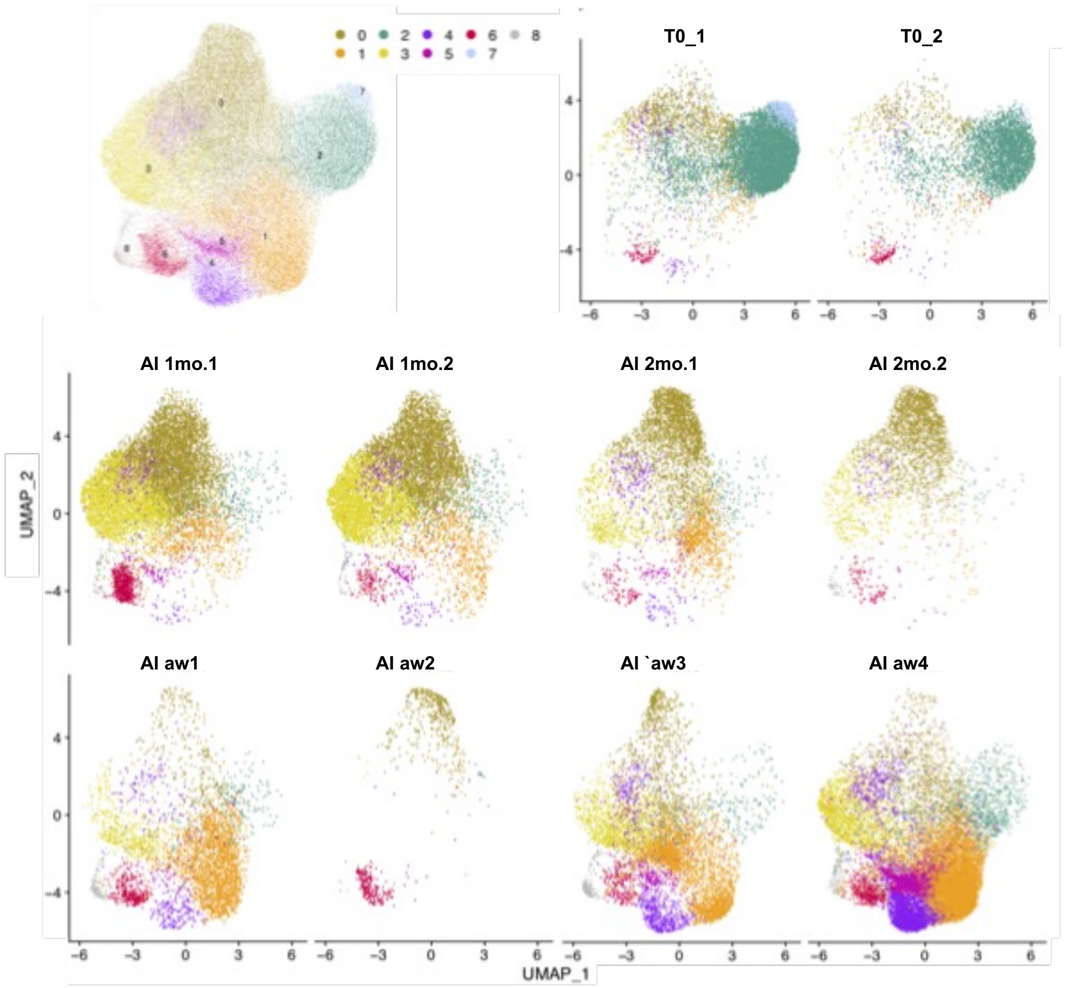

b Adaptive journey of winner and non-winner lineages

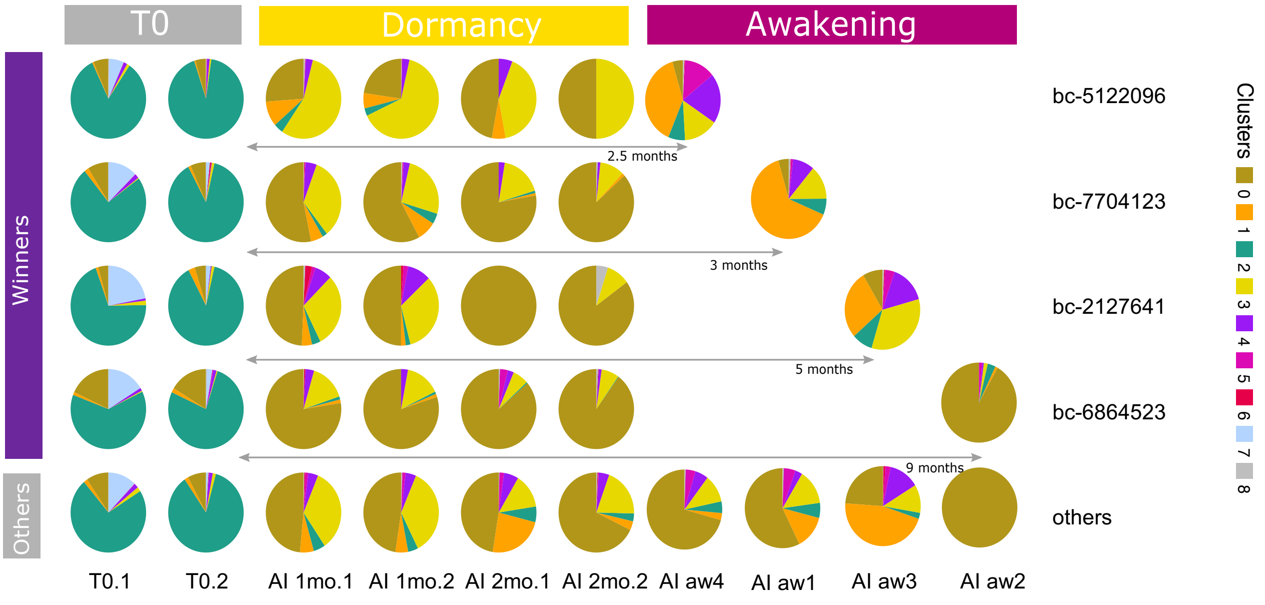

Supplementary Figure S24. Cell cycle regression of TRADITIOM MCF7 LSC for AI (–E2) arm.
a) UMAP projections for MCF7 TRADITIOM LSC (live single cell) –E2 (AI) arm (T0, early dormancy (1month, 1mo), late dormancy (2months, 2mo) and awakening) including cell cycle regression to correct for the putative confounding factor of changes in cell cycle dynamics during dormancy and awakening. **b)** Adaptive journeys of winner lineages of each TRADITIOM LSC awakening sample along with the non-winner lineages (others) profiled from T0 to awakening. Pie charts depict the cell cycle-regressed lineage occupancy of miscellaneous UMAP clusters. The approximate awakening time of each carbon copy (replicate) is shown with arrows.
