# Supplementary material for: Long-term Multimodal Recording Reveals Epigenetic Adaptation Routes in Dormant Breast Cancer Cells
Source: Cancer Discov. 2024 Mar 26;14(5):866–89. doi: 10.1158/2159-8290.CD-23-1161 (PMC11061610; doi:10.1158/2159-8290.CD-23-1161)
Supplement: Supplementary Figure S25 — Failed awakening characterization [file cd-23-1161_supplementary_figure_s25_suppsf25.pdf]

Supplementary Figure S25. Failed Awakening characterization

a TRADITIOM Live: awakening topography (12 cc)

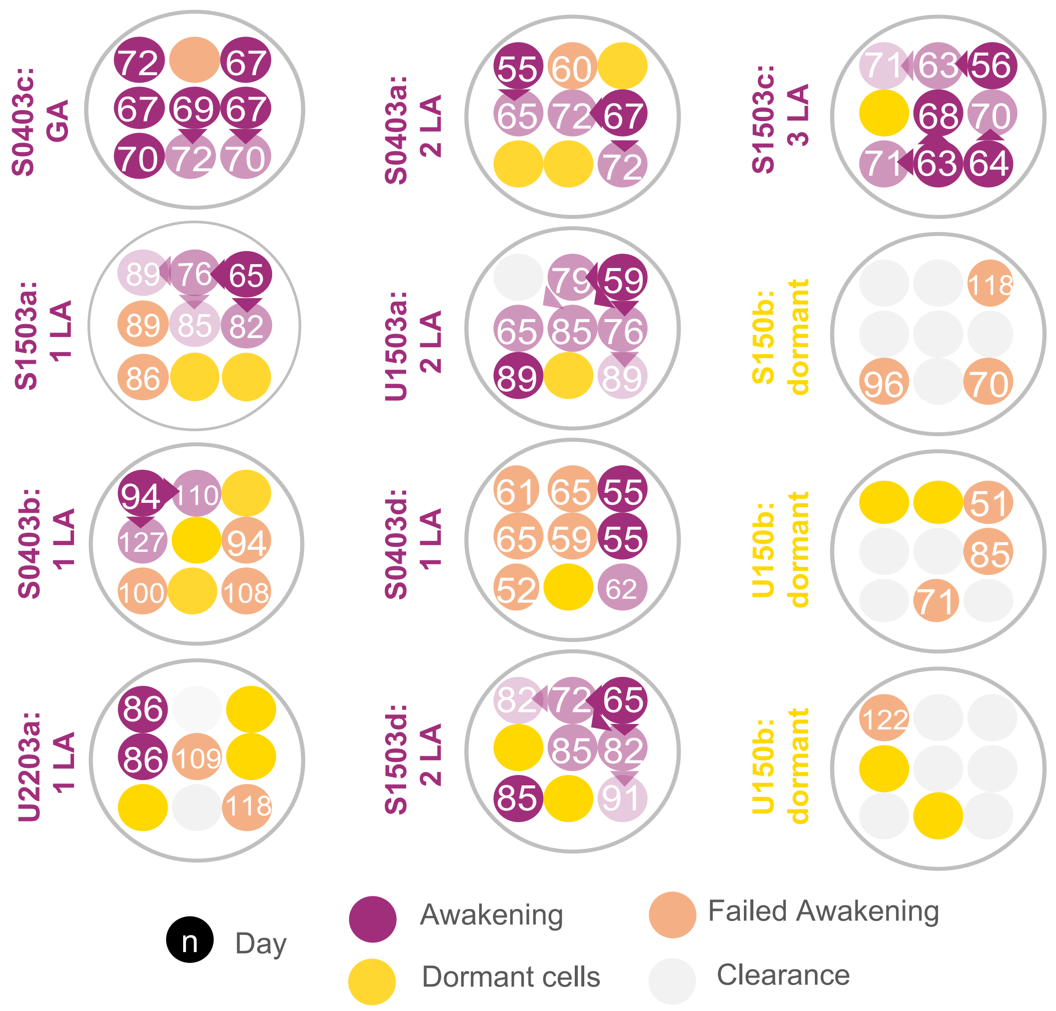

b UMAP projections of cycling and non-cycling winner barcodes vs others

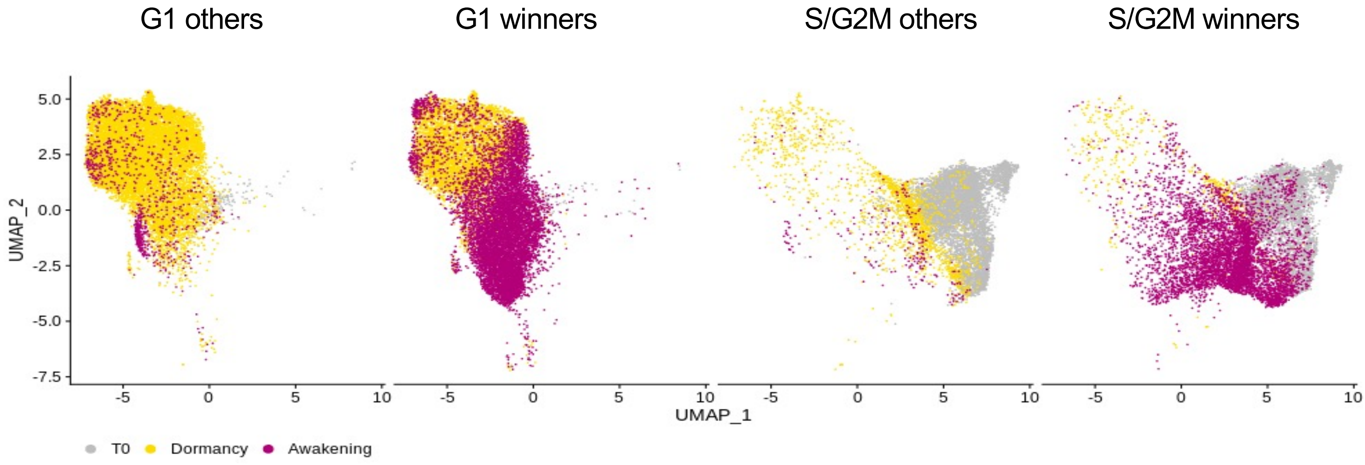

Supplementary Figure S25. Failed awakening characterization. a) TRADITIOM-Live awakening topography, detailed analysis. Cells were seeded in 48-well format, exposed to oestrogen deprivation (-E2) and imaged (9 scanning windows *per* well) to monitor awakening dynamics (awakening=wells reaching a confluency of 50%). GA: global awakening, LA: localized awakening. Arrows indicate the expansion of a growing colony in the adjacent scanning window. **b)** UMAP projections of cycling (S/G2M) and non-cycling (G1) winner vs non-winner lineages (others) at dormancy (yellow) and awakening (magenta).
