# Supplementary material for: Long-term Multimodal Recording Reveals Epigenetic Adaptation Routes in Dormant Breast Cancer Cells
Source: Cancer Discov. 2024 Mar 26;14(5):866–89. doi: 10.1158/2159-8290.CD-23-1161 (PMC11061610; doi:10.1158/2159-8290.CD-23-1161)
Supplement: Supplementary Figure S7 — Differential expression profiling of tumour biopsies from spatially resolved CK+ segments of patients 1-3 (rare cohort treated with long-term ET until progression) [file cd-23-1161_supplementary_figure_s7_suppsf7.pdf]

Supplementary Figure S7. Differential expression profiling of tumour biopsies from spatially resolved CK+ segments of Patients 1-3

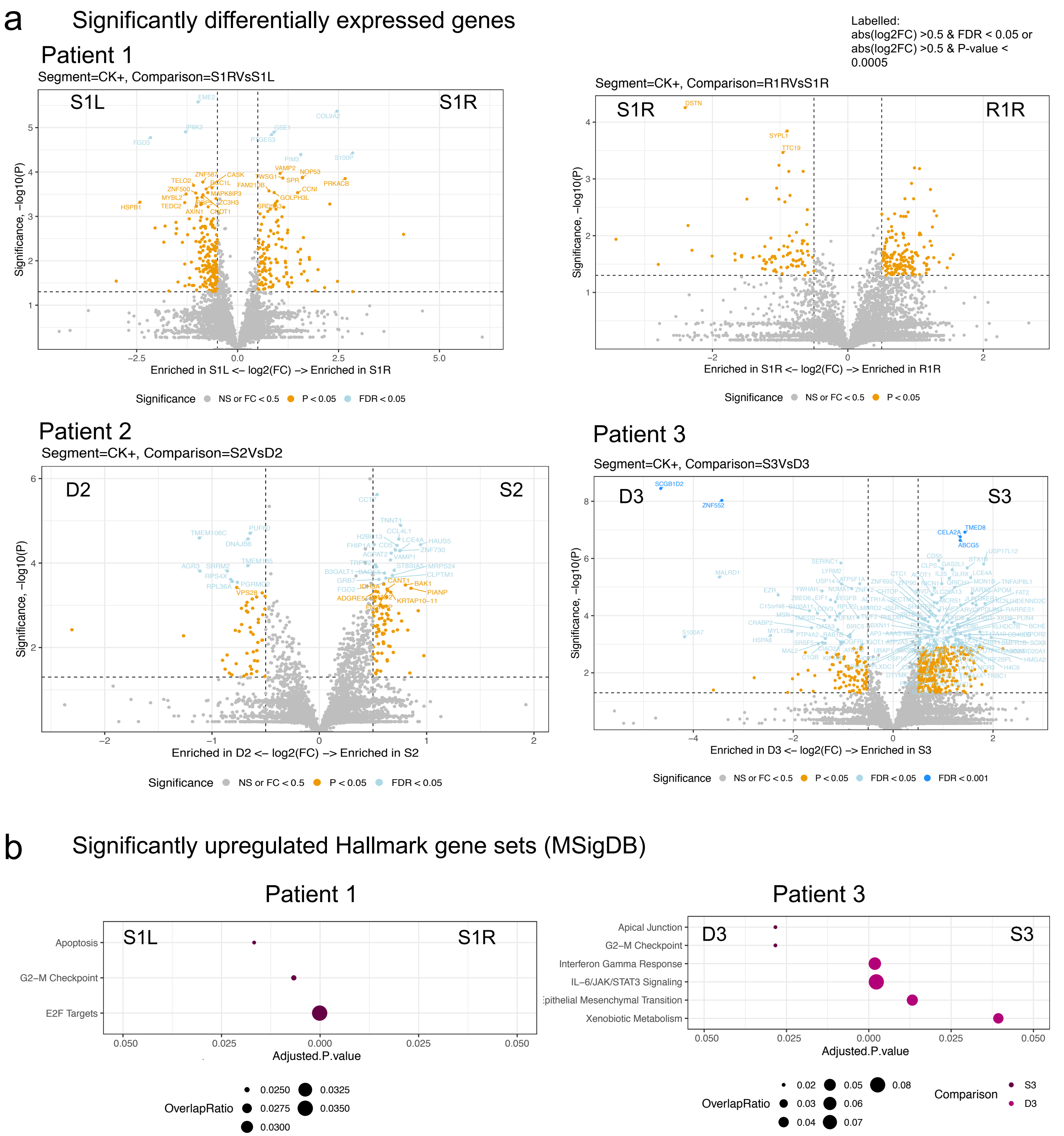

Supplementary Figure S7. Differential expression profiling of tumour biopsies from spatially resolved CK+ segments of patients 1-3. a) Significantly differentially expressed genes (DEGs) in each comparison were identified based on mean expression level from different ROIs. DEGs were labelled (abs(log2FC) >0.5 & FDR < 0.05 or abs(log2FC) >0.5 & P-value < 0.0005) and colour-coded according to the figure legend of each Volcano plot. **b)** The dot plots report significantly upregulated gene sets (MSigDB Hallmark 2020) for DEGs identified in each comparison. Position corresponds to the adjusted p-value on x axis, size indicates the overlap ratio with the corresponding gene set and colour-based on biopsy. For patient 1, the dots to left of 0 report gene sets enriched in the left breast from surgery biopsy (S1L) and to the right of 0 report gene sets enriched in the right breast (S1R). Similarly, for patient 3 dots to the left of 0 correspond to gene sets enriched in diagnostic biopsy (D3) and to the right are those that were enriched in surgical biopsy (S3).
