# Supplementary material for: Long-term Multimodal Recording Reveals Epigenetic Adaptation Routes in Dormant Breast Cancer Cells
Source: Cancer Discov. 2024 Mar 26;14(5):866–89. doi: 10.1158/2159-8290.CD-23-1161 (PMC11061610; doi:10.1158/2159-8290.CD-23-1161)
Supplement: Supplementary Figure S19 — TRADITIOM LSC awakening dynamics [file cd-23-1161_supplementary_figure_s19_suppsf19.pdf]

Supplementary Figure S19. TRADITIOM LSC awakening dynamics

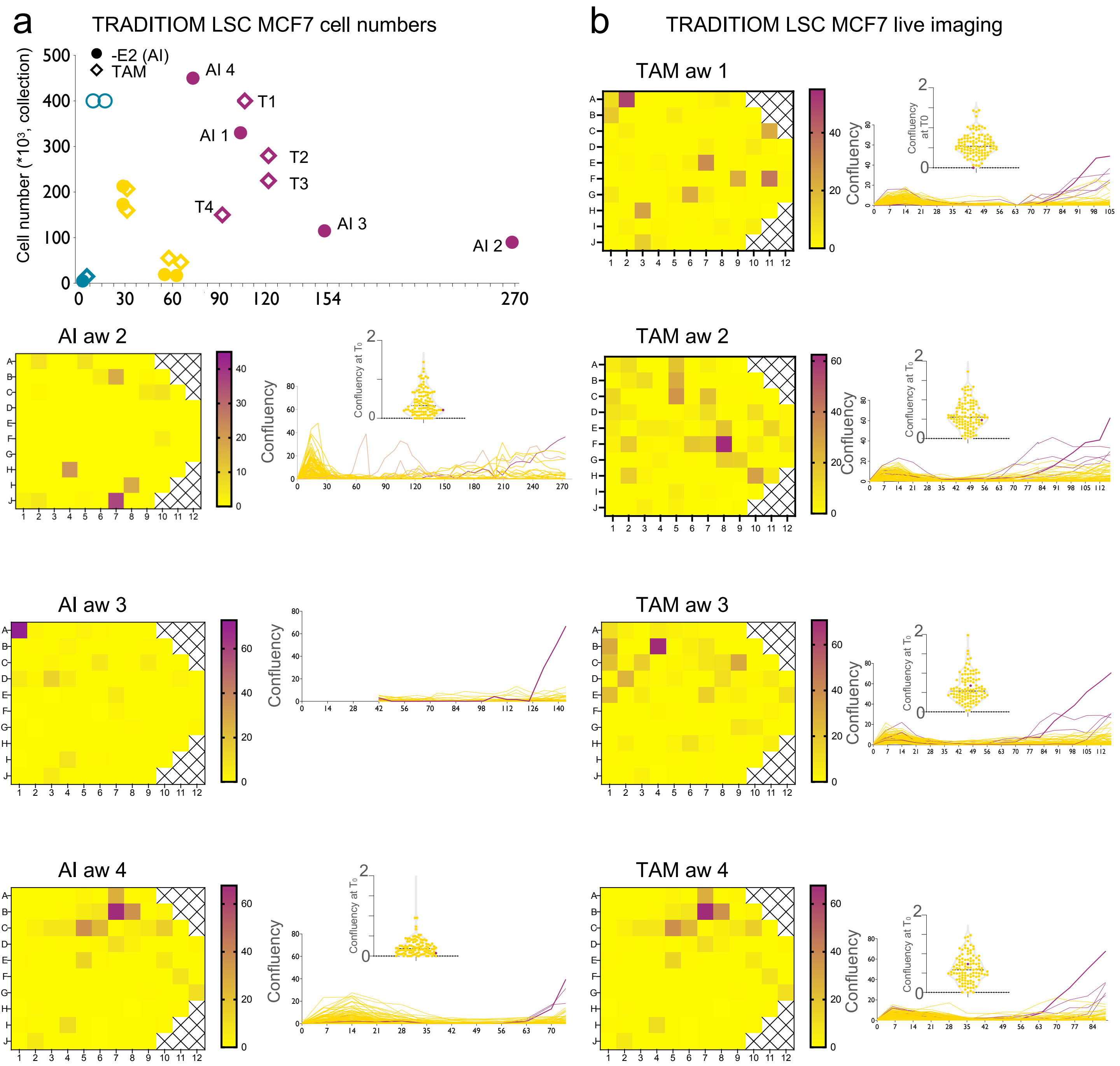

Supplementary Figure S19. TRADITIOM LSC awakening dynamics. a) Cell counts for TRADITIOM LSC (live single cell) MCF7 flasks for –E2 (AI) (circle) and TAM (diamond) arms at their respective time of collection for scRNA-seq analysis (teal: latency, yellow: dormancy, magenta: awakening). **b)** Proliferation dynamics of TRADITIOM LSC samples TAM aw1-4 (awakening 1-4) and –E2 (AI) aw2-4 (awakening 2-4) determined by weekly Incucyte imaging along with the local confluency heatmap at the time of collection (awakening). Magenta lines follow the growth dynamics of main awakening areas from T0 to overt expansion that represents the collection point for scRNA-seq analysis. Violin plots show the confluency of each scanning area at T0 (onset of oestrogen deprivation). The T0 confluency of awakening area is highlighted in purple.
