# Supplementary material for: Long-term Multimodal Recording Reveals Epigenetic Adaptation Routes in Dormant Breast Cancer Cells
Source: Cancer Discov. 2024 Mar 26;14(5):866–89. doi: 10.1158/2159-8290.CD-23-1161 (PMC11061610; doi:10.1158/2159-8290.CD-23-1161)
Supplement: Supplementary Figure S28 — Expression of epigenetic dormancy signature genes [file cd-23-1161_supplementary_figure_s28_suppsf28.pdf]

# Supplementary Figure S28. Expression of the epigenetic dormancy signature genes

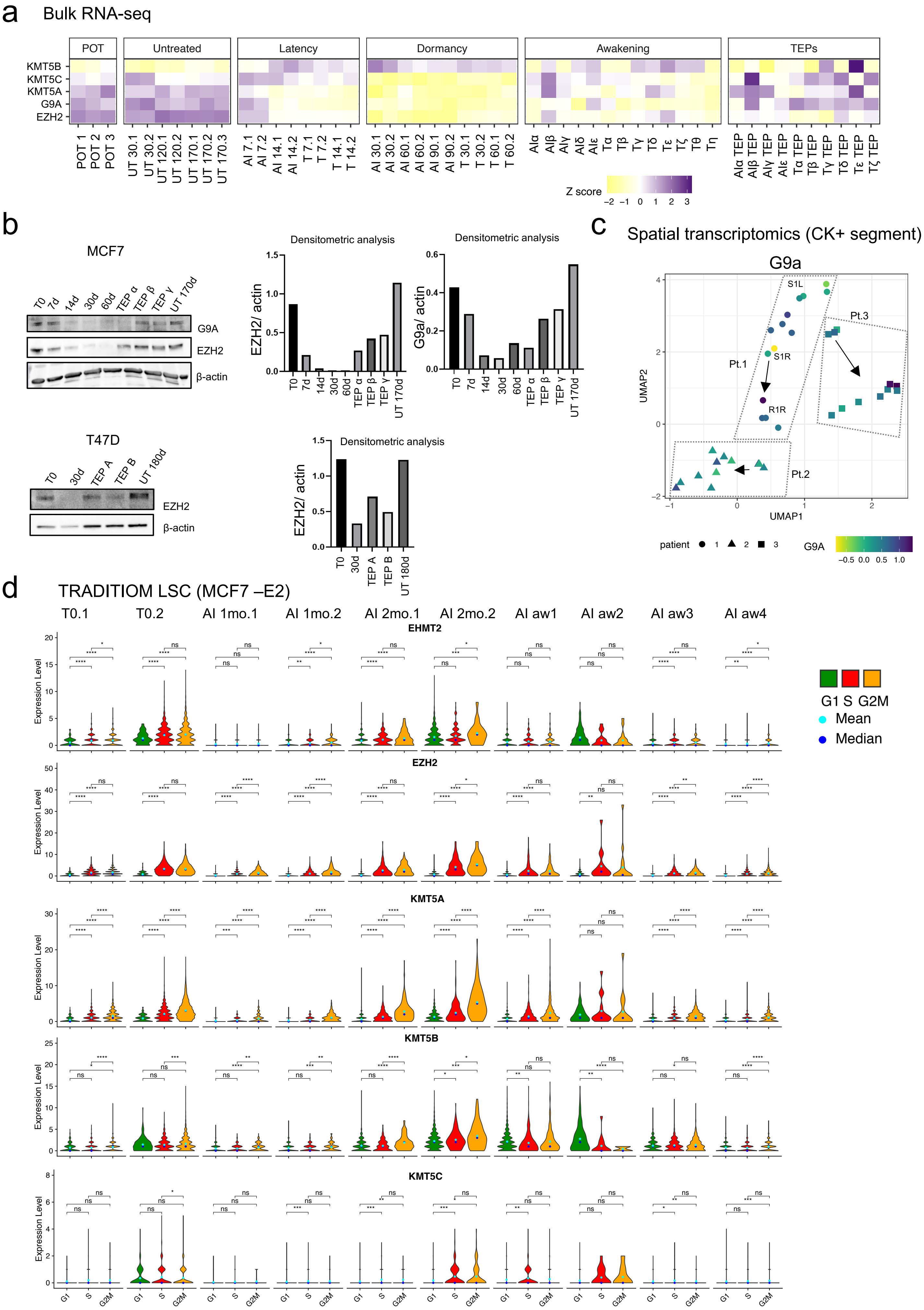

**Supplementary Figure S28. Expression of the epigenetic dormancy signature genes. a)** Heatmap depicting RNA expression levels of *KMT5A/B/C*, *G9A* and *EZH2* from bulk RNA-seq in TRADITIOM MCF7 dataset. **b)** Protein expression levels of G9A (for MCF7) and EZH2 (for MCF7 and T47D) are shown with Western blotting for TRADITIOM samples from POT (pre-treatment) to TEPs (late progression). Densitometric analyses of the bands (normalized to beta-actin) are shown as bar plots. **c)** UMAP projection of spatial transcriptomic data for G9A expression from patients 1-3 are shown for CK+ segment. **d)** Violin plots depicting scRNA-seq expression levels of *EZH2*, *G9A (EHMT2)*, *KMT5A/B/C* in cells at different cell cycle stages (G1, S and G2M) for TRADITIOM LSC (live single cell) MCF7 –E2 (AI) arm from T0, early dormancy (1 month, 1mo), late dormancy (2 months, 2mo) to awakenings.
