# Supplementary material for: Long-term Multimodal Recording Reveals Epigenetic Adaptation Routes in Dormant Breast Cancer Cells
Source: Cancer Discov. 2024 Mar 26;14(5):866–89. doi: 10.1158/2159-8290.CD-23-1161 (PMC11061610; doi:10.1158/2159-8290.CD-23-1161)
Supplement: Supplementary Figure S26 — Validation of mass spectrometry and inhibitors’ activity [file cd-23-1161_supplementary_figure_s26_suppsf26.pdf]

**Supplementary Figure S26. Validation of mass spectrometry and inhibitors' activity**

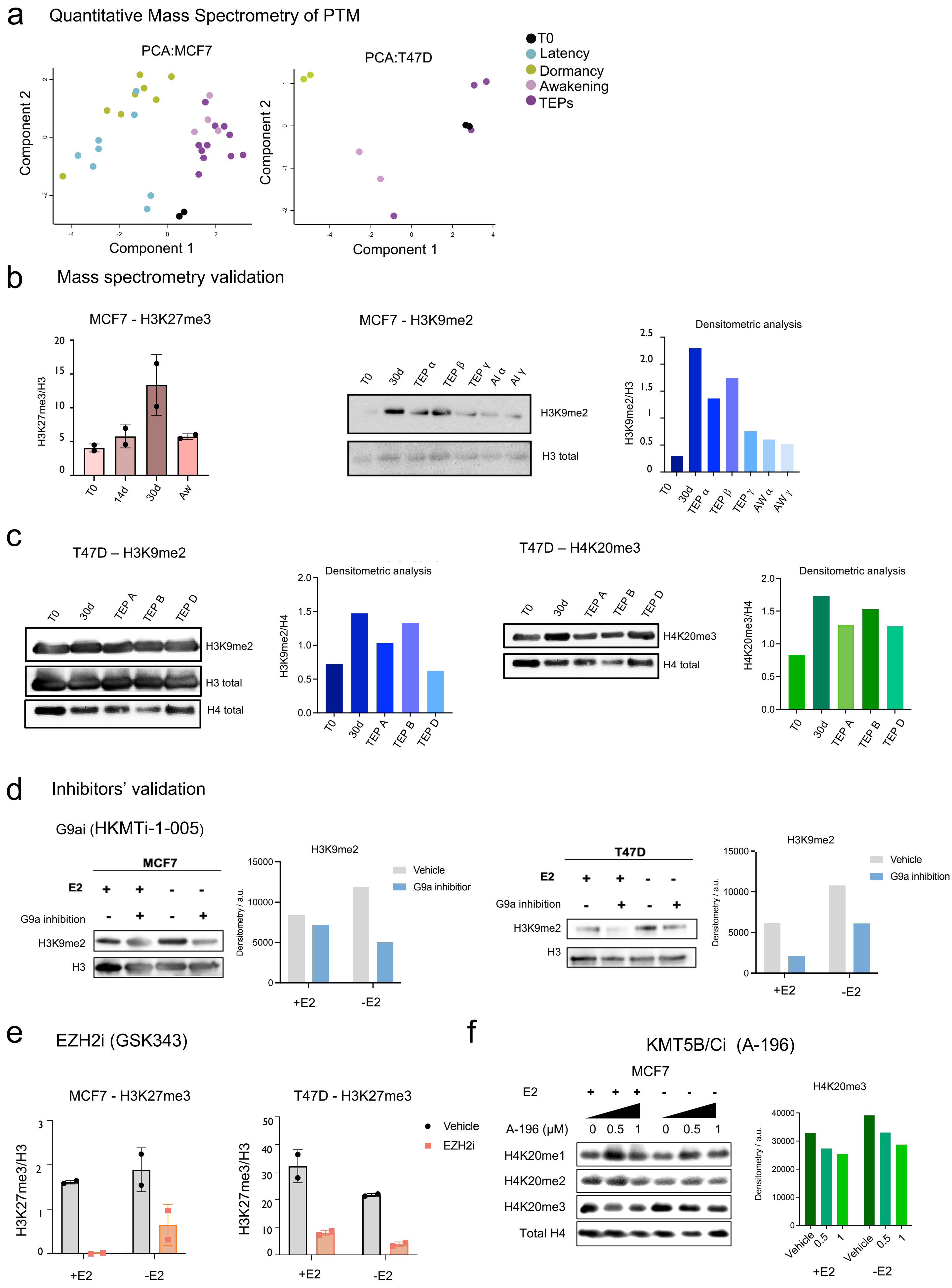

**Supplementary Figure S26. Validation of mass spectrometry and inhibitors' activity. a)** PCA plots for super-SILAC mass spectrometry of post-translational histone modifications in TRADITIOM dataset for MCF7 and T47D from T0 to TEPs (late progression). **b)** Mass spectrometry validation of TRADITIOM MCF7 dataset was done with ELISA for H3K27me3 (2 biological and 2 technical replicates) and with Western blotting for H3K9me2. Densitometric analysis (normalized over total H3) is shown in the bar plot. **c)** Mass spectrometry data of TRADITIOM T47D dataset were validated via Western blotting for H3K9me2 and H4K20me3. Densitometric analyses (normalized over total H4) are shown in bar plots. **d)** The effective inhibition by G9a inhibitor (HKMTi-1-005) was validated via Western blotting for both MCF7 (1mM, left panel) and T47D (1.5mM, right panel) in either oestrogen supplemented (+E2) or oestrogen deprived conditions (-E2). Densitometric analysis (normalized over total H3) is shown in bar plots. **e)** The effective inhibition by EZH2 inhibitor (GSK343) was validated via ELISA on two biological replicates for both MCF7 (1mM, left panel) and T47D (1.5mM, right panel) in either oestrogen supplemented (+E2) or oestrogen deprived conditions (-E2). **f)** The effective inhibition by KMT5B/C inhibitor (A-196) was validated via Western blotting for MCF7 cells (H4K20me1/me2/me3) in either oestrogen supplemented (+E2) or oestrogen deprived conditions (-E2). Densitometric analysis for target H4K20me3 modification is shown in bar plots.
