# Supplementary material for: Long-term Multimodal Recording Reveals Epigenetic Adaptation Routes in Dormant Breast Cancer Cells
Source: Cancer Discov. 2024 Mar 26;14(5):866–89. doi: 10.1158/2159-8290.CD-23-1161 (PMC11061610; doi:10.1158/2159-8290.CD-23-1161)
Supplement: Supplementary Figure S27 — Stratification of BC patients using the epigenetic dormancy signature [file cd-23-1161_supplementary_figure_s27_suppsf27.pdf]

Supplementary Figure S27. Stratification of BC patients using the epigenetic dormancy signature

a

Genes included in the epigenetic dormant signature
EZH2
EHMT2 (G9A)
KMT5C (SUV420H2)

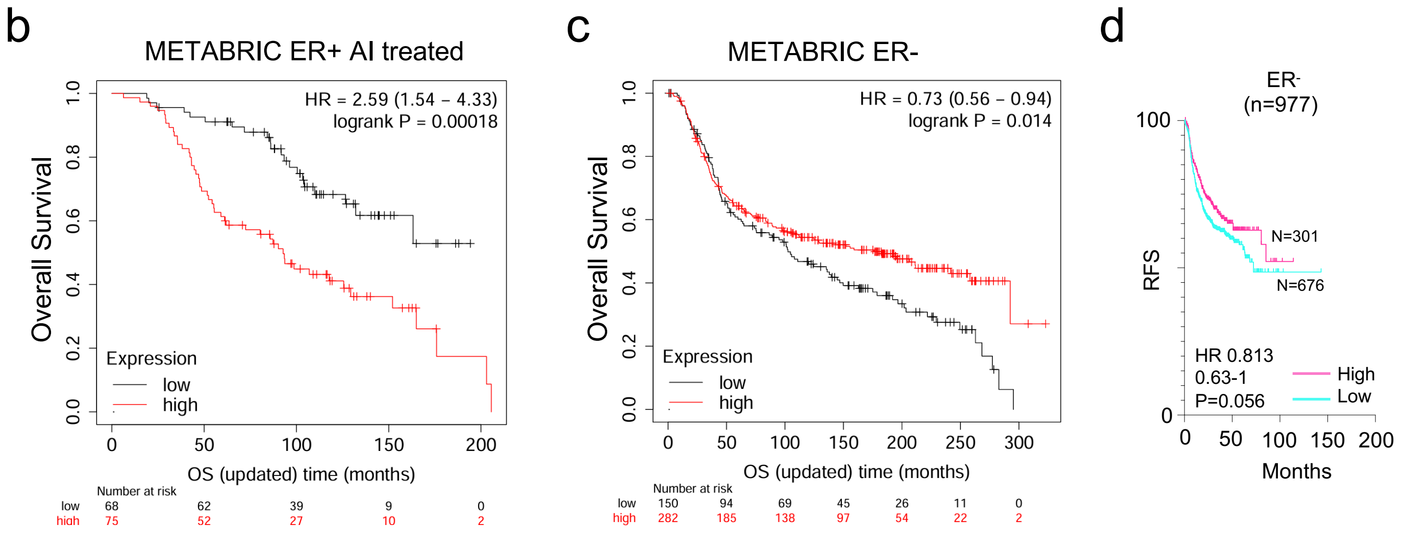

Supplementary Figure S27. Stratification of BC patients using the epigenetic dormancy signature. a) Genes included in the epigenetic dormancy signature **b)** Overall Survival (OS) curves for ER-positive (ER+) patients stratified based on the expression of the epigenetic dormancy signature (high vs low *G9a/EZH2/KMT5C* expression) **c)** Overall Survival (OS) curves for ER-negative (ER-) patients treated with Aromatase Inhibitors (AI) stratified based on the expression of the epigenetic dormancy signature **d)** Relapse free survival (RFS) curves for ER-negative (ER-) patients stratified based on the expression of the epigenetic dormancy signature.
